# *In silico* identification of coffee genome expressed sequences potentially associated with resistance to diseases

**DOI:** 10.1590/s1415-47572010000400031

**Published:** 2010-12-01

**Authors:** Samuel Mazzinghy Alvarenga, Eveline Teixeira Caixeta, Bárbara Hufnagel, Flávia Thiebaut, Eunize Maciel-Zambolim, Laércio Zambolim, Ney Sussumu Sakiyama

**Affiliations:** 1 Instituto de Biotecnologia Aplicada à Agropecuária, Universidade Federal de Viçosa, Viçosa, MGBrazil; 2 Empresa Brasileira de Pesquisa Agropecuária, Embrapa Café, Brasília, DFBrazil; 3 Departamento de Fitopatologia, Universidade Federal de Viçosa, Viçosa, MGBrazil; 4 Departamento de Fitotecnia, Universidade Federal de Viçosa, Viçosa, MGBrazil

**Keywords:** *Coffea*, data mining, ESTs, genomics, *in silico*, bioinformatics

## Abstract

Sequences potentially associated with coffee resistance to diseases were identified by *in silico* analyses using the database of the Brazilian Coffee Genome Project (BCGP). Keywords corresponding to plant resistance mechanisms to pathogens identified in the literature were used as baits for data mining. Expressed sequence tags (ESTs) related to each of these keywords were identified with tools available in the BCGP bioinformatics platform. A total of 11,300 ESTs were mined. These ESTs were clustered and formed 979 EST-contigs with similarities to chitinases, kinases, cytochrome P450 and nucleotide binding site-leucine rich repeat (NBS-LRR) proteins, as well as with proteins related to disease resistance, pathogenesis, hypersensitivity response (HR) and plant defense responses to diseases. The 140 EST-contigs identified through the keyword NBS-LRR were classified according to function. This classification allowed association of the predicted products of EST-contigs with biological processes, including host defense and apoptosis, and with molecular functions such as nucleotide binding and signal transducer activity. Fisher's exact test was used to examine the significance of differences in contig expression between libraries representing the responses to biotic stress challenges and other libraries from the BCGP. This analysis revealed seven contigs highly similar to catalase, chitinase, protein with a BURP domain and unknown proteins. The involvement of these coffee proteins in plant responses to disease is discussed.

## Introduction

Coffee (*Coffea* spp.), which is produced in more than 60 countries, is one of the most important exportation products in the world. However, its production is frequently adversely affected by diseases and pests. Plant resistance genes (*R* genes) are a very important group of genes that have been used by breeders to develop resistant cultivars as part of strategies for disease control.

Flor (1942) proposed a highly specific recognition system between plant *R* gene products and pathogen elicitor proteins (Avr effectors). In Flor's gene-for-gene theory, the plant with a dominant *R* gene recognizes the pathogen's corresponding dominant *Avr* gene. The presence of the *Avr* gene makes the pathogen non-virulent if the plant has the appropriate *R* gene. If there is no *R* gene in the plant and/or no *Avr* gene in the pathogen, then there is no recognition and disease is initiated (Jia *et al.*, 2000; Deslandes *et al.*, 2003; Belkhadir *et al.*, 2004).

The interaction between the *R* and *Avr* gene product triggers a signal transduction pathway involving several proteins that leads to a plant defense response (Caplan and Dinesh-Kumar, 2006). Some of the proteins induced in response to infection by pathogens include thaumatin, a pathogenesis-related protein, chalcone synthase, a key enzyme in the biosynthesis of diverse flavonoids involved in disease resistance, chitinases and glucanases, which exhibit antifungal activity by attacking components in fungal cell walls, and proteins such as polyphenol oxidases, glucosyl transferases and phytoalexins, known to be involved in plant defense against pests and pathogens.

Plant *R* gene identification and isolation has increased in the last decade (Richter and Ronald, 2000) and the sequencing of several plant genomes has produced a large amount of information on the functions of gene products, and the regulation of biosynthetic and signal transduction pathways (Staskawicz *et al.*, 1995). *R* genes encode at least five types of proteins (R proteins) (Dangl and Jones, 2001) that can be classified according to their structural characteristics. The first R gene to be cloned was the tomato *Pto* gene, which codes for a serine/threonine kinase protein and confers resistance to races of *Pseudomonas syringae* carrying the *AvrPto* avirulence gene (Martin *et al.*, 1993). This gene belongs to the first class (class 1) of R proteins.

Class 2 R proteins include the tomato *Cf9* gene, which confers resistance to *Cladosporium fulvum* and encodes transmembrane proteins, the extracellular N-terminal region of which has leucine-rich repeats (LRR) (Jones *et al.*, 1994). However, these proteins have no significant intracellular region that can serve as a signaling component to activate plant defense mechanisms (Ellis and Jones, 1998).

Class 3, represented by the rice *Xa21* gene (which confers resistance to *Xanthomonas oryzae*), includes proteins with a classic receptor-kinase, one extracellular LRR region, one transmembrane region and a cytosolic serine/threonine kinase domain (Song *et al.*, 1995; Ellis and Jones, 1998).

Most of the resistance genes code for proteins with a variable N-terminal domain followed by a tripartite nucleotide binding site (NBS) and an LRR region (Baker *et al.*, 1997; Ellis *et al.*, 2000; Dangl and Jones, 2001). Based in their N-terminal domain, these proteins can be classified in two groups. The TIR-NBS-LRR group (class 4) has a sequence similar to the Toll protein of *Drosophila* and to the Interleukin-1 Receptor in mammals (TIR). This group TIR-NBS-LRR includes the tobacco *N* gene (which confers resistance to *tobacco mosaic virus -* TMV), the genes *L6* and *M* (resistance to rust) from flax and the genes *RPP5*, *RPP1* (both conferring resistance to *Peronospora parasitica*) and *RPS4* (resistance to *Pseudomonas syringae*) from *Arabidopsis* (Ellis and Jones, 1998; Gassmann *et al.*, 1999).

The other group (class 5) includes non-TIR-NBS-LRR proteins that lack the TIR sequence (Meyers *et al.*, 1999; Pan *et al.*, 2000; Cannon *et al.*, 2002; Richly *et al.*, 2002) and/or contain a leucine zipper domain (LZ) or a coiled coil (CC) domain in their N-terminal region (Pan *et al.*, 2000). CCs are structures formed by 2-5 helixes that display a distinct folding of the amino acid side chains at the helix-helix interface (Lupas, 1996). The CC structure has an organized repetition of seven amino acid residues, two of which have hydrophobic side chains that form an interface for interaction between coils (Young, 2000).

The CC-NBS-LRR class includes the *RPM1* and *RPS2* genes, and the LZ-NBS-LRR class includes the *ArabidopsisRPS5* and tomato *Prf* genes (both confer resistance to *P. syringae*), the *ArabidopsisRPP8* gene (resistance to *P. parasitica*), the tomato *Mi* gene (resistance to the nematode *Meloidogyne incognita*) and the potato *Rx1* gene (resistance to *potato virus* X – PVX) (Ellis and Jones, 1998; Bendahmane *et al.*, 1999). The tomato *I2*, maize *Rp1-D*, lettuce *RGC2* and pepper *Bs2* genes contain neither the TIR nor the LZ region (Tai *et al.*, 1999). In addition to these two regions, some R gene products contain a conserved domain of unknown function, referred to as GLPLAL, between the NBS and LRR regions (Dinesh-Kumar *et al.*, 2000).

ESTs similar to the NBS-LRR class of R proteins have been found in coffee. By using several degenerate primers for two conserved motifs within the NBS regions of R genes from different species, Noir *et al.* (2001) amplified and cloned sequences from nine distinct motifs of NBS proteins in coffee. These authors identified 18 RGAs (Resistance Gene Analogs) in a single coffee accession, a finding that suggested the presence of a large number of R genes in the coffee genome.

Fernandez *et al.* (2004) identified two coffee ESTs highly similar to the proteins DND1 (Defense, No Death) and NDR1 (Non race-specific Disease Resistance), which are resistance signaling components in *Arabidopsis thaliana*. DND1 protein is an ion channel involved in the hypersensitivity reaction signaling pathway whereas NDR1 is a key-component in the signaling pathway of several CC-NBS-LRR proteins (Silva *et al.*, 2006). Guzzo (2004) isolated coffee genes with functions related to plant defense against phytopathogens. These genes are involved in processes such as the hypersensitivity reaction, programmed cell death, anti-microbial protein synthesis and controlled protein degradation. Lin *et al.* (2005) identified coffee genes with putative functions related to disease resistance (such as TIR-NBS-LRR proteins), pathogenesis and other activities.

A better understanding of the mechanisms of disease resistance in coffee plants and the development of molecular tools for identifying these mechanisms may be useful in genetic breeding programs for this crop plant. In this regard, genomic analysis can be a powerful ally in investigating *R* genes and plant-pathogen interactions. The recent application of genome technology to plants has generated considerable information and has provided DNA sequence databases that have allowed the identification of genetic factors related to plant characteristics of agricultural interest.

The use of technologies such as expressed sequence tags (ESTs), which are short sub-sequences of transcribed cDNA sequences, has greatly reduced the time required to locate and describe plant genes. ESTs can be used to identify gene transcripts and are instrumental in gene discovery and gene sequence determination (Adams *et al.*, 1991). Due to their efficiency, ESTs have been widely in creating public databases (Wolfsberg and Landsman, 1997). In plants, ESTs were initially reported for *Arabidopsis* (Höfte *et al.*, 1993) and rice (Yamamoto and Sasaki, 1997), and this was rapidly followed by similar projects for corn (Gai *et al.*, 2000), soybean (Shoemaker *et al.*, 2002), wheat (Lazo *et al.*, 2004), potato (Ronning *et al.*, 2003) and cotton (Udall *et al.*, 2006). The data provided by such studies can be readily accessed via the EST database (dbEST) of the National Center for Biotechnology Information (NCBI), and has facilitated the identification of genes responsible for selected agronomical traits and their subsequent manipulation by molecular genetic techniques.

In the Brazilian Coffee Genome Project (BCGP), approximately 33,000 unigenes were identified in 214,964 ESTs obtained from 37 cDNA libraries of *Coffea arabica*, *Coffea canephora* and *Coffea racemosa* under different physiological conditions. The clustering of these ESTs yielded 17,982 EST-contigs and 32,155 singlets (Vieira *et al.*, 2006). In this work, we used *in silico* analysis to screen the BCGP database for the genes of proteins potentially involved in coffee resistance to diseases.

## Material and Methods

###  Data mining

All of the data from the BCGP, as well as various bioinformatics tools, were provided by the Laboratório de Genômica e Expressão (LGE) and by Embrapa Recursos Genéticos e Biotecnologia (Cenargen).

Initially, we searched the literature to identify proteins related to the mechanisms of plant disease resistance. These proteins were subsequently used as keywords to mine the ESTs in the BCGP. Data mining was done using Gene Projects, a sequence management and manipulation system that is part of the LGE bioinformatics platform (Carazzolle *et al.*, 2007). Based on the keywords found, we ran a search that targeted the annotation of each EST in the LGE database. The ESTs identified in each keyword-based search were placed in virtual folders referred to as “projects”. Each project was identified by a keyword and contained its related ESTs. The projects created were: Chitinase, Cytochrome P450, Glucanase, Glucosyltransferase, HSP (Heat Shock Protein), Hypersensitive, Importin, NBS-LRR (nucleotide binding site-leucine rich repeat), Pathogenesis, Polyphenoloxidase, Phytoalexin, Resistance, Thaumatin and Chalconesyntase. The ESTs of each project were clustered with the CAP3 assembly program (Huang and Madan, 1999) to form EST-contigs that corresponded to consensus sequences with improved length and quality. These EST-contigs were compared to the GenBank nr sequence database (version of August 26, 2002) by BLAST (BLASTX version 2.2.4) (Altschul *et al.*, 1990) with the BLOSUM 62 scoring matrix. The EST clustering and BLAST searches were done through Gene Projects. Each EST-contig annotation was examined in order to obtain relevant information about the putative disease resistance genes. Only EST-contigs with E-values < e^-20^ and scores > 100 were considered for further analysis.

###  Functional characterization

Functional characterization of the selected EST-contigs from the NBS-LRR Project was done with the Blast2GO software (Conesa *et al.*, 2005). This program finds sequences similar to each EST-contig by Blast searching against the NCBI nr database. The maximum E-value from the best blast hit was set to 1e^-10^ and the minimum alignment size (HSP length) was set to 33. Based on the BlastX results, the Blast2GO extracted terms from the Gene Ontology (GO) for each EST-contig. The distribution of GO terms was analyzed at level 3 of the Directed Acyclic Graphs (DAGs). The three categories of electronically designed terms were Molecular Function, Biological Process and Cellular Component. Conserved domains were identified by screening the EST-contigs with InterProScan from the EBI (European Bioinformatics Institute). InterPro is a database that contains information about domains, motifs and regions that are conserved in protein families. The data for InterPro are obtained from other databases that include PROSITE, PRINTS, ProDom, Pfam, SMART, TIGRFAMs, PIRSF, UPERFAMILY and PANTHER. The last step of the program consisted of annotating the ECs (Enzyme Codes) and searching for KEGG (Kyoto Encyclopedia of Genes and Genomes) metabolic maps for the EST-contigs.

###  Fisher's exact test

Fisher's exact test (Fisher, 1922), available from the Cenargen Bioinformatics platform, was used to compare the levels of contig expression between libraries generated following exposure to biotic stress and other libraries from the BCGP. The major libraries generated after biotic stress were RM1 (leaves infected with leaf miner and coffee leaf rust), NS1 (roots infected with nematodes) and RX1 (stems infected with *Xylella* spp.) while the other libraries consisted of the remaining coffee genome libraries (Vieira *et al.*, 2006). A value of p = 0.05 indicated significance.

## Results and Discussion

###  Data mining

A total of 11,300 ESTs were mined and deposited in 14 projects. The projects CytochromeP450, Resistance and Chitinase contained the highest number of ESTs, with 2,441 (21.6%), 1,864 (16.5%) and 1,855 (16.4%) ESTs, respectively (Table 1). The projects with the smallest number of ESTs were Phytoalexin, Polyphenoloxidase and Hypersensitive, with 12 (0.10%), 67 (0.59%) and 86 (0.76%) ESTs, respectively.

With the keyword NBS-LRR it was possible to form 140 EST-contigs. The LRR (Leucine-Rich Repeat) domain participates in the interaction of the R protein with its related Avr gene product (elicitor). Sequence variability within the interstitial leucine residues can affect ligand binding and may confer different recognition specificities to each Avr factor (Parniske *et al.*, 1997).

In several plant-pathogen systems, variation in the LRR region sequence is responsible for different recognition or resistance specificities (Parniske *et al.*, 1997; Thomas *et al.*, 1997; Botella *et al.*, 1998; Wang *et al.*, 1998; Ellis *et al.*, 1999). The NBS (nucleotide binding site) domain is believed to participate in the activation of signal transduction compounds, leading to pathogen-specific resistance responses (Aarts *et al.*, 1998; Feys and Parker, 2000; Van Der Biezen *et al.*, 2000). Using the NBS-LRR bait, EST-contigs similar to disease resistance proteins, NBS-LRR proteins, chitinases and other proteins were identified (Table S1). The conserved domains found in the EST-contigs included pfam00931 (NB-ARC domain), COG4886 (LRR protein), cd00116 (LRR_RI, leucine-rich repeats, ribonuclease inhibitor (RI)-like subfamily), smart00220 (S_TKc, serine/threonine protein kinases) and pfam01582 (TIR domain).

The keyword Resistance was used as a general term to obtain ESTs related to plant defense mechanisms and resulted in the identification of 300 EST-contigs that included several classes of disease resistance proteins, such as CC-NBS-LRR, TIR-NBS-LRR and nematode and virus resistance genes (Table S2). The conserved domains observed in the EST-contigs included COG4886 (LRR protein), pfam00931 (NB-ARC domain) and pfam01582 (TIR domain).

Mining with the term Hypersensitive Reaction (HR) yielded eight EST-contigs. The HR involves an extreme cellular response that may cause a high degree of resistance to disease. The HR is an induced defense response that results in the death of a limited number of host cells surrounding the sites of pathogen infection and culminates with the interruption of pathogen multiplication and growth on plant tissues. The HR occurs in response to the recognition of infection by the host, as a consequence of incompatibility between plant and pathogen (Pascholati and Leite, 1994). Of the eight EST-contigs identified, six encoded proteins that were related to the HR (Table S3).

The Pathogenesis project contained 61 EST-contigs that included proteins related to pathogenesis, chitinases, thaumatin family proteins and other proteins. The conserved domains found in the EST-contigs included cd00035 (chitin binding domain), cd00325 (chitinase glyco hydro 19 - glycoside hydrolase family 19 chitinase domain) and pfam00407 (pathogenesis-related protein Bet v I family) (Table S4).

45 EST-contigs were identified with the keyword Chitinase. Chitinases form a large group of enzymes with diverse structures and functions, some of which are related to the resistance of several plant species to pathogens (Sahai and Manocha, 1993; Jackson and Taylor, 1996). These enzymes are among the most expressed genes in coffee (Lin *et al.*, 2005). In a manner functionally similar to glucanases (see below), chitinases weaken the fungus cell wall by hydrolyzing chitin, an N-acetylglucosamine polymer. This hydrolysis results in cell lysis and death (Lin *et al.*, 2005). Analysis of the EST-contigs identified in the Chitinase project revealed the presence of chitinases with several conserved domains, including pfam00704 (glyco hydro 18), cd00035 (ChtBD1, chitin binding domain), cd00325 (chitinase glyco hydro 19, Glycoside hydrolase family 19 chitinase domain) and COG3469 (chitinase) (Table S5).

The CytochromeP450 project contained 144 EST-contigs (Table S6). Cytochrome P450 is involved in cellular oxidation pathways. The proteins encoded by these genes are involved in the biosynthesis of compounds related to defense, such as the *Arabidopsis* gene PAD3, which is required for the synthesis of camalexin during resistance to *Alternaria brassicicola* (Zhou *et al.*, 1999). Takemoto *et al.* (1999) showed that the product of the gene CYP82E1 (cytochrome P450) may be involved in the resistance of tobacco to *Pseudomonas syringae*. Qi *et al.* (2006) demonstrated that the enzyme AsCYP51H10 (a member of the cytochrome P450 family) participates in the production of antimicrobial compounds (avenacins) that confer resistance to diseases in oatmeal.

The keyword Glucanase, derived from the enzyme β1,3-glucanase, identified 88 EST-contigs. This enzyme hydrolyzes β1,3-glucan present in the fungus cell wall, and eventually leads to cell lysis and death (Selitrennikoff, 2001). The extracellular forms of this protein apparently act in the early stages of plant defense by exerting a direct fungicidal action on the hyphae of invading fungi. The release of eliciting oligosaccharides from the fungus wall can activate other local or systemic mechanisms of plant resistance. In contrast, the intracellular forms of β1,3-glucanase apparently act later in plant defense reactions (Boller and Métraux, 1988). The antifungal activity of glucanases has been demonstrated in several cellular enzymatic assays (Stintzi *et al.*, 1993), as well as in transgenic plants (Jach *et al.*, 1995). The EST-contigs identified in the Glucanase project included xyloglucan endotransglycosylase proteins, cellulase, β-1,3-glucanase, proteins from the glycosyl hydrolase family and proteins involved in brassinosteroid regulation (Table S7).

The project HSP (Heat Shock Protein) contained 30 EST-contigs. An increase in heat shock protein expression protects animals and plants against environmental stress. HSP90, a chaperone protein required for several metabolic defense pathways, contributes to the accumulation of resistance proteins in cells (Dreher and Callis, 2007). Analysis of the EST-contigs identified here revealed the presence of several classes of HSP proteins containing the conserved domains pfam00012 (HSP70), cd00298 (alpha-crystallin-Hsps), COG0071 (IbpA, molecular chaperone), cd00189 (TPR, tetratricopeptide repeat domain) and PRK00290 (dnaK, molecular chaperone DnaK) (Table S8).

The project Thaumatin identified 16 EST-contigs associated with thaumatin and pathogenesis-related (PR) proteins. Thaumatin is a PR protein with fungicidal activity against a large number of plant and human pathogens (Selitrennikoff, 2001). Although the precise mechanism of action of this protein is still unclear, various observations indicate that it has an important role in fungus death (Selitrennikoff, 2001). Thaumatin alters the permeability of the fungus cell wall but has little or no effect on the protoplast (Roberts and Selitrennikoff, 1990). This protein binds 1,3 β-glucan and has 1,3 β-glucanase activity *in vitro* (Grenier *et al.*, 1993; Trudel *et al.*, 1998). In tobacco, thaumatin interferes with the regulation of cell wall assembly in *Saccharomyces cerevisiae* (Yun *et al.*, 1997, 1998). Analysis of the EST-contigs identified in the Thaumatin project revealed the presence of the conserved domains Smart 00205 (THN) and pfam00314 (Thaumatin family) (Table S9).

The project Phytoalexin was consisted of three EST-contigs, all of which were annotated for the phytoalexin-deficient 4-1 protein of *Solanumtuberosum* (Table S10). Phytoalexins are synthesized by and accumulate in plant cells after microbial infection (Stoessl, 1986). Phytoalexins inhibit elongation of the germinative tube and radial colony growth, in addition to causing collapse of the membrane system and electrolyte loss. Resistant plants produce high levels of phytoalexins when compared to susceptible ones. The concentration of phytoalexin increases in parallel with that of key enzymes, *e.g.*, chalcone synthase, involved in its biosynthesis (Pascholati and Leite, 1994).

Five EST-contigs were identified with the keyword Chalconesynthase (Table S11). Three EST-contigs had the annotation chalcone synthase, one was annotated for aldo-keto reductase and one for 3-oxoacyl-(acyl-carrier-protein) synthase III. Chalcone synthase, an enzyme that regulates the biosynthesis of phenylpropanoids, catalyzes the first reaction of the biosynthetic pathway of flavonoids and isoflavonoids. Elicitors cause fast transient stimulation of chalcone synthase gene transcription as a basal event in the response to phytoalexins (Ryder *et al.*, 1984).

Four EST-contigs were identified with the keyword Polyphenoloxidase (Table S12). The most relevant annotations were catechol oxidase and polyphenol oxidase. Although the physiological function of polyphenol oxidase in plant cells is still unclear, most reports indicate that this enzyme functions in plant defense against attacks by pathogens and insects. This proposed involvement in defense is based on the enzyme's ability to oxidize phenolic compounds when the tissue is damaged. In this situation, rupture of the cellular compartment containing polyphenoloxidase (plastids) brings the enzyme into contact with phenolic compounds released by rupture of the vacuole (Melo *et al.*, 2006).

The project Importin consisted of 10 EST-contigs, six of which were annotated as importin α (Table S13). Importin α mediates the importation of cytosolic proteins into the nucleus. Plant importin α binds to virulence factors in *Agrobacterium tumefaciens* and several phytopathogenic viruses (Hermsmeier *et al.*, 2001).

The project Glucosyltransferase generated 125 EST-contigs. Glycosyl transferases catalyze the transfer of glucose residues to several substrates and regulate the activity of compounds that have important functions in plant defense against pathogens, such as salicylic acid (Chong *et al.*, 2002). The EST-contigs identified in the project Glucosyltransferase included proteins from the glucosyl transferase family, saccharose synthase and glucosyl transferases induced by cold (Table S14).

**Figure 1:**
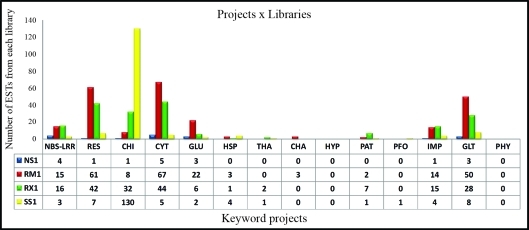
Number of ESTs from NS1 (roots infected with nematodes), RM1 (leaves infected with leaf miner and coffee leaf rust), RX1 (stems infected with *Xylella* spp.) and SS1 (well-watered field plants) libraries that were present in the created projects. CHA – chalconesynthase, CHI – chitinase, CYT – cytochrome P450, GLT – glucosyltransferase, GLU – glucanase, HYP – hypersensitive, IMP – importin, NBS-LRR – nucleotide binding site-leucine rich repeat, PAT – pathogenesis, PFO – polyphenoloxidase, PHY – phytoalexin, RES – resistance and THA – thaumatin.

Figure 1 and Table S15 show the number of ESTs identified from the different libraries in each project. The libraries analyzed were NS1 (roots with nematodes), RM1 (leaves with coffee leaf miner and coffee leaf rust), RX1 (branches with *Xylella*) and SS1 (normal tissues). The libraries NS1, RM1 and RX1 were chosen because they were under biotic stress and therefore represented a potential source of ESTs involved in plant defense against stress. The SS1 library served as a control to identify genes that were expressed when the plant was not under stress.

This analysis showed that the RM1 library was best represented in the projects Resistance, Cytochrome P450, Glucanase and Glucosyltransferase. This library was also well represented in the projects NBS-LRR and Importin. These observations confirmed the potential importance of RM1 library ESTs in plant defense against coffee leaf miner and coffee leaf rust.

The RX1 library was also well represented in the projects Resistance, Cytochrome P450 and Glucosyltransferase. RX1 was best represented in the projects NBS-LRR, Pathogenesis, and Importin. This result indicates that ESTs mined in this library are important in coffee plant defense against *Xylella* because they are potentially related to resistance process against this pathogen.

The projects Resistance, Chitinase, Cytochrome P450 and Glucosyltransferase contained the highest number of ESTs from the libraries of interest. The search term “resistance” is a good general term for plant defense mechanisms, and it was expected that the three libraries obtained under stress conditions (NS1, RM1, RX1) would be well-represented by ESTs for stress-related genes. This was indeed the case for the RM1 and RX1 libraries, whereas the NS1 library had only one EST in these projects while the SS1 library had seven. The low number of ESTs in the NS1 library may reflect a weak expression of genes related to the keyword “resistance”, or may indicate that the genes were not sampled. Further studies may explain these results.

Chitinases, which are related to the resistance of several plant species to pathogens (Sahai and Manocha, 1993; Jackson and Taylor, 1996), were substantially expressed in normal conditions. This finding agrees with Lin *et al.* (2005), who showed that chitinases were among the most expressed genes in coffee. According to these authors, the fact that the chitinases are highly expressed and represented by an extended gene family in coffee may reflect a greater need for fungal resistance. This need may be related to the perennial nature of coffee and to the fact that this plant is a tropical species and may therefore be exposed to a large number of pathogens.

Plant cytochrome P450 enzymes are involved in a large number of biosynthetic reactions related to the formation of fatty acids, hormones, defense compounds and other molecules. Up to August 2009 (the last available update) 11,294 cytochrome P450 sequences, distributed in 977 families, had been identified; 3,284 of these sequences (excluding variants and pseudogenes) were from plant species (see Cytochrome P450 homepage in Internet Resoures). Hence, a large number of the cytochrome P450 ESTs identified here may be involved in biological processes unrelated to plant defense against diseases.

No ESTs were identified by the Hypersensitive and Phytoalexin projects in any of the libraries analyzed. This suggests that genes related to these terms are either not expressed in these libraries or that their expression was too low to be detected.

The 979 EST-contigs potentially associated with coffee plant resistance to diseases identified here provide a useful database for expanding our understanding of coffee plant resistance to pathogens and pests. The domains detected in the sequences described here served as: (1) recognition sites for protein-protein interactions, (2) effector binding sites to produce protein conformational changes, (3) sites for ion signal modulation that resulted in target regions alterations and consequent protein activation or inactivation, and (4) recognition sites for elicitor molecules released by pathogens. In addition, the EST-contigs identified here may be useful as probes in the physical mapping of coffee and as candidate genes for the development of molecular markers and the identification of RGAs (Resistance Genes Analogs) in coffee breeding programs.

###  Functional categorization

The NBS-LRR project generated 826 ESTs that, after clustering, resulted in 160 EST-contigs and 243 singlets. 140 of these EST-contigs had a score > 100 and an E-value < e^-20^. Most of the 140 EST-contigs consisted of two or three ESTs (Figure S1). The size of the EST-contigs ranged from 469 bp (EST-contig 49: putative NBS-LRR type disease resistance protein of *Prunus persica*) to 3281 bp (EST-contig 50: putative leucine-rich repeat transmembrane, putative protein kinase of *Gossypium hirsutum*) (Table S1).

The best hits for species in the BlastX searches for EST-contigs were for *Vitis vinifera* and *Populus trichocarpa* (Figure S2). A recent study of the evolution and genomic composition of *C. canephora* revealed considerable conservation of the microcollinearity between this species and *V. vinifera* (Guyot *et al.*, 2009). These authors also reported a high level of conservation between the genomes of *C. canephora* and other Dicotyledon species such as *Solanum lycopersicon* and *Populus trichocarpa*. Species from the Asterid I group, such as *Solanum* sp. and *Lycopersicon* sp., known to be related to coffee (Lin *et al.*, 2005), also showed a large number of blast hits.

BlastX of the EST-contigs done through Blast2GO resulted in hits with annotations for NBS-LRR, CC-NBS-LRR, TIR-NBS-LRR, disease resistance protein, leucine-rich repeat transmembrane protein kinase, leucine-rich repeat receptor-like protein kinase and others. The average E-value for the 20 best BlastX hits ranged from e^-10^ to e^-180^ (Figure S3). The average similarity level for the 20 best hits ranged from 43% to 99% (Figure S4). These data show that the results obtained in the annotation were significant and thus highly reliable.

Of the 140 EST-contigs analyzed, 89 were associated with a conserved domain. Analysis of the InterProScan database revealed similarities with domains such as IPR002182 (PF00931 – domain NB-ARC), IPR001611 (PF00560 – domain LRR), IPR007271 (PTHR10231 – nucleotide*-*sugar transporter) and IPR000719 (PS50011 – domain protein chitinase), all of which have functions related to plant defense against pathogens. No proteins with enzymatic activity (no attached EC) and no metabolic maps (no attached KEGG map) were associated with these EST-contigs.

GO functional classification terms were retrieved for 138 EST-contigs (98.6%): one contig had 11 GO terms, 39 (27.9%) had only one term and two (1.42%) had no term (Figure S5). Since most of the biological functions for the DNA sequences and corresponding proteins were inferred by electronic annotation of the GO terms (Figure S6) it was necessary to screen several databases in order to obtain the greatest amount of information before depositing the sequence in a database.

**Figure 2:**
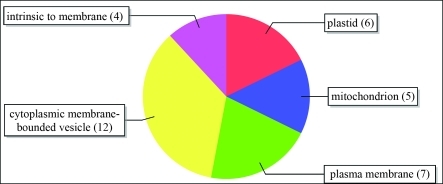
Distribution of GO terms in the Cellular Component category, level 3.

The Cellular Component category contained terms such as “intrinsic to the membrane” and “plasma membrane” (Figure 2). Known R genes, such as *Cf9* from tomato, which confers resistance to *Cladosporium fulvum*, code for transmembrane proteins in which the extracellular N-terminal region consists of an LRR domain (Jones *et al.*, 1994).

**Figure 3:**
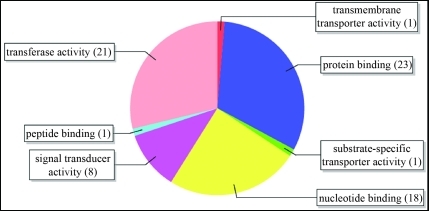
Distribution of GO terms in the Molecular Function category, level 3.

The terms associated with the Molecular Function category included “signal transducer activity”, “nucleotide binding” and others (Figure 3). Nucleotide binding activity has been associated with the NBS domain, which has the molecular function of binding ATP or GTP in various organisms (Saraste *et al.*, 1990; Traut, 1994). The N-terminal regions, together with the NBS domain, are believed to participate in the activation of signal transduction pathways involved in pathogen-specific resistance responses (Aarts *et al.*, 1998; Feys and Parker, 2000; Van Der Biezen *et al.*, 2000).

**Figure 4:**
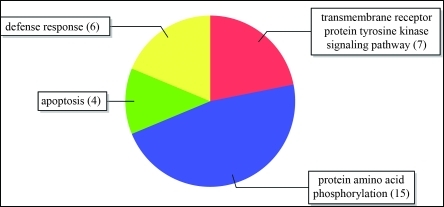
Distribution of GO terms in the Biological Process category, level 3.

The Biological Process category contained terms such as “defense response”, “apoptosis” and “tyrosine kinase protein transmembrane receptor signaling pathway” (Figure 4). The *Pto* gene from tomato encodes a serine/threonine kinase protein that confers resistance to the races of *Pseudomonas syringae* that carry the avirulence gene *AvrPto* (Martin *et al.*, 1993). The gene *Xa21* (resistance to *Xanthomonas oryzae*) from rice, on the other hand, encodes proteins with the classic receptor-kinase format, *i.e.*, an extracellular LRR region, a transmembrane region and a cytosolic serine/threonine domain (Song *et al.*, 1995; Ellis and Jones, 1998).

###  Fisher's exact test

In Fisher's exact test, the p value represents the probability of a given cluster having equal expression in both of the groups analyzed, *i.e.*, if p < 0.05 then there is a significant difference in the expression of a given cluster between the two groups analyzed. Fisher's exact test identified seven contigs that differed in expression between the two groups analyzed, *i.e.*, biotic stress challenged libraries versus the remaining coffee genome libraries. A search for similarity using BlastX resulted in hits that included catalase, chitinase, a protein with a BURP domain and unknown proteins (Table 2).

In plant defense mechanisms, pathogen recognition is followed by an oxidative burst that triggers the rapid generation and accumulation of reactive oxygen species (ROS). During biotic and abiotic stress ROS may have two very different roles: the exacerbation of damage and the activation of defense responses (Dat *et al.*, 2000). Hydrogen peroxide, a powerful and potentially harmful ROS, damages both plant cells and pathogens (Allan and Fluhr, 1997). Catalase, an enzyme that protects cells from the toxic effects of peroxides, catalyzes the conversion of hydrogen peroxide to water and molecular oxygen (Willekens *et al.*, 1997). Catalase also uses hydrogen peroxide to oxidize toxins, including phenols, formic acid, formaldehyde and alcohols (Resende *et al.*, 2003). Dixon *et al.* (1994) showed that in soybean (*Glycine max*) catalase blocked the accumulation of phytoalexin glyceolin elicited by *Verticillium dahlia* Kleb. Treatment with catalase also blocks the induction of cell death in several systems in response to various avirulence signals (Lamb and Dixon, 1997). Catalase activity usually increases in response to high concentrations of hydrogen peroxide during virus infection (Riedle-Bauer, 2000). Lignin deposition tends to increase the resistance of plant cell walls to digestive enzymes of pathogens. In plant defense mechanisms, catalase acts in conjunction with peroxidases to accelerate the oxidation of phenolic compounds that are precursors in lignin synthesis (Margis-Pinheiro *et al.*, 1993). Based on these considerations, we inferred that contigs 8478 and 9073, which putatively encode catalases, are somehow involved in plant defense against pathogens.

Contig 14592 showed high similarity to a class III chitinase from *Coffea arabica* that contains a conserved domain called hevamine. This conserved domain hydrolyzes the linear polysaccharide chains of chitin and peptidoglycan and is therefore important for defense against pathogenic bacteria and fungi. Class III chitinases belong to family 18 of glycosyl hydrolases (Hamel e*t al.*, 1997) and include bifunctional enzymes with lysozyme activity (Jekel *et al.*, 1991). As mentioned earlier, chitinases degrade chitin and are widely distributed in many species of higher plants (Park *et al.*, 2002). Much of the evidence for the suggested roles of chitinases in plant defense has been based on dramatic and rapid enhancement of enzyme levels in the HR during induced host resistance, and in pathogen-infected tissues (Punja and Zhang, 1993).

The BlastX result for contigs 13986 and 431 was, in both cases, a member of the cysteine protease inhibitor family from *Arabidopsis thaliana*. However, the E-values of 0.003 and 0.005 for contigs 13986 and 431, respectively, were above the threshold usually adopted in similarity searches. This finding indicates that these contigs encode a *Coffea* protein with no significant similarity to any protein present in the NCBI nr database at the moment. The BlastX results for contig 10650 indicated that there was a protein in the database that had significant similarity to the product of this contig. However, the identity and function of this protein are unknown. Contig 13908 was highly similar to a BURP domain from *Solanum tuberosum*; BURP domains are believed to have important, fundamental functional roles (Hattori *et al.*, 1998), although no role in defense has yet been attributed to this domain.

In conclusion, the results of this study provide important information on resistance genes in the coffee genome since the terms identified in the GO analysis are probably related to plant defense mechanisms. The information generated by this genomic analysis also expands our understanding of the possible defense responses triggered in coffee plants in response to pathogens.

## Supplementary Material

The following online material is available for this article:

Table S1 Table S1 - EST-contigs with E-values < e^-20^ and scores > 100 obtained in the project NBS-LRR, and their blast hits, scores, E-values, sizes, number of reads and conserved domains from putative proteins.

Table S2 EST-contigs with E-values < e^-20^ and scores > 100 obtained in the project Resistance, and their blast hits, scores, E-values, sizes, number of reads and conserved domains from putative proteins.

Table S3 EST-contigs with E-values < e^-20^ and scores > 100 obtained in the project Hypersensitive, and their blast hits, scores, E-values, sizes, number of reads and conserved domains from putative proteins.

Table S4 EST-contigs with E-values < e^-20^ and scores > 100 obtained in the project Pathogenesis, and their blast hits, scores, E-values, sizes, number of reads and conserved domains from putative proteins.

Table S5 EST-contigs with E-values < e^-20^ and scores > 100 obtained in the project Chitinase, and their blast hits, scores, E-values, sizes, number of reads and conserved domains from putative proteins

Table S6 EST-contigs with E-values < e^-20^ and scores > 100 obtained in the project Cytochrome P450, and their blast hits, scores, E-values, sizes, number of reads and conserved domains from putative proteins

Table S7 EST-contigs with E-values < e^-20^ and scores > 100 obtained in the project Glucanase, and their blast hits, scores, E-values, sizes, number of reads and conserved domains from putative proteins.

Table S8 EST-contigs with E-values < e^-20^ and scores > 100 obtained in the project HSP (Heat Shock Protein), and their blast hits, scores, E-values, sizes, number of reads and conserved domains from putative proteins.

Table S9 EST-contigs with E-values < e^-20^ and scores > 100 obtained in the project Thaumatin, and their blast hits, scores, E-values, sizes, number of reads and conserved domains from putative proteins.

Table S10 EST-contigs with E-values < e^-20^ and scores > 100 obtained in the project Phytoalexin, and their blast hits, scores, E-values, sizes, number of reads and conserved domains from putative proteins.

Table S11 EST-contigs with E-values < e^-20^ and scores > 100 obtained in the project Chalconesynthase, and their blast hits, scores, E-values, sizes, number of reads and conserved domains from putative proteins.

Table S12 EST-contigs with E-values < e^-20^ and scores > 100 obtained in the project Polyphenoloxydase, and their blast hits, scores, E-values, sizes, number of reads and conserved domains from putative proteins.

Table S13 EST-contigs with E-values < e^-20^ and scores > 100 obtained in the project Importin, and their blast hits, scores, E-values, sizes, number of reads and conserved domains from putative proteins

Table S14 EST-contigs with E-values < e^-20^ and scores > 100 obtained in the project Glucosyltransferase, and their blast hits, scores, E-values, sizes, number of reads and conserved domains from putative proteins.

Table S15 ESTs from the NS1, RM1, RX1 and SS1 libraries shown in Figure 1.

Figure S1 Distribution of the number of reads in the 140 EST-contigs with E-values < e^-20^ and scores > 100 formed after clustering.

Figure S2 Species with the most blast hits with the EST-contigs.

Figure S3 Average E-value distribution of the 20 best hits of the 140 EST-contigs submitted to BlastX by Blast2GO.

Figure S4 Average similarity value distribution of the 20 best hits of the 140 EST-contigs submitted to BlastX by Blast2GO.

Figure S5 Distribution of the number of GO terms (Cellular Component, Molecular Function and Biological Process) for the 140 EST-contigs analyzed by Blast2GO.

Figure S6 Evidence code (EC) distribution for the EST-contig blast hits.

This material is available as part of the online article from http://www.scielo.br/gmb.

## Figures and Tables

**Table 1:** The number of ESTs and their relative percentages obtained by keyword data mining in 14 keyword projects, the number of clusters (EST-contigs and singlets) formed and the number and percentage of EST-contigs with E-values < e^-20^ and scores > 100.

Project	ESTs	%	EST-contigs Total	Singlets	EST-contigs^1^	%
Chalconesynthase	153	1.35	5	8	5	0.51
Chitinase	1,855	16.41	47	48	45	4.59
CytochromeP450	2,441	21.60	235	202	144	14.70
Glucanase	642	5.68	92	68	88	8.98
Glucosyltransferase	1,286	11.38	130	160	125	12.76
HSP (Heat Shock Protein)	240	2.12	31	27	30	3.06
Hypersensitive	86	0.76	8	4	8	0.81
Importin	532	4.70	11	14	10	1.02
NBS-LRR	826	7.30	160	243	140	14.30
Pathogenesis	979	8.66	63	37	61	6.23
Phytoalexin	12	0.10	3	2	3	0.30
Polyphenoloxidase	67	0.59	4	3	4	0.40
Resistance	1,864	16.49	347	416	300	30.64
Thaumatin	317	2.80	16	7	16	1.63
Total	11,300	100	1,152	1,239	979	100

^1^E-value < e^-20^ and score > 100.

**Table 2:** Contigs differentially expressed between libraries containing the responses to biotic stress challenges and other libraries from the Brazilian Coffee Genome Project. Differential expression was confirmed by Fisher's exact test, with the p values indicated in the last column.

Cluster	# reads	Length	BlastX	Score	E-value	GenBank Record	p value
Contig 10650	15	1376	hypothetical protein [*Populus trichocarpa*]	99.4	8.00E-19	ref|XP_002319603.1|	0.00079
Contig 13908	30	1181	BURP domain-containing protein [*Solanum tuberosum*]	289	1.00E-76	gb|ACD49738.1|	0.00114
Contig 13986	9	794	cysteine protease inhibitor family protein / cystatin family protein [*Arabidopsis thaliana*]	46.2	0.003	ref|NP_193383.1|	0.0007
Contig 14592	131	1290	class III chitinase [*Coffea arabica*]	447	1.00E-123	emb|CAJ43737.1|	0.00263
Contig 431	33	821	cysteine protease inhibitor family protein / cystatin family protein [*Arabidopsis thaliana*]	45.8	0.005	ref|NP_193383.1|	0.00158
Contig 8478	65	2154	catalase [*Prunus avium*]	788	0.00	gb|ABM47415.1|	0.00193
Contig 9073	347	1819	catalase [*Nicotiana plumbaginifolia*]	915	0.00	emb|CAA85426.1|	0.00482
