# Supplementary material for: In silico identification of coffee genome expressed sequences potentially associated with resistance to diseases
Source: Genet Mol Biol. 2010 Dec 1;33(4):795–806. doi: 10.1590/s1415-47572010000400031 (PMC3036153; doi:10.1590/s1415-47572010000400031)
Supplement: Table S1 — EST-contigs with E-values < e-20 and scores > 100 obtained in the project NBS-LRR, and their blast hits, scores, E-values, sizes, number of reads and conserved domains from putative proteins. [file gmb-33-4-795-suppl1.pdf]

Table S1: EST-Contigs with e-value < e^{-20} and score > 100 obtained in the Project NBS-LRR, and their blast hit, score, e-value, size, number of reads, and conserved domains from putative proteins.

NBS-LRR						
Contig	BLAST NR	Score	e-value	Length	Reads	Conserved Domains
1	gi|46389753|dbj|BAD15107.1| hypothetical protein [Nicotiana tabacum]	168	1.00E-40	696	2	No CD has been identified
2	gi|55297132|dbj|BAD68775.1| putative NBS-LRR disease resistance protein [Oryza sativa (japonica cultivar-group)]	116	8.00E-25	801	2	pfam00931
3	gi|48209881|gb|AAT40487.1| putative disease resistance protein [Solanum demissum]	143	3.00E-33	623	2	pfam00931
4	gi|5734781|gb|AAD50046.1| Very similar to disease resistance proteins [Arabidopsis thaliana]	201	2.00E-50	985	2	pfam00931
5	gi|37781280|gb|AAP44392.1| nematode resistance-like protein [Solanum tuberosum]	222	1.00E-56	831	2	pfam01582, pfam00931, COG4886
6	gi|5231014|gb|AAD41050.1| NBS/LRR disease resistance protein RPM1 [Arabidopsis thaliana]	164	4.00E-39	1121	2	pfam00931, COG4886
7	gi|48057628|gb|AAT39951.1| putative disease resistance protein Prf [Solanum demissum]	132	9.00E-30	756	2	pfam00931
9	gi|15219278|ref|NP_175742.1| disease resistance protein (CC-NBS-LRR class), putative [Arabidopsis thaliana]	106	1.00E-21	953	2	pfam00931, pfam01576
10	gi|46389753|dbj|BAD15107.1| hypothetical protein [Nicotiana tabacum]	157	2.00E-37	617	2	No CD has been identified
11	gi|47900744|gb|AAT39316.1| putative resistance complex protein I2C-2 [Solanum demissum]	124	3.00E-27	762	2	pfam00931
12	gi|47824998|gb|AAT38770.1| putative late blight resistance protein [Solanum demissum]	125	1.00E-27	858	2	pfam00931
13	gi|50941633|ref|XP_480344.1| putative RGH1A [Oryza sativa (japonica cultivar-group)]	113	5.00E-24	782	2	pfam00931, COG4886
14	gi|21436417|gb|AAM51409.1| unknown protein [Arabidopsis thaliana]	359	6.00E-98	844	3	pfam08263, cd00116
15	gi|50428772|gb|AAT77096.1| Fom-2 protein [Cucumis melo]	166	1.00E-39	1033	3	No CD has been identified
16	gi|8547237|gb|AAF76312.1| Prf [Lycopersicon esculentum]	139	2.00E-49	1241	2	pfam00931
17	gi|61105091|gb|AAX38325.1| receptor-like protein kinase [Lycopersicon esculentum]	270	7.00E-71	1118	3	cd00116
18	gi|27311539|gb|AAO00735.1| expressed protein [Arabidopsis thaliana]	194	3.00E-48	874	2	cd00192
20	gi|62733933|gb|AAX96042.1| Leucine Rich Repeat, putative [Oryza sativa]	171	2.00E-41	711	2	cd00116
22	gi|13249030|gb|AAK16647.1| F-box containing protein TIR1 [Populus tremula x Populus tremuloides]	431	0	1065	4	cd00116
24	gi|50428772|gb|AAT77096.1| Fom-2 protein [Cucumis melo]	252	2.00E-65	1852	3	No CD has been identified
25	gi|18181933|dbj|BAB83871.1| disease resistance protein [Arabidopsis thaliana]	172	2.00E-45	867	4	pfam00931
26	gi|52075880|dbj|BAD45826.1| putative CMP-sialic acid transporter [Oryza sativa]	179	1.00E-88	654	2	pfam04142
27	gi|9758146|dbj|BAB08703.1| disease resistance protein [Arabidopsis thaliana]	110	7.00E-23	912	4	pfam00931, COG4886
28	gi|50939001|ref|XP_479028.1| putative disease resistance protein RPH8 [Oryza sativa (japonica cultivar-group)]	227	2.00E-77	1443	3	pfam00931, COG4886
29	gi|48057658|gb|AAT39957.1| putative late blight resistance protein [Solanum demissum]	206	2.00E-79	1266	4	pfam00931
30	gi|21554189|gb|AAM63268.1| putative leucine-rich repeat disease resistance protein [Arabidopsis thaliana]	432	0	1335	7	cd00116
31	gi|46389753|dbj|BAD15107.1| hypothetical protein [Nicotiana tabacum]	168	1.00E-40	803	3	No CD has been identified
32	gi|37718761|gb|AAR01633.1| putative leucine-rich repeat receptor kinase [Oryza sativa]	312	0	1833	4	cd00192, smart00220
33	gi|6456755|gb|AAF09256.1| disease resistance protein BS2 [Capsicum chacoense]	358	4.00E-97	2540	15	pfam00931
35	gi|50939001|ref|XP_479028.1| putative disease resistance protein RPH8 [Oryza sativa (japonica cultivar-group)]	325	4.00E-87	1857	7	pfam00931, COG4886
36	gi|21805750|gb|AAM76770.1| hypothetical protein [Arabidopsis thaliana]	176	9.00E-43	965	5	pfam07320
37	gi|29839503|sp|P59584|RP8HA_ARATH Disease resistance protein RPH8A (RPP8 homolog A) [Arabidopsis thaliana]	293	2.00E-77	2164	5	pfam00931
38	gi|15240263|ref|NP_200956.1| leucine-rich repeat transmembrane protein kinase, putative [Arabidopsis thaliana]	523	0	1416	3	cd00180, cd00116, smart00220, COG4886
39	gi|7270910|emb|CAB80590.1| receptor protein kinase-like protein [Arabidopsis thaliana]	184	5.00E-45	1239	2	cd00192, cd00116, smart00220
40	gi|39636723|gb|AAR29070.1| blight resistance protein RGA1 [Solanum bulbocastanum]	248	4.00E-64	1540	2	pfam00931, COG4886
41	gi|46389753|dbj|BAD15107.1| hypothetical protein [Nicotiana tabacum]	145	2.00E-33	785	2	No CD has been identified
42	gi|26450926|dbj|BAC42570.1| putative receptor protein kinase [Arabidopsis thaliana]	286	9.00E-76	1307	3	pfam08263, cd00192, smart00220, COG4886
44	gi|50939001|ref|XP_479028.1| putative disease resistance protein RPH8 [Oryza sativa (japonica cultivar-group)]	291	4.00E-77	1981	6	pfam00931, COG4886
45	gi|50945887|ref|XP_482471.1| putative disease resistance gene homolog [Oryza sativa (japonica cultivar-group)]	137	2.00E-34	1313	4	pfam00931, COG4886
46	gi|4835246|emb|CAB42924.1| putative disease resistance protein [Arabidopsis thaliana]	216	1.00E-54	1500	2	pfam00931, PRK11281
47	gi|24459865|emb|CAC82608.1| disease resistance-like protein [Coffea canephora]	157	6.00E-37	1025	3	pfam00931
48	gi|30689664|ref|NP_195056.2| disease resistance protein (CC-NBS-LRR class), putative [Arabidopsis thaliana]	652	0	2255	13	pfam00931
49	gi|47059739|gb|AAT09451.1| putative NBS-LRR type disease resistance protein [Prunus persica]	461	0	3281	15	pfam00931, COG4886
50	gi|49333393|gb|AAT64032.1| putative leucine-rich repeat transmembrane protein; putative protein kinase [Gossypium hirsutum]	227	9.00E-59	469	3	pfam08263, cd00192, smart00220, COG4886
51	gi|9651941|gb|AAF91322.1| receptor-like protein kinase 1 [Glycine max]	531	0	831	3	cd00180, cd00116, smart00220, pfam08263
52	gi|20466151|gb|AAM20393.1| transport inhibitor response 1, putative [Arabidopsis thaliana]	222	7.00E-57	641	2	No CD has been identified
53	gi|39636816|gb|AAR29076.1| blight resistance protein T118 [Solanum tarijense]	126	5.00E-30	768	4	pfam00931, COG4886
54	gi|50945887|ref|XP_482471.1| putative disease resistance gene homolog [Oryza sativa (japonica cultivar-group)]	246	1.00E-63	1619	5	pfam00931, COG4886
55	gi|7573427|emb|CAB87743.1| transport inhibitor response 1 (TIR1) [Arabidopsis thaliana]	280	3.00E-74	812	5	No CD has been identified
56	gi|14573459|gb|AAK68074.1| somatic embryogenesis receptor-like kinase 3 [Arabidopsis thaliana]	210	2.00E-53	1118	3	cd00180, COG4886, smart00220, pfam08263
57	gi|9279736|dbj|BAB01326.1| receptor-like kinase [Arabidopsis thaliana]	382	0	816	2	cd00192, cd00116, pfam08263
58	gi|8547237|gb|AAF76312.1| Prf [Lycopersicon esculentum]	194	6.00E-48	1246	2	pfam00931
59	gi|25403295|pir||B86398 protein T7N9.24 [imported] - [Arabidopsis thaliana]	132	3.00E-60	1059	2	smart00255, pfam00931, COG4886, cd00116
60	gi|34909106|ref|NP_915900.1| putative NBS-LRR type resistance protein [Oryza sativa]	100	3.00E-25	900	2	pfam00931, COG4886
61	gi|17979045|gb|AAL49790.1| unknown protein [Arabidopsis thaliana]	291	1.00E-77	859	2	cd00180, cd00116, smart00220, pfam08263

63	gi|44717048|gb|AAN62015.2| leucine-rich repeat protein [Capsicum annum]	262	9.00E-69	821	4	pfam08263
66	gi|38566726|emb|CAE76632.1| leucine rich repeat protein [Cicer arietinum]	500	0	1315	3	cd00116, pfam08263
67	gi|46389753|dbj|BAD15107.1| hypothetical protein [Nicotiana tabacum]	194	1.00E-56	1239	3	No CD has been identified
68	gi|3360289|gb|AAC27894.1| leucine-rich repeat transmembrane protein kinase 1 [Zea mays]	407	0	1182	3	cd00180, cd00116, smart00220, pfam08263
69	gi|46095229|gb|AAS80152.1| FOM-2 [Cucumis melo]	177	3.00E-43	1022	3	No CD has been identified
70	gi|40218005|gb|AAR82926.1| coronatine-insensitive 1 [Lycopersicon esculentum]	469	0	1442	3	No CD has been identified
71	gi|38564276|gb|AAR23717.1| At4g22730 [Arabidopsis thaliana]	343	6.00E-93	1078	3	cd00192, pfam08263, smart00220, COG4886
72	gi|19699206|gb|AAL90969.1| At1g21410/F24J8_17 [Arabidopsis thaliana]	502	0	1333	3	smart00367, cd00116
73	gi|8515762|gb|AAF76163.1| RGC1 [Solanum tuberosum]	290	6.00E-77	1239	5	pfam00931
74	gi|48057628|gb|AAT39951.1| putative disease resistance protein Prf [Solanum demissum]	149	7.00E-35	691	2	pfam00931
75	gi|27311539|gb|AAO00735.1| expressed protein [Arabidopsis thaliana]	468	0	1235	5	cd00192
76	gi|8547232|gb|AAF76308.1| Prf [Lycopersicon pimpinellifolium]	196	9.00E-62	744	2	pfam00931
77	gi|50428772|gb|AAT77096.1| Fom-2 protein [Cucumis melo]	166	1.00E-39	1384	2	No CD has been identified
78	gi|20259553|gb|AAM14119.1| putative receptor protein kinase [Arabidopsis thaliana]	429	0	895	2	cd00180, COG4886, smart00220, cd00116
79	gi|37718761|gb|AAR01633.1| putative leucine-rich repeat receptor kinase [Oryza sativa]	217	3.00E-60	955	2	cd00192, smart00220
80	gi|28393326|gb|AAO42089.1| putative receptor protein kinase [Arabidopsis thaliana]	297	7.00E-90	1021	5	cd00180,cd00116, smart00220, pfam08263
81	gi|42566272|ref|NP_192248.2| leucine-rich repeat transmembrane protein, putative [Arabidopsis thaliana]	194	9.00E-49	518	2	cd00192, smart00220, COG4886
82	gi|5541686|emb|CAB51192.1| putative protein [Arabidopsis thaliana]	254	6.00E-84	2331	8	pfam00931
83	gi|50939001|ref|XP_479028.1| putative disease resistance protein RPH8 [Oryza sativa (japonica cultivar-group)]	390	0	2376	4	pfam00931, COG4886
84	gi|42566272|ref|NP_192248.2| leucine-rich repeat transmembrane protein RPH8A [Oryza sativa]	167	2.00E-40	702	2	cd00192, smart00220, COG4886
86	gi|48057628|gb|AAT39951.1| putative disease resistance protein Prf [Solanum demissum]	357	7.00E-97	1571	7	pfam00931
87	gi|5231014|gb|AAD41050.1| NBS/LRR disease resistance protein RPM1 [Arabidopsis thaliana]	114	3.00E-24	694	2	pfam00931, COG4886
88	gi|50428772|gb|AAT77096.1| Fom-2 protein [Cucumis melo]	105	1.00E-21	672	2	No CD has been identified
89	gi|21239384|gb|AAM44275.1| receptor-like kinase RHG4 [Glycine max]	375	0	1395	2	cd00180, smart00220, cd00116
90	gi|48209881|gb|AAT40487.1| putative disease resistance protein [Solanum demissum]	128	1.00E-28	559	2	pfam00931
91	gi|42572433|ref|NP_974312.1| leucine-rich repeat transmembrane protein kinase, putative [Arabidopsis thaliana]	257	5.00E-67	1325	5	cd00192, smart00220, COG4886
92	gi|15218941|ref|NP_176789.1| leucine-rich repeat protein kinase, putative (TMK1) [Arabidopsis thaliana]	556	0	1049	4	cd00180, cd00116, smart00220, COG4886
93	gi|48209881|gb|AAT40487.1| putative disease resistance protein [Solanum demissum]	124	4.00E-27	802	2	pfam00931
94	gi|50945887|ref|XP_482471.1| putative disease resistance gene homolog [Oryza sativa (japonica cultivar-group)]	207	8.00E-52	1693	3	pfam00931, COG4886
95	gi|24459841|emb|CAC82597.1| disease resistance-like protein [Coffea arabica]	260	2.00E-68	778	2	pfam00931
96	gi|32364526|gb|AAP80292.1| resistance protein Tsu5 [Arabidopsis thaliana]	105	1.00E-21	931	2	pfam00931
97	gi|24459845|emb|CAC82610.1| disease resistance-like protein [Coffea arabica]	161	2.00E-58	1047	2	pfam00931
99	gi|7672732|gb|AAF66615.1| LRR receptor-like protein kinase [Nicotiana tabacum]	364	2.00E-99	766	5	cd00180, smart00220
100	gi|8547237|gb|AAF76312.1| Prf [Lycopersicon esculentum]	208	2.00E-52	953	3	pfam00931
101	gi|4689223|gb|AAD27815.1| disease resistance protein I2 [Lycopersicon esculentum]	130	4.00E-29	715	2	pfam00931
102	gi|55771369|dbj|BAD72536.1| LIM domain containing protein-like [Oryza sativa]	438	0	885	3	smart00132
103	gi|21689699|gb|AAM67471.1| putative F-box family protein AtFBL3 [Arabidopsis thaliana]	321	2.00E-86	783	5	No CD has been identified
104	gi|47059739|gb|AAT09451.1| putative NBS-LRR type disease resistance protein [Prunus persica]	200	7.00E-50	1107	4	pfam00931, COG4886
105	gi|42569070|ref|NP_179220.2| leucine-rich repeat transmembrane protein kinase, putative [Arabidopsis thaliana]	183	7.00E-45	1102	3	cd00192, smart00220, COG4886
106	gi|48209881|gb|AAT40487.1| putative disease resistance protein [Solanum demissum]	148	2.00E-34	729	2	pfam00931
107	gi|5734781|gb|AAD50046.1| Very similar to disease resistance proteins [Arabidopsis thaliana]	142	5.00E-33	655	2	pfam00931
108	gi|16323486|gb|AAL15237.1| putative protein translocation complex Sec61 gamma chain [Arabidopsis thaliana]	102	5.00E-21	548	2	COG2443
109	gi|24459853|emb|CAC82602.1| disease resistance-like protein [Coffea arabica]	300	7.00E-80	1131	5	pfam00931
113	gi|30698151|ref|NP_201372.2| leucine-rich repeat transmembrane protein kinase, putative [Arabidopsis thaliana]	232	3.00E-62	860	3	cd00192, smart00220, COG4886
114	gi|46095229|gb|AAS80152.1| FOM-2 [Cucumis melo]	104	8.00E-21	1558	3	No CD has been identified
115	gi|11994124|dbj|BAB01126.1| receptor protein kinase [Arabidopsis thaliana]	274	3.00E-72	848	2	cd00192, cd00116, smart00220, pfam08263
116	gi|46389753|dbj|BAD15107.1| hypothetical protein [Nicotiana tabacum]	216	1.00E-54	1229	4	No CD has been identified
117	gi|50899184|ref|XP_450380.1| putative disease related protein 2 [Oryza sativa]	138	1.00E-65	1886	4	pfam00931, COG4886
119	gi|13249030|gb|AAK16647.1| F-box containing protein TIR1 [Populus tremula x Populus tremuloides]	200	2.00E-70	771	3	cd00116
120	gi|62733050|gb|AAX95167.1| receptor-like protein kinase [Oryza sativa]	329	5.00E-89	735	3	cd00192, smart00220, COG4886
121	gi|53828509|gb|AAU94364.1| At1g27190 [Arabidopsis thaliana]	256	5.00E-72	712	2	cd00192, cd05086
122	gi|46389753|dbj|BAD15107.1| hypothetical protein [Nicotiana tabacum]	139	1.00E-31	934	3	No CD has been identified
123	gi|50945889|ref|XP_482472.1| putative disease resistance gene homolog [Oryza sativa (japonica cultivar-group)]	145	9.00E-34	779	4	pfam00931, COG4886
124	gi|9758140|dbj|BAB08632.1| disease resistance protein-like [Arabidopsis thaliana]	169	1.00E-40	936	2	pfam00931, COG4886
125	gi|5478530|gb|AAD43920.1| UVB-resistance protein UVR8 [Arabidopsis thaliana]	453	0	802	4	pfam00415, COG5184
126	gi|48057658|gb|AAT39957.1| putative late blight resistance protein [Solanum demissum]	164	2.00E-39	786	2	pfam00931
127	gi|30385250|gb|AAP23944.1| leucine-rich repeat protein [x Citrofortunella mitis]	360	7.00E-98	1380	20	cd00116, pfam08263
128	gi|8547237|gb|AAF76312.1| Prf [Lycopersicon esculentum]	105	3.00E-21	1325	3	pfam00931
129	gi|48057628|gb|AAT39951.1| putative disease resistance protein Prf [Solanum demissum]	205	2.00E-51	1058	2	pfam00931
131	gi|34913790|ref|NP_918242.1| putative LRR [Oryza sativa (japonica cultivar-group)]	143	1.00E-34	799	2	pfam00931, COG4886
132	gi|50905715|ref|XP_464346.1| putative protein kinase 2 [Oryza sativa]	184	6.00E-48	824	2	cd00180, smart00220
133	gi|50918423|ref|XP_469608.1| hypothetical protein [Oryza sativa (japonica cultivar-group)]	334	2.00E-90	1019	5	cd02970
134	gi|50925601|ref|XP_472985.1| OSJNBa0084K20.12 [Oryza sativa (japonica cultivar-group)]	236	6.00E-61	730	2	pfam04142

135	gi|55297132|dbj|BAD68775.1| putative NBS-LRR disease resistance protein [Oryza sativa (japonica cultivar-group)]	124	3.00E-27	814	3	pfam00931
136	gi|53689783|gb|AAU89792.1| late blight resistance protein-like [Solanum tuberosum]	152	8.00E-36	803	2	pfam00931, smart00534
137	gi|24459843|emb|CAC82598.1| disease resistance-like protein [Coffea arabica]	138	4.00E-31	1237	3	pfam00931
138	gi|39636816|gb|AAR29076.1| blight resistance protein T118 [Solanum tarijense]	172	2.00E-41	1124	2	pfam00931, COG4886
139	gi|8547237|gb|AAF76312.1| Prf [Lycopersicon esculentum]	332	1.00E-89	1366	2	pfam00931
140	gi|62734129|gb|AAX96238.1| NB-ARC domain, putative [Oryza sativa (japonica cultivar-group)]	111	4.00E-23	1259	3	pfam00931
141	gi|15222893|ref|NP_175437.1| disease resistance protein (CC-NBS-LRR class), putative [Arabidopsis thaliana]	108	1.00E-22	711	2	pfam00931
142	gi|38603954|gb|AAR24722.1| At5g49400 [Arabidopsis thaliana]	197	3.00E-49	709	2	No CD has been identified
144	gi|7270269|emb|CAB80038.1| putative protein [Arabidopsis thaliana]	470	0	990	2	smart00256
145	gi|15222893|ref|NP_175437.1| disease resistance protein (CC-NBS-LRR class), putative [Arabidopsis thaliana]	338	3.00E-91	1906	5	pfam00931
146	gi|50941263|ref|XP_480159.1| putative U3 snoRNP protein IMP4 [Oryza sativa]	388	0	744	4	pfam04427
147	gi|6456755|gb|AAF09256.1| disease resistance protein BS2 [Capsicum chacoense]	216	6.00E-98	2019	6	pfam00931
148	gi|34896972|ref|NP_909832.1| putative leucine-rich repeat protein [Oryza sativa]	303	6.00E-81	1102	36	cd00116, pfam08263
149	gi|15223744|ref|NP_172891.1| leucine-rich repeat transmembrane protein kinase, putative [Arabidopsis thaliana]	339	8.00E-92	1150	2	cd00192, smart00220, COG4886
150	gi|22652532|gb|AAN03742.1| NBS-LRR-like protein [Oryza sativa (japonica cultivar-group)]	222	4.00E-56	2251	6	pfam00931
151	gi|20259113|gb|AAM14272.1| unknown protein [Arabidopsis thaliana]	643	0	1958	18	No CD has been identified
152	gi|5080812|gb|AAD39321.1| Putative disease resistance protein [Arabidopsis thaliana]	181	5.00E-44	1580	3	pfam00931
154	gi|62321425|dbj|BAD94804.1| putative protein [Arabidopsis thaliana]	170	4.00E-41	870	3	No CD has been identified
155	gi|48057628|gb|AAT39951.1| putative disease resistance protein Prf [Solanum demissum]	227	5.00E-58	1120	2	pfam00931
156	gi|50945887|ref|XP_482471.1| putative disease resistance gene homolog [Oryza sativa (japonica cultivar-group)]	145	1.00E-51	1558	4	pfam00931, COG4886
157	gi|48057628|gb|AAT39951.1| putative disease resistance protein Prf [Solanum demissum]	241	7.00E-62	1745	6	pfam00931
159	gi|48209881|gb|AAT40487.1| putative disease resistance protein [Solanum demissum]	146	5.00E-34	639	2	pfam00931
