# Supplementary material for: In silico identification of coffee genome expressed sequences potentially associated with resistance to diseases
Source: Genet Mol Biol. 2010 Dec 1;33(4):795–806. doi: 10.1590/s1415-47572010000400031 (PMC3036153; doi:10.1590/s1415-47572010000400031)
Supplement: Table S2 — EST-contigs with E-values < e-20 and scores > 100 obtained in the project Resistance, and their blast hits, scores, E-values, sizes, number of reads and conserved domains from putative proteins. [file gmb-33-4-795-suppl2.pdf]

Table S2: EST-Contigs with e-value < e^{-20} and score > 100 obtained in the Project Resistance, and their blast hit, score, e-value, size, number of reads, and conserved domains from putative proteins.

Resistance						
Contig	BLAST NR	Score	e-value	Length	Reads	Conserved Domains
2	gi|118489526|gb|ABK96565.1|[118489526] unknown [Populus trichocarpa x Populus deltoides]	159	7.00E-38	454	2	No CD has been identified
4	gi|46389753|dbj|BAD15107.1| hypothetical protein [Nicotiana tabacum]	168	1.00E-40	696	2	No CD has been identified
5	gi|55297132|dbj|BAD68775.1| putative NBS-LRR disease resistance protein [Oryza sativa (japonica cultivar-group)]	116	1.00E-24	839	3	pfam00931
6	gi|82581291|dbj|BAE48710.1| NRAMP family metal transporter [Chengiopanax sciadophylloides]	404	0	1922	6	PRK00701
7	gi|82581291|dbj|BAE48710.1| NRAMP family metal transporter [Chengiopanax sciadophylloides]	480	0	1170	14	PRK00701
8	gi|15228832|ref|NP_191829.1| ATMRP10 [Arabidopsis thaliana]	343	3.00E-93	848	4	cd03244, cd03250, pfam00664, cd03271, cd03233, COG1132
9	gi|15220789|ref|NP_175748.1| kinase [Arabidopsis thaliana]	635	0	1342	3	cd00192, COG4886
11	gi|48209881|gb|AAT40487.1| putative disease resistance protein [Solanum demissum]	143	4.00E-33	623	2	pfam00931
12	gi|40846374|gb|AAR92477.1| putative WRKY transcription factor 30 [Vitis aestivalis]	198	1.00E-49	767	2	pfam03106
13	gi|157358243|emb|CAO65880.1|[157358243] unnamed protein product [Vitis vinifera]	502	0	1242	5	No CD has been identified
14	gi|5734781|gb|AAD50046.1| Very similar to disease resistance proteins [Arabidopsis thaliana]	201	3.00E-50	985	2	pfam00931
15	gi|79316282|ref|NP_001030932.1| ATP binding [Arabidopsis thaliana]	293	5.00E-78	809	6	cd00550, pfam02374
16	gi|15220948|ref|NP_172851.1| GN (GNOM) [Arabidopsis thaliana]	309	0	1306	5	cd00171, COG5307
17	gi|41052472|dbj|BAD07483.1| PDR-type ABC transporter 1 [Nicotiana tabacum]	169	4.00E-65	1098	5	cd03232, cd03233, cd03234, pfam01061, pfam08370, cd03226, pfam00005
18	gi|67933183|ref|ZP_00526308.1| regulatory protein, ArsR [Solibacter usitatus Ellin6076]	125	2.00E-27	682	2	cd00090
19	gi|77382823|gb|ABA74336.1| Peptidase C39, bacteriocin processing [Pseudomonas fluorescens PfO-1]	277	3.00E-73	827	18	cd02423
20	gi|34104782|gb|AAQ61139.1| probable manganese transport transmembrane protein [Chromobacterium violaceum ATCC 12472]	346	5.00E-94	798	7	COG1914
21	gi|37781280|gb|AAP44392.1| nematode resistance-like protein [Solanum tuberosum]	222	1.00E-56	831	2	pfam01582, pfam00931, COG4886
22	gi|5231014|gb|AAD41050.1| NBS/LRR disease resistance protein RPM1 [Arabidopsis thaliana]	164	6.00E-39	1121	2	pfam00931, COG4886
23	gi|50928783|ref|XP_473919.1| OSJNBa0058K23.17 [Oryza sativa (japonica cultivar-group)]	395	0	1327	10	COG1132, pfam00664, cd03250, cd03244, cd03231
24	gi|14715462|dbj|BAB62040.1| CjMDR1 [Coptis japonica]	265	1.00E-69	769	2	cd03249, pfam00664, COG1132
25	gi|34898274|ref|NP_910483.1| similar to NBS-LRR type resistance gene [Oryza sativa (japonica cultivar-group)]	84	9.00E-24	946	2	pfam00931, COG4886
26	gi|15237456|ref|NP_199466.1| ATPase, coupled to transmembrane movement of substances [Arabidopsis thaliana]	432	0	994	3	cd03249, pfam00664, COG1132, cd03270
29	gi|72117527|gb|AAZ59790.1| ABC transporter related [Ralstonia eutropha JMP134]	157	1.00E-37	283	2	cd03230, COG1131
30	gi|15217776|ref|NP_174115.1| ATPase, coupled to transmembrane movement of substances [Arabidopsis thaliana]	361	0	1034	2	cd03249, pfam00664, COG1132, cd03270
31	gi|15230349|ref|NP_188563.1| protein binding [Arabidopsis thaliana]	236	8.00E-61	740	2	cd00116
32	gi|42563438|ref|NP_186900.3| Ran GTPase binding / chromatin binding [Arabidopsis thaliana]	350	0	1542	4	pfam00415, COG5184
33	gi|9623337|gb|AAF90123.1| Rar1 [Hordeum vulgare]	162	1.00E-38	761	5	pfam04968
35	gi|68557365|ref|ZP_00596706.1| Drug resistance transporter EmrB/QacA subfamily [Ralstonia metallidurans CH34]	279	7.00E-75	777	6	PRK10504, pfam07690
36	gi|16418843|gb|AAL19297.1| putative diguanylate cyclase/phosphodiesterase domain 1 [Salmonella enterica subsp. enterica serovar Choleraesuis str. SC-B67]	185	1.00E-45	741	4	cd01948, COG4943
37	gi|30678939|ref|NP_187029.2| ankyrin repeat family protein / regulator of chromosome condensation (RCC1) family protein [Arabidopsis thaliana]	328	3.00E-88	1369	6	cd00204, pfam00415, COG5184
38	gi|15219278|ref|NP_175742.1| disease resistance protein (CC-NBS-LRR class), putative [Arabidopsis thaliana]	106	1.00E-21	953	2	pfam00931, pfam01576
39	gi|46389753|dbj|BAD15107.1| hypothetical protein [Nicotiana tabacum]	157	3.00E-37	617	2	No CD has been identified
40	gi|50904597|ref|XP_463787.1| putative senescence-associated protein [Oryza sativa (japonica cultivar-group)]	138	2.00E-31	737	6	cd00158
41	gi|47900744|gb|AAT39316.1| putative resistance complex protein I2C-2, putative [Solanum demissum]	124	3.00E-27	762	2	pfam00931
43	gi|15235780|ref|NP_194004.1| leucine-rich repeat transmembrane protein kinase, putative [Arabidopsis thaliana]	264	3.00E-69	762	3	cd00192, COG4886, pfam08263, smart00220
44	gi|13377502|gb|AAK20740.1| LRK33 [Triticum aestivum]	269	1.00E-70	897	2	cd00192, smart00220
45	gi|77554673|gb|ABA97469.1| expressed protein [Oryza sativa (japonica cultivar-group)]	355	1.00E-96	701	2	pfam00415, COG5184
46	gi|47824998|gb|AAT38770.1| putative late blight resistance protein [Solanum demissum]	125	2.00E-27	858	2	pfam00931
47	gi|68213281|ref|ZP_00565114.1| ABC-2 [Methylobacillus flagellatus KT]	204	2.00E-51	662	3	COG0842
48	gi|77383604|gb|ABA75117.1| Two component Heavy metal response transcriptional regulator, winged helix family [Pseudomonas fluorescens PfO-1]	343	3.00E-93	817	11	cd00156, cd00383, PRK09836
49	gi|50941633|ref|XP_480344.1| putative RGH1A [Oryza sativa (japonica cultivar-group)]	113	6.00E-24	782	2	pfam00931
50	gi|8547237|gb|AAF76312.1| Prf [Lycopersicon esculentum]	121	5.00E-26	1079	4	pfam00931
51	gi|15232373|ref|NP_188718.1| leucine-rich repeat family protein [Arabidopsis thaliana]	359	8.00E-98	844	3	pfam08263, cd00116
52	gi|50428772|gb|AAT77096.1| Fom-2 protein [Cucumis melo]	166	1.00E-39	1033	3	No CD has been identified
53	gi|8547237|gb|AAF76312.1| Prf [Lycopersicon esculentum]	139	2.00E-49	1241	2	pfam00931
54	gi|21326114|gb|AAM47580.1| putative ABC-transporter-like protein [Sorghum bicolor]	333	2.00E-91	806	2	cd03249, pfam00664, COG1132
56	gi|18394385|ref|NP_564003.1| ATP binding / kinase/ protein kinase/ protein [Arabidopsis thaliana]	368	0	1222	5	cd00192
58	gi|55056944|emb|CAH39854.1| PDR-like ABC transporter [Nicotiana tabacum]	331	3.00E-99	774	3	pfam01061

59	gi|62733933|gb|AAX96042.1| Leucine Rich Repeat, putative [Oryza sativa]	171	2.00E-41	711	2	cd00116
60	gi|79318957|ref|NP_001031116.1| ATMRP1 (Arabidopsis thaliana multidrug resistance-associated protein 1); xenobiotic-transporting ATPase. [Arabidopsis thaliana]	100	6.00E-20	783	2	cd03244, cd03250, pfam00664, cd03297, COG1132
62	gi|15227133|ref|NP_182301.1| ATMRP4 [Arabidopsis thaliana]	429	0	1079	5	cd03244, cd03250, pfam00664, cd03271, cd03233, COG1132
65	gi|77383868|gb|ABA75381.1| Secretion protein HlyD [Pseudomonas fluorescens PfO-1]	425	0	744	6	PRK10476, pfam00529
66	gi|72117689|gb|AAZ59952.1| Acriflavin resistance protein [Ralstonia eutropha JMP134]	128	9.00E-29	496	2	pfam02355, PRK10614
67	gi|54287494|gb|AAV31238.1| putative 26S proteasome non-ATPase regulatory subunit 14 [Oryza sativa (japonica cultivar-group)]	536	0	1247	12	smart00232
68	gi|50428772|gb|AAT77096.1| Fom-2 protein [Cucumis melo]	252	3.00E-65	1852	3	No CD has been identified
69	gi|15217954|ref|NP_176135.1| disease resistance protein (CC-NBS-LRR class), putative [Arabidopsis thaliana]	172	2.00E-45	867	4	pfam00931
70	gi|15238507|ref|NP_198395.1| disease resistance protein (CC-NBS-LRR class), putative [Arabidopsis thaliana]	110	9.00E-23	912	4	pfam00931, COG4886
71	gi|50939001|ref|XP_479028.1| putative disease resistance protein RPH8A [Oryza sativa (japonica cultivar-group)]	227	2.00E-77	1443	3	pfam00931, COG4886
72	gi|15220948|ref|NP_172851.1| GN (GNOM) [Arabidopsis thaliana]	416	0	753	5	cd00171, COG5307
73	gi|84453202|dbj|BAE71198.1| putative transporter-like protein [Trifolium pratense]	186	8.00E-46	799	6	PRK09874, COG2814, pfam07690
74	gi|21554189|gb|AAM63268.1| putative leucine-rich repeat disease resistance protein [Arabidopsis thaliana]	432	0	1386	7	cd00116
75	gi|48093465|gb|AAT40109.1| putative UDP-glucuronate decarboxylase 3 [Nicotiana tabacum]	704	0	2073	18	PRK10084, COG0451
76	gi|46389753|dbj|BAD15107.1| hypothetical protein [Nicotiana tabacum]	168	2.00E-40	803	3	No CD has been identified
77	gi|48788902|ref|ZP_00284881.1| COG0654: 2-polyprenyl-6-methoxyphenol hydroxylase and related FAD-dependent oxidoreductases [Burkholderia fungorum LB400]	228	1.00E-58	788	12	COG0654, PRK06185
78	gi|37534400|ref|NP_921502.1| hypothetical protein [Oryza sativa (japonica cultivar-group)]	172	2.00E-57	784	2	pfam03732, pfam08284, pfam00665, pfam00385
79	gi|6456755|gb|AAF09256.1| disease resistance protein BS2 [Capsicum chacoense]	358	6.00E-97	2533	15	pfam00931
81	gi|15227133|ref|NP_182301.1| ATMRP4 [Arabidopsis thaliana]	595	0	1353	3	cd03244, cd03250, pfam00664, cd03271, cd03233, COG1132
82	gi|50939001|ref|XP_479028.1| putative disease resistance protein RPH8A [Oryza sativa (japonica cultivar-group)]	325	5.00E-87	1857	7	pfam00931, COG4886
83	gi|15225931|ref|NP_182138.1| unknown protein [Arabidopsis thaliana]	176	1.00E-42	965	5	pfam07320
84	gi|29839503|sp|P59584|RP8HA_ARATH Disease resistance protein RPH8A (RPP8 homolog A) [Arabidopsis thaliana]	293	2.00E-77	2164	5	pfam00931
85	gi|79594244|ref|NP_850245.2| hydrolase [Arabidopsis thaliana]	298	1.00E-79	902	2	cd01311
86	gi|68344526|gb|AAY92132.1| multidrug RND efflux transporter, permease protein MdtB [Pseudomonas fluorescens Pf-5]	292	3.00E-78	510	11	pfam02355, PRK10503
87	gi|39636723|gb|AAR29070.1| blight resistance protein RGA1 [Solanum bulbocastanum]	248	5.00E-64	1540	2	pfam00931, COG4886
88	gi|55773917|dbj|BAD72522.1| putative multidrug resistance-associated protein 7 [Oryza sativa (japonica cultivar-group)]	142	6.00E-33	537	2	cd03244, cd03250, pfam00664, cd03215, COG1132
89	gi|5824321|emb|CAB54139.1| ATPase [Solanum tuberosum]	372	0	1121	4	cd0055
90	gi|5478530|gb|AAD43920.1| UVB-resistance protein UVR8 [Arabidopsis thaliana]	523	0	1308	8	pfam00415, COG5184
92	gi|68342685|gb|AAY90291.1| fusaric acid resistance protein, putative [Pseudomonas fluorescens Pf-5]	478	0	798	9	pfam04632, COG4129, COG1289
93	gi|46389753|dbj|BAD15107.1| hypothetical protein [Nicotiana tabacum]	145	2.00E-33	785	2	No CD has been identified
94	gi|15220442|ref|NP_172010.1|AXR1 (AUXIN RESISTANT 1); small protein activating enzyme [Arabidopsis thaliana]	311	2.00E-83	831	2	cd01493
97	gi|15240747|ref|NP_196345.1| EMS1 (EXCESS MICROSPOROCYTES1); kinase [Arabidopsis thaliana]	164	4.00E-39	805	3	cd00192, COG4886, cd00116, smart00220
98	gi|50939001|ref|XP_479028.1| putative disease resistance protein RPH8A [Oryza sativa (japonica cultivar-group)]	291	5.00E-77	1981	6	pfam00931, COG4886
99	gi|18416049|ref|NP_567674.1| unknown protein [Arabidopsis thaliana]	652	0	1469	6	pfam03105, pfam07690
100	gi|15227133|ref|NP_182301.1| ATMRP4 [Arabidopsis thaliana]	978	0	2599	10	cd03244, cd03250, pfam00664, cd03271, cd03233, COG1132
101	gi|15228004|ref|NP_181808.1| protein binding [Arabidopsis thaliana]	119	1.00E-25	794	2	COG0842
102	gi|56381949|gb|AAV85693.1| At5g59250 [Arabidopsis thaliana]	244	4.00E-63	881	2	pfam00083
103	gi|51971421|dbj|BAD44375.1| unnamed protein product [Arabidopsis thaliana]	390	0	875	3	cd03249, pfam00664
104	gi|50945887|ref|XP_482471.1| putative disease resistance gene homolog [Oryza sativa (japonica cultivar-group)]	137	3.00E-34	1313	4	pfam00931, COG4886
105	gi|14626935|gb|AAK70805.1| leucine-rich repeat resistance protein-like protein [Gossypium hirsutum]	332	2.00E-93	873	4	COG4886
107	gi|38327504|gb|AAR17783.1| ribosomal protein L3 [Lycopersicon esculentum]	457	0	819	2	PRK04231
108	gi|15230357|ref|NP_190664.1| ATP binding [Arabidopsis thaliana]	216	1.00E-54	1500	2	pfam00931, PRK11281
109	gi|15231620|ref|NP_191462.1| TT12 (TRANSPARENT TESTA 12); antiporter/transporter [Arabidopsis thaliana]	338	1.00E-91	872	9	pfam01554, COG0534
110	gi|74040324|gb|AAZ95005.1| late blight resistance protein Rpi-blb2 [Solanum bulbocastanum]	157	6.00E-37	1023	4	pfam00931
111	gi|77556936|gb|ABA99732.1| disease resistance, putative [Oryza sativa]	671	0	2255	13	pfam00931
112	gi|18407955|ref|NP_566879.1| MLO3; calmodulin binding [Arabidopsis thaliana]	275	1.00E-72	820	2	pfam03094
113	gi|33573112|emb|CAE36756.1| acriflavine resistance protein B [Bordetella parapertussis]	350	2.00E-95	613	2	pfam02355, PRK10555
114	gi|68344778|gb|AAY92384.1| undecaprenol kinase, putative [Pseudomonas fluorescens Pf-5]	263	6.00E-69	848	3	PRK00281
115	gi|47059739|gb|AAT09451.1| putative NBS-LRR type disease resistance protein [Prunus persica]	461	0	3281	15	pfam00931, COG4886
116	gi|56784769|dbj|BAD81990.1| putative terbinafine resistance locus protein [Oryza sativa Japonica Group]	174	1.00E-42	598	3	pfam04893
117	gi|50906405|ref|XP_464691.1| putative MAP3K delta-1 protein kinase [Oryza sativa (japonica cultivar-group)]	276	5.00E-73	850	4	cd00180, smart00220

118	gi|32400274|emb|CAE00640.1| putative mitogen-activated protein kinase 1 [Medicago sativa]	196	5.00E-49	702	2	cd00180, smart00220
119	gi|68345576|gb|AAY93182.1| outer membrane efflux protein [Pseudomonas fluorescens Pf-5]	310	2.00E-83	576	3	pfam02321, COG1538
120	gi|75704070|gb|ABA23746.1| ABC transporter-like [Anabaena variabilis ATCC 29413]	264	2.00E-69	598	2	cd03253, COG1132
121	gi|15220982|ref|NP_171690.1| PFC1 (PALEFACE 1) [Arabidopsis thaliana]	141	2.00E-37	687	2	PRK00274
122	gi|49533774|gb|AAT66773.1| putative late blight resistance protein [Solanum demissum]	313	1.00E-83	1266	5	pfam00931
123	gi|39636816|gb|AAR29076.1| blight resistance protein T118 [Solanum tarijense]	126	7.00E-30	768	4	pfam00931, COG4886
124	gi|34913374|ref|NP_918034.1| putative DNA damage repair protein [Oryza sativa]	201	2.00E-50	632	2	smart00361
125	gi|79328183|ref|NP_001031909.1| carboxylic ester hydrolase [Arabidopsis thaliana]	371	0	1328	14	pfam02230
126	gi|79328183|ref|NP_001031909.1| carboxylic ester hydrolase [Arabidopsis thaliana]	345	1.00E-93	868	6	pfam02230
128	gi|18394385|ref|NP_564003.1| ATP binding / kinase/ protein kinase/ protein [Arabidopsis thaliana]	404	0	760	2	cd00192
129	gi|14330718|emb|CAC40827.1| HcrVf3 protein [Malus floribunda]	145	8.00E-42	742	2	pfam08263, cd00116, COG4886
130	gi|15225286|ref|NP_180201.1| ER (ERECTA) [Arabidopsis thaliana]	179	2.00E-59	643	2	cd00180, pfam08263, cd00116, cd05086
131	gi|55139525|gb|AAV41396.1| peru 2 [Lycopersicon peruvianum]	194	2.00E-48	695	2	pfam08263
132	gi|14269079|gb|AAK58012.1| verticillium wilt disease resistance protein Ve2 [Lycopersicon esculentum]	165	2.00E-39	740	2	pfam08263, cd00116
133	gi|15235429|ref|NP_192169.1| calmodulin binding [Arabidopsis thaliana]	499	0	1828	4	pfam03094
134	gi|50945887|ref|XP_482471.1| putative disease resistance gene homolog [Oryza sativa (japonica cultivar-group)]	246	1.00E-63	1619	5	pfam00931, COG4886
135	gi|18478787|gb|AAL73330.1| putative receptor-like protein kinase RLPK1 [Glycine max]	101	2.00E-20	663	2	cd00192
136	gi|15225814|ref|NP_180259.1| ATPase, coupled to transmembrane movement of substances [Arabidopsis thaliana]	756	0	1725	6	cd03232, cd03233, pfam01061, pfam08370, cd03262, pfam00005
137	gi|79330795|ref|NP_001032070.1| MLO11; calmodulin binding [Arabidopsis thaliana]	183	3.00E-68	778	3	pfam03094
138	gi|28436071|gb|AAO41731.1| cytoplasmic ribosomal protein S14 [Brassica napus]	234	2.00E-60	826	11	PRK09607
139	gi|33637487|gb|AAQ23899.1| RSH2 [Nicotiana tabacum]	1100	0	2333	10	pfam04607, pfam01966
140	gi|15231046|ref|NP_191408.1| phosphoric ester hydrolase [Arabidopsis thaliana]	381	0	1145	4	cd03388
141	gi|48209881|gb|AAT40487.1| putative disease resistance protein [Solanum demissum]	217	5.00E-55	962	2	pfam00931
142	gi|77689672|ref|ZP_00804853.1| Peptidase M15D, vanX D-ala-D-ala dipeptidase [Rhodopseudomonas palustris BisB5]	119	1.00E-25	673	2	COG2173
143	gi|42733544|dbj|BAD11207.1| multidrug resistance-associated protein [Thlaspi caerulescens]	226	5.00E-72	665	2	cd03244, cd03250, pfam00664, cd03224, COG1132
144	gi|33576997|emb|CAE34077.1| putative ABC transporter [Bordetella bronchiseptica RB50]	212	3.00E-54	337	4	cd03249, pfam00664, COG1132
145	gi|82735577|ref|ZP_00898439.1| major facilitator family transporter [Pseudomonas putida F1]	414	0	690	6	COG2814, pfam0769
146	gi|50918091|ref|XP_469442.1| putative protein kinase [Oryza sativa (japonica cultivar-group)]	197	2.00E-49	740	2	cd00180, smart00220
147	gi|8547237|gb|AAF76312.1| Prf [Lycopersicon esculentum]	194	8.00E-48	1246	2	pfam00931
148	gi|10121909|gb|AAG13419.1| T7N9.24 [Arabidopsis thaliana]	132	4.00E-60	1059	2	smart00255, cd00116, pfam00931, COG4886
150	gi|27368843|emb|CAD59579.1| MDR-like ABC transporter [Oryza sativa (japonica cultivar-group)]	144	4.00E-53	1311	2	cd03249, pfam00664, COG1132
151	gi|49086916|gb|AAT51392.1| PA4599 [synthetic construct]	183	2.00E-45	501	3	PRK09578, pfam00529
152	gi|34909106|ref|NP_915900.1| putative NBS-LRR type resistance protein [Oryza sativa (japonica cultivar-group)]	100	5.00E-25	900	2	pfam00931, COG4886
153	gi|71040679|gb|AAZ20288.1| disease resistance-responsive family protein [Arachis hypogaea]	146	4.00E-34	612	2	pfam03018
154	gi|41052474|dbj|BAD07484.1| PDR-type ABC transporter 2 [Nicotiana tabacum]	380	0	835	3	cd03232, cd03233, pfam01061, pfam08370, pfam00005
155	gi|33572422|emb|CAE41988.1| muramoyltetrapeptide carboxypeptidase [Bordetella pertussis Tohama I]	211	6.00E-54	508	2	PRK11253
156	gi|32400274|emb|CAE00640.1| putative mitogen-activated protein kinase 1 [Medicago sativa]	139	7.00E-32	702	2	cd00180, smart00220
159	gi|48057628|gb|AAT39951.1| putative disease resistance protein Prf [Solanum demissum]	269	2.00E-70	1318	3	pfam00931
160	gi|22331862|ref|NP_191473.2| ATMRP14 [Arabidopsis thaliana]	318	3.00E-85	994	2	cd03244, cd03250, pfam00664, cd03296, COG1132
162	gi|77384019|gb|ABA75532.1| Drug resistance transporter EmrB/QacA subfamily [Pseudomonas fluorescens PfO-1]	323	3.00E-87	719	5	PRK10504, pfam07690
163	gi|18423435|ref|NP_568777.1| WRKY27; transcription factor [Arabidopsis thaliana]	140	5.00E-32	787	2	pfam03106
164	gi|4097585|gb|AAD09518.1| NTGP4 [Nicotiana tabacum]	355	2.00E-96	1384	9	cd01852, COG4717
165	gi|33571779|emb|CAE41286.1| acriflavine resistance protein B [Bordetella pertussis Tohama I]	145	5.00E-34	255	2	PRK10555
168	gi|71040679|gb|AAZ20288.1| disease resistance-responsive family protein [Arachis hypogaea]	161	3.00E-38	789	2	pfam03018
169	gi|15235429|ref|NP_192169.1| calmodulin binding [Arabidopsis thaliana]	661	0	1458	4	pfam03094
171	gi|38566726|emb|CAE76632.1| leucine rich repeat protein [Cicer arietinum]	500	0	1315	3	pfam08263, cd00116
172	gi|46389753|dbj|BAD15107.1| hypothetical protein [Nicotiana tabacum]	194	2.00E-56	1239	3	No CD has been identified
173	gi|15233846|ref|NP_194179.1| ATP binding / kinase/ protein kinase/ protein [Arabidopsis thaliana]	169	9.00E-41	733	2	cd00192
174	gi|34904948|ref|NP_913821.1| putative zinc transporter [Oryza sativa]	216	2.00E-54	1436	4	COG1230
175	gi|48209881|gb|AAT40487.1| putative disease resistance protein [Solanum demissum]	105	1.00E-21	675	2	pfam00931
176	gi|50900396|ref|XP_450986.1| putative ABC transporter [Oryza sativa (japonica cultivar-group)]	490	0	1415	6	cd03213, pfam00005, pfam01061
178	gi|12006354|gb|AAG44839.1| putative Hs1pro-1-like receptor [Glycine max]	344	3.00E-93	1029	7	pfam07014, pfam07231
180	gi|42602161|gb|AAS21681.1| receptor-like kinase [Arabidopsis thaliana]	265	3.00E-69	1304	2	cd00192, COG4886, smart00220
182	gi|15223013|ref|NP_177760.1| unknown protein [Arabidopsis thaliana]	269	6.00E-71	686	2	COG5070
183	gi|77689672|ref|ZP_00804853.1| Peptidase M15D, vanX D-ala-D-ala dipeptidase [Rhodopseudomonas palustris BisB5]	120	4.00E-26	684	7	COG2173

184	gi|66734028|gb|AAY53482.1| resistance protein R1 [Solanum demissum]	290	8.00E-77	1239	5	pfam00931
185	gi|47717725|gb|AAT37905.1| multidrug-resistance associated protein 3 [Zea mays]	350	3.00E-95	720	2	cd03244, cd03250, pfam00664, COG1132
187	gi|15230899|ref|NP_188597.1| catalytic/ iron ion binding [Arabidopsis thaliana]	379	0	1367	3	pfam04055, COG0820
188	gi|30687745|ref|NP_173637.3| unknown protein [Arabidopsis thaliana]	197	2.00E-60	770	2	cd00170, pfam03765
189	gi|8547232|gb|AAF76308.1| Prf [Lycopersicon pimpinellifolium]	196	1.00E-61	744	2	pfam00931
190	gi|30267436|gb|AAP21819.1| metal transporter [Lycopersicon esculentum]	783	0	2216	12	pfam01566
191	gi|34905796|ref|NP_914245.1| P0401G10.24 [Oryza sativa (japonica cultivar-group)]	132	1.00E-29	758	2	pfam03656
192	gi|68560019|ref|ZP_00599347.1| Heavy metal efflux pump CzcA [Ralstonia metallidurans CH34]	343	4.00E-93	656	2	COG3696
193	gi|24051889|gb|AAN43154.1| orf, conserved hypothetical protein [Shigella flexneri 2a str.301]	240	5.00E-62	817	7	PRK10995
194	gi|41052474|dbj|BAD07484.1| PDR-type ABC transporter 2 [Nicotiana tabacum]	917	0	2264	29	cd03232, cd03233, pfam01061, pfam08370, pfam00005
195	gi|82794018|gb|ABB91438.1| R-FOM-2 [Cucumis melo]	167	9.00E-40	1384	2	pfam00931
196	gi|15239535|ref|NP_197963.1| kinase/ protein binding [Arabidopsis thaliana]	104	4.00E-21	770	2	pfam08263, cd00116
197	gi|34910256|ref|NP_916475.1| putative MRP-like ABC transporter [Oryza sativa]	357	3.00E-97	788	2	cd03244, cd03250, pfam00664, COG1132
198	gi|22137208|gb|AAM91449.1| At1g53210/F12M16_12 [Arabidopsis thaliana]	306	6.00E-82	724	2	pfam01699, cd00051
199	gi|15231938|ref|NP_188102.1| leucine-rich repeat family protein / protein kinase family protein [Arabidopsis thaliana]	293	1.00E-77	1074	4	cd00192, cd00116
200	gi|12324080|gb|AAG52008.1| unknown protein; 23065-20358 [Arabidopsis thaliana]	236	8.00E-61	1037	3	pfam02535
202	gi|58826317|gb|AAW82883.1| phytoalexin-deficient 4-1 protein [Solanum tuberosum]	528	0	1506	5	cd00519
203	gi|14626935|gb|AAK70805.1| leucine-rich repeat resistance protein-like protein [Gossypium hirsutum]	531	0	1539	8	COG4886
204	gi|21552979|gb|AAM62409.1| Rar1 [Nicotiana tabacum]	349	7.00E-95	905	3	pfam04968
207	gi|15232624|ref|NP_190257.1| ATP binding [Arabidopsis thaliana]	254	8.00E-84	2331	8	pfam00931
208	gi|50939001|ref|XP_479028.1| putative disease resistance protein RPH8 [Oryza sativa (japonica cultivar-group)]	390	0	2376	4	pfam00931, COG4886
209	gi|42572237|ref|NP_974213.1| binding [Arabidopsis thaliana]	381	0	1176	3	cd00204, COG5184, pfam00415
210	gi|33568473|emb|CAE32385.1| putative multidrug resistance protein [Bordetella bronchiseptica RB50]	210	4.00E-53	691	2	PRK10476, COG1566
211	gi|48093465|gb|AAT40109.1| putative UDP-glucuronate decarboxylase 3 [Nicotiana tabacum]	147	6.00E-34	862	7	PRK10084, COG0451
212	gi|48057628|gb|AAT39951.1| putative disease resistance protein Prf [Solanum demissum]	412	0	1961	9	pfam00931
213	gi|55733942|gb|AAV59449.1| putative MRP-like ABC transporter [Oryza sativa]	298	0	1171	2	cd03244, cd03250, pfam00664, cd03297, COG1132, pfam00005
214	gi|26450926|dbj|BAC42570.1| putative receptor protein kinase [Arabidopsis thaliana]	286	7.00E-76	922	2	cd00192, pfam08263, COG4886, smart00220
215	gi|5231014|gb|AAD41050.1| NBS/LRR disease resistance protein RPM1 [Arabidopsis thaliana]	114	4.00E-24	694	2	pfam00931, COG4886
216	gi|82794018|gb|ABB91438.1| R-FOM-2 [Cucumis melo]	105	1.00E-21	672	2	pfam00931
218	gi|3334219|sp|O23920|HPPD_DAUCA 4-hydroxyphenylpyruvate dioxygenase (4HPPD) [Daucus carota]	233	8.00E-65	773	2	pfam00903
219	gi|62632825|gb|AAX89383.1| NBS-LRR type disease resistance protein Rps1-k-2 [Glycine max]	156	8.00E-37	848	2	pfam00931, COG4886
220	gi|41052472|dbj|BAD07483.1| PDR-type ABC transporter 1 [Nicotiana tabacum]	408	0	1677	4	cd03232, cd03233, cd03234, pfam01061, pfam08370, cd03226, pfam00005
221	gi|15229564|ref|NP_189044.1| unknown protein [Arabidopsis thaliana]	108	4.00E-23	382	2	pfam03018
222	gi|48209881|gb|AAT40487.1| putative disease resistance protein [Solanum demissum]	124	5.00E-27	802	2	pfam00931
223	gi|38489222|gb|AAR21296.1| NDR1-like protein [Nicotiana benthamiana]	206	6.00E-52	703	4	pfam07320
224	gi|50945887|ref|XP_482471.1| putative disease resistance gene homolog [Oryza sativa (japonica cultivar-group)]	207	1.00E-51	1693	3	pfam00931, COG4886
225	gi|30693847|ref|NP_190920.2| UXS1 (UDP-GLUCURONIC ACID DECARBOXYLASE 1); catalytic [Arabidopsis thaliana]	159	2.00E-37	855	2	PRK10084, COG0451
226	gi|52854207|gb|AAU88159.1| disease resistance-like protein [Coffea arabica]	261	1.00E-68	778	2	pfam00931
228	gi|68348514|gb|AAY96120.1| multidrug resistance transporter, Bcr/CflA family [Pseudomonas fluorescens Pf-5]	347	3.00E-94	860	9	PRK11102, pfam07690
229	gi|18414234|ref|NP_568119.1| CDC25 [Arabidopsis thaliana]	211	2.00E-53	765	5	cd01531
230	gi|44888877|gb|AAS48176.1| Osmolarity-sensing histidine-kinase [Pseudomonas fluorescens]	400	0	709	5	cd00075, pfam00672, PRK09467
231	gi|32364526|gb|AAP80292.1| resistance protein Tsu5 [Arabidopsis thaliana]	105	2.00E-21	902	2	pfam00931
232	gi|24459845|emb|CAC82610.1| disease resistance-like protein [Coffea arabica]	161	2.00E-58	1047	2	pfam00931
234	gi|15223416|ref|NP_171656.1| metal ion binding [Arabidopsis thaliana]	100	3.00E-20	640	2	pfam00403
235	gi|83283975|gb|ABC01895.1| transporter-like protein [Solanum tuberosum]	338	1.00E-91	804	4	cd00170, pfam03765
236	gi|29409364|gb|AAM29178.1| biostress-resistance-related protein [Triticum aestivum]	308	2.00E-82	840	2	pfam02230
237	gi|42562729|ref|NP_175747.2| serine/threonine protein kinase-related [Arabidopsis thaliana]	207	6.00E-52	1360	3	cd00192, cd00116
238	gi|18402572|ref|NP_566659.1| unknown protein [Arabidopsis thaliana]	374	0	1240	3	pfam02470, COG1463
239	gi|15230686|ref|NP_187915.1| ATMRP3 [Arabidopsis thaliana]	315	0	1282	2	cd03244, cd03250, pfam00664, COG1132, cd03224
241	gi|34907356|ref|NP_915025.1| putative receptor protein kinase [Oryza sativa]	348	1.00E-94	880	2	pfam08263, COG4886, cd00116, smart00220, cd00180
242	gi|62896437|emb|CAD91352.2| mercuric ion reductase MerA [Pseudomonas fluorescens]	142	3.00E-33	256	21	cd00371, pfam02852, pfam00070, COG1249, PRK10671
243	gi|8547237|gb|AAF76312.1| Prf [Lycopersicon esculentum]	233	1.00E-59	1114	4	pfam00931
244	gi|33238810|gb|AAQ00875.1| PLP-dependent enzyme [Prochlorococcus marinus str. CCMP1375]	207	2.00E-84	1558	3	pfam06838
245	gi|48093461|gb|AAT40107.1| UDP-glucuronate decarboxylase 1 [Nicotiana tabacum]	658	0	1437	10	PRK11908, COG0451
246	gi|4689223|gb|AAD27815.1| disease resistance protein I2 [Lycopersicon esculentum]	130	6.00E-29	715	2	pfam00931
247	gi|15235205|ref|NP_193719.1| ATPP2-A1 [Arabidopsis thaliana]	147	5.00E-34	1009	5	No CD has been identified
248	gi|77380404|gb|ABA71917.1| Secretion protein HlyD [Pseudomonas fluorescens PfO-1]	146	7.00E-34	805	11	PRK10559, COG1566
249	gi|7677046|gb|AAF67003.1| putative Hs1pro-1 homolog [Pisum sativum]	188	1.00E-46	611	2	pfam07014, pfam07231

250	gi|55771369|dbj|BAD72536.1| LIM domain containing protein-like [Oryza sativa]	438	0	885	3 smart00132
251	gi|47059739|gb|AAT09451.1| putative NBS-LRR type disease resistance protein [Prunus persica]	201	5.00E-50	1140	4 pfam00931, COG4886
252	gi|53727578|ref|ZP_00347912.1| COG1566: Multidrug resistance efflux pump [Pseudomonas aeruginosa UCBPP-PA14]	238	2.00E-61	764	12 COG1566, PRK10476
253	gi|48209881|gb|AAT40487.1| putative disease resistance protein [Solanum demissum]	148	2.00E-34	729	2 pfam00931
254	gi|5734781|gb|AAD50046.1| Very similar to disease resistance proteins [Arabidopsis thaliana]	142	7.00E-33	655	2 pfam00931
255	gi|55773694|dbj|BAD72277.1| 40S ribosomal protein S30-like [Oryza sativa]	103	2.00E-21	476	2 pfam04758
257	gi|68342709|gb|AAY90315.1| multidrug efflux RND transporter [Pseudomonas fluorescens Pf-5]	348	1.00E-94	885	15 pfam02355, PRK09579
258	gi|24459853|emb|CAC82602.1| disease resistance-like protein [Coffea arabica]	300	9.00E-80	1131	5 pfam00931
259	gi|30698733|ref|NP_177218.3| ATTAP1; ATPase, coupled to transmembrane movement of substances [Arabidopsis thaliana]	454	0	899	3 cd03249, pfam00664, COG1132
262	gi|3894387|gb|AAC78593.1| Hcr2-0B [Lycopersicon esculentum]	127	4.00E-28	720	2 cd00116, pfam08263
264	gi|82794018|gb|ABB91438.1| R-FOM-2 [Cucumis melo]	104	8.00E-21	1558	3 pfam00931
265	gi|46389753|dbj|BAD15107.1| hypothetical protein [Nicotiana tabacum]	216	2.00E-54	1229	4 No CD has been identified
266	gi|30698151|ref|NP_201372.2| ATP binding / kinase/ protein serine/threonine kinase [Arabidopsis thaliana]	200	1.00E-83	1204	3 cd00116, pfam08263, COG4886, cd00180
267	gi|77967250|gb|ABB08630.1| Acriflavin resistance protein [Burkholderia sp. 383]	105	1.00E-21	593	3 COG0841
268	gi|50899184|ref|XP_450380.1| putative disease related protein 2 [Oryza sativa]	138	2.00E-65	1886	4 pfam00931, COG4886
270	gi|33564744|emb|CAE44069.1| AcrB/AcrD/AcrF family protein [Bordetella pertussis Tohama I]	147	4.00E-34	643	4 PRK10503
271	gi|15235205|ref|NP_193719.1| ATPP2-A1 [Arabidopsis thaliana]	150	3.00E-35	689	2 No CD has been identified
272	gi|15217785|ref|NP_171753.1| ATPase, coupled to transmembrane movement of substances [Arabidopsis thaliana]	321	2.00E-86	804	5 cd03249, pfam00664, COG1132
273	gi|46389753|dbj|BAD15107.1| hypothetical protein [Nicotiana tabacum]	139	2.00E-31	934	3 No CD has been identified
274	gi|50945889|ref|XP_482472.1| putative disease resistance gene homolog [Oryza sativa (japonica cultivar-group)]	145	1.00E-33	779	4 pfam00931, COG4886
275	gi|66737320|gb|AAY54606.1| NRG1 [Nicotiana benthamiana]	224	4.00E-57	936	2 pfam05659, pfam00931, COG4886
276	gi|49533774|gb|AAT66773.1| putative late blight resistance protein, identical [Solanum demissum]	164	3.00E-39	786	2 pfam00931
277	gi|14715462|dbj|BAB62040.1| CjMDR1 [Coptis japonica]	645	0	1569	12 cd03249, pfam00664, COG1132
278	gi|30690355|ref|NP_850885.1| D111/G-patch domain-containing protein [Arabidopsis thaliana]	168	2.00E-40	774	2 smart00443
279	gi|8547237|gb|AAF76312.1| Prf [Lycopersicon esculentum]	105	4.00E-21	1325	3 pfam00931
280	gi|22331862|ref|NP_191473.2| ATMRP14 [Arabidopsis thaliana]	481	0	1093	4 cd03244, cd03250, pfam00664, cd03296, COG1132
281	gi|48057628|gb|AAT39951.1| putative disease resistance protein Prf [Solanum demissum]	205	2.00E-51	1058	2 pfam00931
282	gi|79319849|ref|NP_001031180.1| unknown protein [Arabidopsis thaliana]	115	2.00E-49	1167	4 pfam04859
283	gi|21239382|gb|AAM44274.1| receptor-like kinase RHG1 [Glycine max]	375	0	793	2 smart00220, pfam08263, COG4886, cd00180
285	gi|18408274|ref|NP_564850.1| ATNAP11 [Arabidopsis thaliana]	466	0	1579	5 cd03261, COG1127
286	gi|34913790|ref|NP_918242.1| putative LRR [Oryza sativa (japonica cultivar-group)]	143	2.00E-34	799	2 pfam00931, COG4886
287	gi|10177549|dbj|BAB10828.1| ABC transporter-like protein [Arabidopsis thaliana]	341	2.00E-92	824	2 cd03249, pfam00664, COG1132
289	gi|24984168|gb|AAN68204.1| ABC transporter, permease/ATP-binding protein, putative [Pseudomonas putida KT2440]	231	2.00E-59	589	11 cd03253,pfam00664, cd03223, COG5265
290	gi|82735577|ref|ZP_00898439.1| major facilitator family transporter [Pseudomonas putida F1]	417	0	699	6 COG2814, pfam07690
291	gi|16974114|emb|CAC95155.1| putative resistance protein [Solanum lycopersicum]	469	0	1466	9 pfam03798
292	gi|55297132|dbj|BAD68775.1| putative NBS-LRR disease resistance protein [Oryza sativa Japonica Group]	124	4.00E-27	814	3 pfam00931
293	gi|53689783|gb|AAU89792.1| late blight resistance protein-like [Solanum tuberosum]	152	1.00E-35	803	2 pfam00931, smart00534
294	gi|77381310|gb|ABA72823.1| Acriflavin resistance protein [Pseudomonas fluorescens PfO-1]	404	0	781	5 COG0841
295	gi|2292907|emb|CAA71179.1| P-glycoprotein homologue [Hordeum vulgare]	438	0	1166	3 cd03249, pfam00664, COG1132, cd03271
296	gi|52854237|gb|AAU88174.1| disease resistance-like protein [Psilanthus wightianus]	143	1.00E-32	1237	3 pfam00931
297	gi|39636816|gb|AAR29076.1| blight resistance protein T118 [Solanum tarijense]	172	2.00E-41	1124	2 pfam00931, COG4886
298	gi|42570865|ref|NP_973506.1| SAE2 (SUMO-ACTIVATING ENZYME 2) [Arabidopsis thaliana]	286	8.00E-76	818	4 cd01489
299	gi|34104782|gb|AAQ61139.1| probable manganese transport transmembrane protein [Chromobacterium violaceum ATCC 12472]	178	1.00E-43	620	4 COG1914
301	gi|82568697|dbj|BAE48662.1| alcohol dehydrogenase [Prunus mume]	172	3.00E-42	509	2 PRK12825, PRK05653
302	gi|8547237|gb|AAF76312.1| Prf [Lycopersicon esculentum]	411	0	1667	5 pfam00931
303	gi|15220948|ref|NP_172851.1| GN (GNOM) [Arabidopsis thaliana]	365	0	942	2 cd00171, COG5307
304	gi|6671365|gb|AAF23176.1| P-glycoprotein [Gossypium hirsutum]	456	0	908	3 cd03249, pfam00664, COG1132, cd03270
305	gi|42567508|ref|NP_195551.2| antiporter/ drug transporter [Arabidopsis thaliana]	251	2.00E-65	851	2 pfam01554
306	gi|62734129|gb|AAX96238.1| NB-ARC domain, putative [Oryza sativa (japonica cultivar-group)]	111	5.00E-23	1259	3 pfam00931
307	gi|47572215|ref|ZP_00242260.1| COG0798: Arsenite efflux pump ACR3 and related permeases [Rubrivivax gelatinosus PM1]	107	1.00E-22	196	4 COG0798
308	gi|34910752|ref|NP_916723.1| P0042A10.5 [Oryza sativa (japonica cultivar-group)]	499	0	1353	2 COG5184, pfam00415
309	gi|15237733|ref|NP_201260.1| unknown protein [Arabidopsis thaliana]	463	0	835	6 No CD has been identified
310	gi|18398444|ref|NP_565416.1| MLO8; calmodulin binding [Arabidopsis thaliana]	602	0	1672	8 pfam03094
311	gi|18400769|ref|NP_566512.1| Ran GTPase binding [Arabidopsis thaliana]	433	0	1028	2 COG5184
312	gi|4204793|gb|AAD10836.1| P-glycoprotein [Solanum tuberosum]	457	0	944	5 cd03249, pfam00664, COG1132
313	gi|15230349|ref|NP_188563.1| protein binding [Arabidopsis thaliana]	206	6.00E-60	1107	4 cd00116
315	gi|15222893|ref|NP_175437.1| ATP binding [Arabidopsis thaliana]	108	2.00E-22	711	2 pfam00931
316	gi|28436071|gb|AAO41731.1| cytoplasmic ribosomal protein S14 [Brassica napus]	232	1.00E-59	889	13 PRK09607
317	gi|14269079|gb|AAK58012.1| verticillium wilt disease resistance protein Ve2	369	0	1255	3 cd00116, pfam08263

	[Lycopersicon esculentum]					
318	gi|15220604|ref|NP_176961.1| ATNAP3 [Arabidopsis thaliana]	361	3.00E-98	1361	11	cd03260, COG1126
319	gi|30267434|gb|AAP21818.1| root-specific metal transporter [Lycopersicon esculentum]	333	5.00E-90	785	2	pfam01566
321	gi|15222893|ref|NP_175437.1| ATP binding [Arabidopsis thaliana]	338	4.00E-91	1906	5	pfam00931
322	gi|42550761|gb|EAA73604.1| hypothetical protein FG04278.1 [Gibberella zeae PH-1]	294	0	733	2	pfam03452
323	gi|50929779|ref|XP_474417.1| OSJNBa0088H09.13 [Oryza sativa (japonica cultivar-group)]	206	2.00E-55	820	4	pfam04939
324	gi|6456755|gb|AAF09256.1| disease resistance protein BS2 [Capsicum chacoense]	216	8.00E-98	2019	6	pfam00931
325	gi|3288065|emb|CAB09800.1| RisS protein [Bordetella bronchiseptica]	391	0	725	2	cd00075, smart00304, smart00388, COG3920
326	gi|15220149|ref|NP_175157.1| NRAMP2; metal ion transporter [Arabidopsis thaliana]	620	0	1205	3	pfam01566
327	gi|7110565|gb|AAF36987.1| viral resistance protein [Arabidopsis thaliana]	125	3.00E-27	1054	2	pfam00931
328	gi|15219396|ref|NP_173131.1| unknown protein [Arabidopsis thaliana]	245	1.00E-70	972	4	No CD has been identified
329	gi|18408943|ref|NP_564921.1| IAR1 (IAA-ALANINE RESISTANT 1); metal ion transporter [Arabidopsis thaliana]	352	1.00E-95	1102	2	pfam02535
330	gi|22652532|gb|AAN03742.1| NBS-LRR-like protein [Oryza sativa (japonica cultivar-group)]	222	6.00E-56	2251	6	pfam00931
331	gi|34911420|ref|NP_917057.1| putative leucine rich repeat containing protein kinase [Oryza sativa (japonica cultivar-group)]	265	1.00E-69	706	2	cd00192, COG4886
332	gi|15218909|ref|NP_176187.1| ATP binding [Arabidopsis thaliana]	181	7.00E-44	1580	3	pfam00931
333	gi|77964582|gb|ABB05963.1| Drug resistance transporter, EmrB/QacA subfamily [Burkholderia sp. 383]	280	2.00E-74	499	2	PRK10504, pfam07690
334	gi|20466556|gb|AAM20595.1| integral membrane protein, putative [Arabidopsis thaliana]	332	8.00E-90	751	3	pfam01554, COG0534
335	gi|79317904|ref|NP_001031037.1| unknown protein [Arabidopsis thaliana]	166	1.00E-39	1104	2	pfam03798
336	gi|66737320|gb|AAY54606.1| NRG1 [Nicotiana benthamiana]	210	6.00E-53	870	3	pfam05659, pfam00931, COG4886
338	gi|77551506|gb|ABA94303.1| AWJL218 protein [Oryza sativa (japonica cultivar-group)]	311	5.00E-83	1800	3	pfam08263, COG4886
339	gi|48057628|gb|AAT39951.1| putative disease resistance protein Prf [Solanum demissum]	247	8.00E-64	1290	3	pfam00931
340	gi|50945887|ref|XP_482471.1| putative disease resistance gene homolog [Oryza sativa (japonica cultivar-group)]	145	1.00E-51	1558	4	pfam00931, COG4886
341	gi|48057628|gb|AAT39951.1| putative disease resistance protein Prf [Solanum demissum]	241	1.00E-61	2213	8	pfam00931
343	gi|71081904|gb|AAZ23261.1| senescence-associated protein [Nicotiana tabacum]	211	4.00E-53	966	3	cd00158
344	gi|48209881|gb|AAT40487.1| putative disease resistance protein [Solanum demissum]	146	6.00E-34	639	2	pfam00931
345	gi|42571555|ref|NP_973868.1| TGD1 (TRIGALACTOSYLDIACYLGLYCEROL 1) [Arabidopsis thaliana]	474	0	1390	5	pfam02405
346	gi|14331118|emb|CAC40990.1| ABC1 protein [Nicotiana plumbaginifolia]	133	6.00E-30	683	3	cd03232, cd03233, cd03234, pfam01061, pfam08370, cd03259, cd03247
347	gi|71081904|gb|AAZ23261.1| senescence-associated protein [Nicotiana tabacum]	212	1.00E-53	817	4	cd00158
