# Supplementary material for: In silico identification of coffee genome expressed sequences potentially associated with resistance to diseases
Source: Genet Mol Biol. 2010 Dec 1;33(4):795–806. doi: 10.1590/s1415-47572010000400031 (PMC3036153; doi:10.1590/s1415-47572010000400031)
Supplement: Table S3 — EST-contigs with E-values < e-20 and scores > 100 obtained in the project Hypersensitive, and their blast hits, scores, E-values, sizes, number of reads and conserved domains from putative proteins. [file gmb-33-4-795-suppl3.pdf]

Table S3: EST-Contigs with e-value < e^{-20} and score > 100 obtained in the Project Hypersensitive, and their blast hit, score, e-value, size, number of reads, and conserved domains from putative proteins.

Hypersensitive						
Contig	BLAST NR	Score	e-value	Length	Reads	Conserved Domains
1	gi|21386975|gb|AAM47891.1| hypersensitive-induced response protein [Arabidopsis thaliana]	199	2.00E-49	1075	4	cd03407
2	gi|34484310|gb|AAQ72788.1| hypersensitive-induced response protein [Cucumis sativus]	487	0	1576	16	cd03407
3	gi|11994123|dbj|BAB01125.1| unnamed protein product [Arabidopsis thaliana]	242	3.00E-66	1008	5	cd00128
4	gi|21386975|gb|AAM47891.1| hypersensitive-induced response protein [Arabidopsis thaliana]	475	0	1241	6	cd03407
5	gi|48209898|gb|AAT40492.1| putative hypersensitive-induced reaction protein [Solanum demissum]	514	0	1296	24	cd03407
6	gi|15450553|gb|AAK96454.1| AT5g49480/K6M13_2 [Arabidopsis thaliana]	150	5.00E-35	805	6	cd00051
7	gi|46452120|gb|AAS98165.1| hypersensitive-induced reaction protein [Capsicum annuum]	520	0	1118	19	cd03407
8	gi|34484310|gb|AAQ72788.1| hypersensitive-induced response protein [Cucumis sativus]	139	8.00E-32	790	2	cd03407
