# Supplementary material for: In silico identification of coffee genome expressed sequences potentially associated with resistance to diseases
Source: Genet Mol Biol. 2010 Dec 1;33(4):795–806. doi: 10.1590/s1415-47572010000400031 (PMC3036153; doi:10.1590/s1415-47572010000400031)
Supplement: Table S4 — EST-contigs with E-values < e-20 and scores > 100 obtained in the project Pathogenesis, and their blast hits, scores, E-values, sizes, number of reads and conserved domains from putative proteins. [file gmb-33-4-795-suppl4.pdf]

Table S4: EST-Contigs with e-value < e^{-20} and score > 100 obtained in the Project Pathogenesis, and their blast hit, score, e-value, size, number of reads, and conserved domains from putative proteins.

Pathogenesis						
Contig	BLAST NR	Score	e-value	Length	Reads	Conserved Domains
1	gi|467689|emb|CAA55128.1| chitinase/lysozyme [Nicotiana tabacum]	284	9.00E-79	808	2	smart00636, pfam00704
2	gi|60280807|gb|AAX18296.1| major allergen Mal d 1.0501 [Malus x domestica]	166	8.00E-40	807	13	pfam00407
3	gi|603882|emb|CAA87072.1| pathogenesis-related protein PR-3 type [Sambucus nigra]	362	6.00E-99	804	3	cd00325, cd00035
4	gi|45773916|gb|AAS76762.1| At3g14067 [Arabidopsis thaliana]	127	3.00E-28	774	3	cd02120, pfam00082, pfam05922
5	gi|3413481|emb|CAA07250.1| serine protease [Lycopersicon esculentum]	218	2.00E-67	787	2	cd02120, pfam00082, pfam05922
6	gi|32401255|gb|AAP80801.1| class VII chitinase precursor [Gossypium hirsutum]	198	2.00E-49	713	3	cd00325
7	gi|21592749|gb|AAM64698.1| putative thaumatin-like protein [Arabidopsis thaliana]	325	2.00E-87	1592	3	smart00205
9	gi|6723683|emb|CAB67120.1| subtilisin-like protease [Lycopersicon esculentum]	230	3.00E-59	754	3	cd02120, pfam00082, pfam05922
10	gi|21592749|gb|AAM64698.1| putative thaumatin-like protein [Arabidopsis thaliana]	304	3.00E-81	911	7	smart00205
11	gi|4586372|dbj|BAA74546.2| thaumatin-like protein SE39b [Nicotiana tabacum]	313	5.00E-84	1011	4	smart00205
12	gi|7531180|sp|O04681|PTI5_LYCES Pathogenesis-related genes transcriptional activator PTI5 (PTO-interacting protein 5) [Lycopersicon esculentum]	158	2.00E-37	819	6	cd00018
13	gi|60280803|gb|AAX18294.1| major allergen Mal d 1.04 [Malus x domestica]	188	1.00E-46	735	2	pfam00407
14	gi|60280807|gb|AAX18296.1| major allergen Mal d 1.0501 [Malus x domestica]	184	6.00E-45	1068	81	pfam00407
15	gi|60280807|gb|AAX18296.1| major allergen Mal d 1.0501 [Malus x domestica]	166	1.00E-39	993	183	pfam00407
16	gi|60280803|gb|AAX18294.1| major allergen Mal d 1.04 [Malus x domestica]	168	2.00E-40	777	4	pfam00407
17	gi|40824063|gb|AAR92335.1| At1g12440 [Arabidopsis thaliana]	160	9.00E-38	1030	12	pfam01428
18	gi|60280803|gb|AAX18294.1| major allergen Mal d 1.04 [Malus x domestica]	165	2.00E-39	824	13	pfam00407
19	gi|60280803|gb|AAX18294.1| major allergen Mal d 1.04 [Malus x domestica]	163	7.00E-39	810	2	pfam00407
20	gi|7269429|emb|CAB79433.1| putative pathogenesis-related protein [Arabidopsis thaliana]	236	9.00E-61	975	3	cd00168
21	gi|60280807|gb|AAX18296.1| major allergen Mal d 1.0501 [Malus x domestica]	183	7.00E-45	830	15	pfam00407
22	gi|60280807|gb|AAX18296.1| major allergen Mal d 1.0501 [Malus x domestica]	166	8.00E-40	803	3	pfam00407
23	gi|71057064|emb|CAI38795.2| thaumatin-like protein [Actinidia deliciosa]	400	0	1017	41	pfam00314
24	gi|34897932|ref|NP_910312.1| ozone-responsive stress-related protein-like [Oryza sativa (japonica cultivar-group)]	138	1.00E-31	572	8	pfam06592
25	gi|53830843|gb|AAU95244.1| putative thaumatin-like protein [Solanum tuberosum]	371	0	975	56	pfam00314
26	gi|71057064|emb|CAI38795.2| thaumatin-like protein [Actinidia deliciosa]	404	0	892	15	pfam00314
27	gi|19976|emb|CAA41439.1| pathogenesis-related protein P2 [Lycopersicon esculentum]	216	4.00E-55	652	2	pfam00967
28	gi|33413141|emb|CAD60273.1| putative pathogenesis related protein 1 precursor [Vitis vinifera]	241	2.00E-62	761	9	cd00168
29	gi|33413141|emb|CAD60273.1| putative pathogenesis related protein 1 precursor [Vitis vinifera]	212	9.00E-54	818	28	cd00168
30	gi|33329390|gb|AAQ10092.1| thaumatin-like protein [Vitis vinifera]	381	0	1813	117	pfam00314
31	gi|14290153|gb|AAK59278.1| thaumatin-like protein [Sambucus nigra]	368	0	880	21	pfam00314
32	gi|60280803|gb|AAX18294.1| major allergen Mal d 1.04 [Malus x domestica]	166	1.00E-39	771	14	pfam00407
33	gi|33413141|emb|CAD60273.1| putative pathogenesis related protein 1 precursor [Vitis vinifera]	213	5.00E-54	657	2	cd00168
34	gi|2677826|gb|AAB97141.1| major allergen protein homolog [Prunus armeniaca]	147	4.00E-34	902	4	pfam00407
35	gi|3901018|emb|CAA10235.1| stress and pathogenesis-related protein [Fagus sylvatica]	214	4.00E-54	822	4	pfam00407
36	gi|6273385|gb|AAF06347.1| SCUTL2 [Vitis vinifera]	228	5.00E-84	925	10	smart00205
37	gi|7531180|sp|O04681|PTI5_LYCES Pathogenesis-related genes transcriptional activator PTI5 (PTO-interacting protein 5) [Lycopersicon esculentum]	172	1.00E-41	1047	3	cd00018
38	gi|32815927|gb|AAP88348.1| At3g12630 [Arabidopsis thaliana]	154	6.00E-36	1280	13	pfam01428
39	gi|4586372|dbj|BAA74546.2| thaumatin-like protein SE39b [Nicotiana tabacum]	281	2.00E-79	1037	3	smart00205
40	gi|30424403|emb|CAD56465.1| putative class 5 chitinase [Medicago truncatula]	219	1.00E-55	867	3	smart00636, pfam00704
41	gi|15221033|ref|NP_173261.1| thaumatin, putative [Arabidopsis thaliana]	399	0	1147	6	smart00205
42	gi|12324220|gb|AAG52086.1| thaumatin-like protein; 9376-10898 [Arabidopsis thaliana]	314	0	858	7	smart00205
43	gi|1236785|emb|CAA57976.1| sts14 [Solanum tuberosum]	175	6.00E-43	507	2	cd00168
44	gi|2501182|sp|Q41350|OLP1_LYCES Osmotin-like protein precursor [Lycopersicon esculentum]	413	0	1133	8	smart00205
45	gi|4510345|gb|AAD21434.1| expressed protein [Arabidopsis thaliana]	166	1.00E-39	811	4	pfam01428
46	gi|5814093|gb|AAD52097.1| receptor-like kinase CHRK1 [Nicotiana tabacum]	250	4.00E-65	812	3	cd00192, smart00636, pfam00704
48	gi|505267|emb|CAA54374.1| chitinase, class V [Nicotiana tabacum]	409	0	1346	15	smart00636, pfam00704
49	gi|50948973|ref|XP_493844.1| putative glutathione S-transferase [Oryza sativa]	115	2.00E-24	781	2	COG0625, cd03055, cd03203
50	gi|60280803|gb|AAX18294.1| major allergen Mal d 1.04 [Malus x domestica]	164	3.00E-39	938	91	pfam00407
51	gi|169363|gb|AAA33773.1| PVPR3 [Phaseolus vulgaris]	139	2.00E-31	910	3	pfam01428
52	gi|60280803|gb|AAX18294.1| major allergen Mal d 1.04 [Malus x domestica]	170	5.00E-41	801	8	pfam00407
53	gi|2677826|gb|AAB97141.1| major allergen protein homolog [Prunus armeniaca]	149	1.00E-34	818	4	pfam00407
54	gi|60280803|gb|AAX18294.1| major allergen Mal d 1.04 [Malus x domestica]	193	6.00E-48	772	2	pfam00407
55	gi|3183991|emb|CAA06414.1| P69F protein [Lycopersicon esculentum]	159	7.00E-38	757	2	cd02120, pfam00082, pfam05922
56	gi|2677826|gb|AAB97141.1| major allergen protein homolog [Prunus armeniaca]	167	5.00E-40	880	18	pfam00407
57	gi|38603816|gb|AAR24653.1| At5g40020 [Arabidopsis thaliana]	148	3.00E-34	890	2	smart00205
58	gi|11385441|gb|AAG34803.1| glutathione S-transferase GST 13 [Glycine max]	248	3.00E-64	1006	5	cd03185, cd03058
59	gi|7106538|dbj|BAA92224.1| similar to PR-10 [Vigna unguiculata]	197	5.00E-49	913	10	pfam00407
60	gi|21595167|gb|AAM66077.1| pathogenesis-related protein-like protein [Arabidopsis thaliana]	114	1.00E-24	515	3	No CD has been identified
61	gi|7270553|emb|CAB81510.1| thaumatin-like protein [Arabidopsis thaliana]	343	6.00E-93	870	4	smart00205
62	gi|60280803|gb|AAX18294.1| major allergen Mal d 1.04 [Malus x domestica]	164	4.00E-39	925	13	pfam00407
63	gi|12323299|gb|AAG51631.1| thaumatin-like protein; 12104-13574 [Arabidopsis thaliana]	261	2.00E-68	765	2	smart00205
