# Supplementary material for: In silico identification of coffee genome expressed sequences potentially associated with resistance to diseases
Source: Genet Mol Biol. 2010 Dec 1;33(4):795–806. doi: 10.1590/s1415-47572010000400031 (PMC3036153; doi:10.1590/s1415-47572010000400031)
Supplement: Table S5 — EST-contigs with E-values < e-20 and scores > 100 obtained in the project Chitinase, and their blast hits, scores, E-values, sizes, number of reads and conserved domains from putative proteins [file gmb-33-4-795-suppl5.pdf]

Table S5: EST-Contigs with e-value < e^{-20} and score > 100 obtained in the Project Chitinase, and their blast hit, score, e-value, size, number of reads, and conserved domains from putative proteins.

Chitinase						
**Contig**	**BLAST NR**	**Score**	**e-value**	**Length**	**Reads**	**Conserved Domains**
1	gi|467689|emb|CAA55128.1| chitinase/lysozyme [Nicotiana tabacum]	284	9.00E-79	808	2	smart00636, pfam00704
2	gi|15553476|gb|AAL01886.1| chitinase 3-like protein precursor [Trichosanthes kirilowii]	135	9.00E-35	843	2	pfam00704
3	gi|15553476|gb|AAL01886.1| chitinase 3-like protein precursor [Trichosanthes kirilowii]	320	5.00E-86	1364	56	pfam00704
4	gi|603882|emb|CAA87072.1| pathogenesis-related protein PR-3 type [Sambucus nigra]	362	6.00E-99	804	3	cd00325, cd00035
5	gi|15553476|gb|AAL01886.1| chitinase 3-like protein precursor [Trichosanthes kirilowii]	231	4.00E-59	1335	288	pfam00704
6	gi|3451147|emb|CAA09110.1| chitinase [Hevea brasiliensis]	357	1.00E-96	1880	58	COG3469
7	gi|11262118|pir||JC7335 chitinase (EC 3.2.1.14) 1 - cone shell (Conus tulipa) [Tulipa bakeri]	344	2.00E-93	851	3	smart00636
8	gi|15553476|gb|AAL01886.1| chitinase 3-like protein precursor [Trichosanthes kirilowii]	163	6.00E-39	880	19	pfam00704
9	gi|3790355|dbj|BAA33971.1| chitinase 134 [Nicotiana tabacum]	450	0	1007	11	cd00035
10	gi|37051096|dbj|BAC81645.1| class1 chitinase [Pisum sativum]	473	0	1377	48	cd00325
11	gi|3790355|dbj|BAA33971.1| chitinase 134 [Nicotiana tabacum]	457	0	1050	19	cd00035
12	gi|15553476|gb|AAL01886.1| chitinase 3-like protein precursor [Trichosanthes kirilowii]	194	4.00E-48	922	35	pfam00704
13	gi|15553476|gb|AAL01886.1| chitinase 3-like protein precursor [Trichosanthes kirilowii]	224	9.00E-57	1624	62	pfam00704
14	gi|10954033|gb|AAG25709.1| class III acidic chitinase [Malus x domestica]	273	5.00E-72	729	3	COG3469
15	gi|15553476|gb|AAL01886.1| chitinase 3-like protein precursor [Trichosanthes kirilowii]	206	7.00E-52	829	10	pfam00704
16	gi|15553476|gb|AAL01886.1| chitinase 3-like protein precursor [Trichosanthes kirilowii]	335	2.00E-90	1499	282	pfam00704
17	gi|1359600|emb|CAA64868.1| chitinase Ib [Castanea sativa]	457	0	1178	11	cd00325
18	gi|167539|gb|AAC37395.1| chitinase [Cucumis sativus]	162	2.00E-38	821	6	COG3469, pfam00704
19	gi|15553476|gb|AAL01886.1| chitinase 3-like protein precursor [Trichosanthes kirilowii]	339	1.00E-91	1295	155	pfam00704
20	gi|33414050|gb|AAP03087.1| class Ib chitinase [Galega orientalis]	285	2.00E-85	866	5	cd00325, cd00035
22	gi|15553476|gb|AAL01886.1| chitinase 3-like protein precursor [Trichosanthes kirilowii]	219	1.00E-55	932	4	pfam00704
23	gi|167540|gb|AAC37396.1| ORF 3 [Cucumis sativus]	222	2.00E-56	1142	7	COG3469
24	gi|4835586|dbj|BAA77677.1| acidic chitinase [Glycine max]	268	2.00E-70	1072	8	COG3469
25	gi|15553476|gb|AAL01886.1| chitinase 3-like protein precursor [Trichosanthes kirilowii]	152	1.00E-35	892	5	pfam00704
26	gi|30424403|emb|CAD56465.1| putative class 5 chitinase [Medicago truncatula]	219	1.00E-55	867	3	smart00636, pfam00704
27	gi|15553476|gb|AAL01886.1| chitinase 3-like protein precursor [Trichosanthes kirilowii]	228	4.00E-58	1391	136	pfam00704
28	gi|3451147|emb|CAA09110.1| chitinase [Hevea brasiliensis]	345	1.00E-93	994	2	COG3469
29	gi|34908492|ref|NP_915593.1| putative beta-1,3-glucanase [Oryza sativa]	400	0	1068	3	pfam00332
30	gi|762879|dbj|BAA08708.1| chitinase [Psophocarpus tetragonolobus]	155	7.00E-37	619	3	COG3469
31	gi|37051096|dbj|BAC81645.1| class1 chitinase [Pisum sativum]	439	0	1130	12	cd00325
32	gi|167538|gb|AAC37394.1| ORF 1 [Cucumis sativus]	160	2.00E-38	681	3	COG3469
33	gi|5814093|gb|AAD52097.1| receptor-like kinase CHRK1 [Nicotiana tabacum]	250	4.00E-65	812	3	cd00192, smart00636, pfam00704
34	gi|7434972|pir||JE0184 chitinase (EC 3.2.1.14) 2 - cone shell (Conus tulipa) [Tulipa bakeri]	382	0	1071	4	smart00636
35	gi|62999433|gb|AAY25165.1| beta-1,3-glucanase 1 [Ziziphus jujuba]	442	0	932	2	pfam00332
36	gi|505267|emb|CAA54374.1| chitinase, class V [Nicotiana tabacum]	409	0	1346	15	smart00636, pfam00704
37	gi|3790355|dbj|BAA33971.1| chitinase 134 [Nicotiana tabacum]	392	0	859	19	cd00035
38	gi|19775|emb|CAA77656.1| acidic chitinase III [Nicotiana tabacum]	377	0	1244	12	COG3469
40	gi|10954033|gb|AAG25709.1| class III acidic chitinase [Malus x domestica]	239	1.00E-61	1064	12	COG3469
41	gi|15553476|gb|AAL01886.1| chitinase 3-like protein precursor [Trichosanthes kirilowii]	223	1.00E-56	1113	6	pfam00704
42	gi|4835586|dbj|BAA77677.1| acidic chitinase [Glycine max]	160	4.00E-38	760	2	COG3469
43	gi|3790355|dbj|BAA33971.1| chitinase 134 [Nicotiana tabacum]	414	0	936	15	cd00035
44	gi|21592759|gb|AAM64708.1| unknown [Arabidopsis thaliana]	177	3.00E-52	1030	3	cd00030
45	gi|432580|gb|AAB28479.1| acidic class III chitinase SE2 [Beta vulgaris]	389	0	1067	21	COG3469
46	gi|2934696|dbj|BAA25015.1| class III acidic endochitinase [Glycine max]	111	1.00E-23	581	2	COG3469
47	gi|4835586|dbj|BAA77677.1| acidic chitinase [Glycine max]	270	2.00E-70	2662	421	COG3469
