# Supplementary material for: In silico identification of coffee genome expressed sequences potentially associated with resistance to diseases
Source: Genet Mol Biol. 2010 Dec 1;33(4):795–806. doi: 10.1590/s1415-47572010000400031 (PMC3036153; doi:10.1590/s1415-47572010000400031)
Supplement: Table S6 — EST-contigs with E-values < e-20 and scores > 100 obtained in the project Cytochrome P450, and their blast hits, scores, E-values, sizes, number of reads and conserved domains from putative proteins [file gmb-33-4-795-suppl6.pdf]

Table S6: EST-Contigs with e-value < e^{-20} and score > 100 obtained in the Project Cytochrome P450, and their blast hit, score, e-value, size, number of reads, and conserved domains from putative proteins.

Cytochrome P450						
Contig	BLAST NR	Score	e-value	Length	Reads	Conserved Domains
1	gi|147857238|emb|CAN83490.1| hypothetical protein [Vitis vinifera]	394	0	1087	2	pfam00067
2	gi|147860148|emb|CAN78724.1| hypothetical protein [Vitis vinifera]	204	3.00E-51	564	2	pfam00067
3	gi|15229044|ref|NP_190458.1| ATMP2 [Arabidopsis thaliana]	169	1.00E-40	663	3	pfam00173
4	gi|147786937|emb|CAN60080.1| hypothetical protein [Vitis vinifera]	292	2.00E-77	974	3	pfam00067
5	gi|147812439|emb|CAN65790.1| hypothetical protein [Vitis vinifera]	249	1.00E-64	806	5	pfam00067
7	gi|147864286|emb|CAN83013.1| hypothetical protein [Vitis vinifera]	167	4.00E-40	613	6	pfam00067
8	gi|125574606|gb|EAZ15890.1| hypothetical protein OsJ_030099 [Oryza sativa]	208	3.00E-52	813	2	COG2124
9	gi|147777974|emb|CAN74205.1| hypothetical protein [Vitis vinifera]	376	0	1120	4	pfam00067
10	gi|85068668|gb|ABC69414.1| CYP72A57 [Nicotiana tabacum]	400	0	1081	4	pfam00067
11	gi|92893043|gb|ABE91557.1| E-class P450, group I [Medicago truncatula]	446	0	1487	15	pfam00067
12	gi|21536603|gb|AAM60935.1| unknown [Arabidopsis thaliana]	354	4.00E-96	1033	3	No CD has been identified
13	gi|147801850|emb|CAN75347.1| hypothetical protein [Vitis vinifera]	488	0	878	4	pfam00067
14	gi|84514175|gb|ABC59096.1| cytochrome P450 monooxygenase CYP97C10 [Medicago truncatula].	272	7.00E-98	908	3	pfam00067
16	gi|2695711|emb|CAA04703.1| cytochome b5 [Olea europaea]	126	1.00E-27	740	3	pfam00173
17	gi|85068594|gb|ABC69377.1| CYP81B2v2 [Nicotiana tabacum]	295	2.00E-78	688	4	pfam00067
18	gi|147864456|emb|CAN80496.1| hypothetical protein [Vitis vinifera]	282	2.00E-74	904	2	pfam00067
19	gi|63029720|gb|AAY27751.1| allene oxide synthase [Hevea brasiliensis]	275	3.00E-72	935	5	pfam00067
20	gi|147858518|emb|CAN81014.1| hypothetical protein [Vitis vinifera]	171	7.00E-44	739	3	pfam00067
21	gi|18420515|ref|NP_568068.1| electron transport SCO1/SenC family protein [Arabidopsis thaliana]	261	7.00E-68	1156	3	cd02968
22	gi|147784145|emb|CAN72302.1| hypothetical protein [Vitis vinifera]	744	0	2395	14	pfam00067
23	gi|92870560|gb|ABE79844.1| E-class P450, group I [Medicago truncatula]	191	3.00E-47	664	2	pfam00067
24	gi|12321297|gb|AAG50718.1|AC079041_11 cytochrome P450, putative [Arabidopsis thaliana]	844	0	1757	5	pfam00067
25	gi|77461113|ref|YP_350620.1| Cytochrome bd ubiquinol oxidase, subunit II [Pseudomonas fluorescens PfO-1]	226	9.00E-58	848	13	pfam02322, COG1294
26	gi|126445639|ref|YP_001079231.1| bacterioferritin [Burkholderia mallei NCTC 10247]	280	6.00E-74	794	2	cd00907
27	gi|27764531|gb|AAO23063.1| ent-kaurenoic acid oxidase [Pisum sativum]	266	6.00E-70	751	3	COG1294
28	gi|85068674|gb|ABC69417.1| CYP72A54 [Nicotiana tabacum]	723	0	1653	7	pfam00067
29	gi|33603796|ref|NP_891356.1| putative cytochrome oxidase assembly protein [Bordetella bronchiseptica RB50]	268	2.00E-70	745	2	COG1612
30	gi|115453349|ref|NP_001050275.1| Os03g0390400 [Oryza sativa]	156	7.00E-37	623	3	cd00926
31	gi|110433184|gb|ABG74350.1| cytochrome P450 [Capsicum chinense]	714	0	1639	14	pfam00067
32	gi|145337333|ref|NP_177109.3| oxygen binding [Arabidopsis thaliana]	529	0	1057	9	pfam00067
33	gi|18203445|sp|Q9SPB3|RL10_VITRI 60S ribosomal protein L10 (QM protein homolog) [Vitis riparia]	443	0	969	40	PRK04199
34	gi|147852119|emb|CAN80156.1| hypothetical protein [Vitis vinifera]	360	7.00E-98	1030	2	pfam00067
35	gi|50346814|ref|YP_053185.1| cytochrome b6/f complex subunit IV [Nymphaea alba]	327	6.00E-88	957	5	cd00290
36	gi|15235720|ref|NP_195496.1| cytochrome c oxidase-related [Arabidopsis thaliana]	125	2.00E-27	657	8	cd00925
37	gi|121998106|ref|YP_001002893.1| cytochrome B561 [Halorhodospira halophila SL1]	87	6.00E-22	722	2	COG3658
38	gi|147838607|emb|CAN69513.1| hypothetical protein [Vitis vinifera]	239	1.00E-61	738	2	pfam00067
39	gi|5915836|sp|P93531|C71D7_SOLCH Cytochrome P450 71D7 [Solanum chacoense]	228	3.00E-58	681	3	pfam00067
40	gi|85068684|gb|ABC69422.1| CYP72A58 [Nicotiana tabacum]	674	0	1740	4	pfam00067
41	gi|115437178|ref|NP_001043230.1| Os01g0527700 [Oryza sativa]	490	0	1559	7	COG0109
42	gi|85068674|gb|ABC69417.1| CYP72A54 [Nicotiana tabacum]	490	0	2252	7	pfam00067
44	gi|85001719|gb|ABC68413.1| cytochrome P450 monooxygenase CYP76E3 [Glycine max]	238	4.00E-61	948	3	pfam00067
45	gi|115480934|ref|NP_001064060.1| Os10g0118800 [Oryza sativa]	159	5.00E-37	1405	4	pfam03188
46	gi|92867559|gb|ABE77793.1| E-class P450, group I [Medicago truncatula]	339	5.00E-94	945	6	pfam00067
47	gi|1345882|sp|P49098|CYB5_TOBAC Cytochrome b5 [Nicotiana tabacum]	242	2.00E-62	1000	11	pfam00173
48	gi|147777491|emb|CAN62730.1| hypothetical protein [Vitis vinifera]	304	4.00E-81	818	9	pfam00067
49	gi|85068656|gb|ABC69408.1| CYP71AT2v1 [Nicotiana tabacum]	303	3.00E-81	1182	8	pfam00067
51	gi|33601781|ref|NP_889341.1| Putative heme export protein [Bordetella bronchiseptica RB50]	281	1.00E-74	508	3	COG0755
52	gi|18252325|gb|AAL66194.1|AF386512_1 cytochrome P450 [Pyrus communis]	221	3.00E-56	897	2	pfam00067
53	gi|147812439|emb|CAN65790.1| hypothetical protein [Vitis vinifera]	718	0	1820	17	pfam00067
54	gi|18394631|ref|NP_564058.1| rhomboid family protein [Arabidopsis thaliana]	180	1.00E-43	879	3	pfam01694
55	gi|115466706|ref|NP_001056952.1| Os06g0175900 [Oryza sativa]	122	9.00E-27	595	6	No CD has been identified
56	gi|147778583|emb|CAN60309.1| hypothetical protein [Vitis vinifera]	283	1.00E-74	797	5	pfam00067
57	gi|147782531|emb|CAN68429.1| hypothetical protein [Vitis vinifera]	171	3.00E-41	734	2	pfam00067
58	gi|147794657|emb|CAN73509.1| hypothetical protein [Vitis vinifera]	146	1.00E-33	733	2	pfam00067
59	gi|33593694|ref|NP_881338.1| cytochrome ubiquinol oxidase subunit I [Bordetella pertussis Tohama I]	305	6.00E-82	538	4	cd01662, COG0843
60	gi|139538863|gb|ABO77958.1| p-coumaroyl quinate/shikimate 3'-hydroxylase [Coffea canephora]	1034	0	1877	17	pfam00067
61	gi|18252325|gb|AAL66194.1|AF386512_1 cytochrome P450 [Pyrus communis]	162	1.00E-38	588	2	pfam00067
63	gi|1351206|sp|P48522|TCMO_CATRO Trans-cinnamate 4-monooxygenase [Catharanthus roseus]	490	0	1011	12	pfam00067
64	gi|77457812|ref|YP_347317.1| Cytochrome c-type biogenesis protein [Pseudomonas fluorescens PfO-1]	346	9.00E-94	810	10	cd00189, COG4235
65	gi|130270|sp|P00290|PLAS_LACSA Plastocyanin [Lactuca sativa]	177	2.00E-43	582	3	pfam00127
66	gi|147821410|emb|CAN63505.1| hypothetical protein [Vitis vinifera]	135	2.00E-30	710	2	pfam00067
67	gi|71897452|gb|AAZ52550.1| cytochrome P450 [Capsicum annuum]	288	2.00E-76	980	4	pfam00067
68	gi|147833535|emb|CAN68303.1| hypothetical protein [Vitis vinifera]	333	7.00E-90	763	9	pfam00067
69	gi|77461691|ref|YP_351198.1| Cytochrome c, class I [Pseudomonas fluorescens]	161	1.00E-38	491	21	COG0672, COG2010
70	gi|70730201|ref|YP_259940.1| cytochrome c oxidase, cbb3-type, subunit I [Pseudomonas	441	0	649	2	cd01661

	fluorescens Pf-5]					
71	gi|129593742|gb|ABO31111.1| sphingolipid delta-8 desaturase [Nicotiana tabacum]	798	0	1737	9	cd03506, pfam00173, cd03507
73	gi|147844260|emb|CAN80040.1| hypothetical protein [Vitis vinifera]	697	0	1486	5	pfam00067
75	gi|2695711|emb|CAA04703.1| cytochome b5 [Olea europaea]	244	5.00E-63	830	14	pfam00173
76	gi|147855053|emb|CAN82364.1| hypothetical protein [Vitis vinifera]	159	2.00E-37	722	7	pfam00067
77	gi|147866210|emb|CAN79423.1| hypothetical protein [Vitis vinifera]	313	4.00E-84	686	2	pfam00067
78	gi|18203445|sp|Q9SPB3|RL10_VITRI 60S ribosomal protein L10 (QM protein homolog) [Vitis riparia]	442	0	964	25	PRK04199
79	gi|147777975|emb|CAN74206.1| hypothetical protein [Vitis vinifera]	274	3.00E-72	695	3	pfam00067
80	gi|1351357|sp|P48502|UCR6_SOLTU Ubiquinol-cytochrome c reductase complex 14 kDa protein (CR14) [Solanum tuberosum]	155	3.00E-36	923	17	pfam02271
81	gi|85068668|gb|ABC69414.1| CYP72A57 [Nicotiana tabacum]	328	3.00E-88	873	5	pfam00067
82	gi|30678524|ref|NP_186877.2| flavodoxin family protein [Arabidopsis thaliana]	498	0	1134	4	pfam00258, pfam00175, COG0369
83	gi|147841765|emb|CAN62209.1| hypothetical protein [Vitis vinifera]	205	4.00E-51	1388	31	pfam00067
87	gi|1040729|emb|CAA60621.1| delta-8 sphingolipid desaturase [Helianthus annuus]	694	0	1910	7	cd03506, pfam00173, cd03511
85	gi|147776011|emb|CAN71371.1| hypothetical protein [Vitis vinifera]	290	5.00E-77	739	5	pfam00067
86	gi|147767047|emb|CAN67678.1| hypothetical protein [Vitis vinifera]	555	0	2037	16	pfam00067
141	gi|115484331|ref|NP_001065827.1| Os11g0162200 [Oryza sativa]	115	3.00E-24	796	6	pfam02320
142	gi|147795107|emb|CAN60851.1| hypothetical protein [Vitis vinifera]	286	1.00E-75	914	3	pfam00067
143	gi|1351357|sp|P48502|UCR6_SOLTU Ubiquinol-cytochrome c reductase complex 14 kDa protein (CR14) [Solanum tuberosum]	152	2.00E-35	840	6	pfam02271
144	gi|92872915|gb|ABE81444.1| E-class P450, group I [Medicago truncatula]	278	2.00E-73	819	4	pfam00067
145	gi|66044163|ref|YP_234004.1| FMN-dependent alpha-hydroxy acid dehydrogenase [Pseudomonas syringae pv. syringae B728a]	223	2.00E-57	373	9	cd02809, PRK11197
146	gi|77460775|ref|YP_350282.1| Periplasmic Sensor Signal Transduction Histidine Kinase [Pseudomonas fluorescens PfO-1]	305	2.00E-81	829	8	cd00075, COG4191
147	gi|15241121|ref|NP_200420.1| COX15 (CYTOCHROME C OXIDASE 15) [Arabidopsis thaliana]	137	6.00E-31	868	2	COG1612
149	gi|18420859|ref|NP_568463.1| CYP714A1 (cytochrome P450, family 714, subfamily A, polypeptide 1); oxygen binding [Arabidopsis thaliana]	236	8.00E-61	680	3	pfam00067
150	gi|147821972|emb|CAN77159.1| hypothetical protein [Vitis vinifera]	283	0	1738	8	pfam00067
151	gi|147811196|emb|CAN70159.1| hypothetical protein [Vitis vinifera]	269	2.00E-73	851	3	pfam00067
152	gi|22330642|ref|NP_177656.2| flavodoxin family protein / radical SAM domain-containing protein [Arabidopsis thaliana]	204	3.00E-51	676	2	pfam08608, pfam00258, pfam04055, COG0731
153	gi|124484371|dbj|BAF46296.1| cytochrome P450 [Ipomoea nil]	285	2.00E-75	749	3	pfam00067
154	gi|147840876|emb|CAN73186.1| hypothetical protein [Vitis vinifera]	293	2.00E-77	1260	20	pfam00067
155	gi|145323848|ref|NP_001077513.1| unknown protein [Arabidopsis thaliana]	121	2.00E-26	565	3	No CD has been identified
156	gi|147765656|emb|CAN71501.1| hypothetical protein [Vitis vinifera]	727	0	2096	15	pfam00067
157	gi|1619602|emb|CAA69976.1| MtN3 [Medicago truncatula]	237	5.00E-61	731	2	pfam03083
158	gi|92885617|gb|ABE87844.1| E-class P450, group I [Medicago truncatula]	265	1.00E-85	1055	2	pfam00067
160	gi|147786939|emb|CAN60082.1| hypothetical protein [Vitis vinifera]	264	4.00E-69	745	3	pfam00067
161	gi|85068648|gb|ABC69404.1| CYP71D51v2 [Nicotiana tabacum]	437	0	1575	4	pfam00067
162	gi|46403211|gb|AAS92625.1| coniferylalcohol 5-hydroxylase [Centaurium erythraea]	353	5.00E-96	781	2	pfam00067
163	gi|147843271|emb|CAN80536.1| hypothetical protein [Vitis vinifera]	509	0	1420	5	pfam00067
164	gi|147835182|emb|CAN76753.1| hypothetical protein [Vitis vinifera]	503	0	1213	5	pfam00067
165	gi|48428165|sp|Q9LZQ0|CX5C2_ARATH Cytochrome c oxidase polypeptide Vc-2 (Cytochrome c oxidase subunit 5c-2) [Arabidopsis thaliana]	107	5.00E-22	591	2	pfam05799
166	gi|5915840|sp|O22307|C71DB_LOTJA Cytochrome P450 71D11 [Lotus japonicus]	230	6.00E-59	688	2	pfam00067
167	gi|77459567|ref|YP_349074.1| Transcriptional Regulator, Crp/Fnr family [Pseudomonas fluorescens PfO-1]	255	9.00E-67	603	2	cd00038, cd00092, PRK11161
168	gi|82570227|gb|ABB83676.1| putative p-coumaroyl 3'-hydroxylase CYP98A-C1 [Coffea canephora]	503	0	915	6	pfam00067
169	gi|12331298|emb|CAC24711.1| cytochrome P450 [Solanum tuberosum]	546	0	1655	20	pfam00067
170	gi|37954114|gb|AAP69988.1| ent-kaurene oxidase [Pisum sativum]	592	0	2462	30	pfam00067
171	gi|115446391|ref|NP_001046975.1| Os02g0520800 [Oryza sativa]	272	2.00E-71	773	4	cd03470
173	gi|587562|emb|CAA56520.1| mitochondrial processing peptidase [Solanum tuberosum]	273	1.00E-71	887	5	pfam00675, pfam05193, COG0612
174	gi|147765747|emb|CAN60189.1| hypothetical protein [Vitis vinifera]	356	2.00E-96	1104	4	pfam00067
175	gi|15241121|ref|NP_200420.1| COX15 (CYTOCHROME C OXIDASE 15) [Arabidopsis thaliana]	221	1.00E-61	671	3	COG1612
176	gi|139538863|gb|ABO77958.1| p-coumaroyl quinate/shikimate 3'-hydroxylase [Coffea canephora]	390	0	894	3	pfam00067
177	gi|147772136|emb|CAN75686.1| hypothetical protein [Vitis vinifera]	189	9.00E-47	697	2	pfam00067
178	gi|15982240|emb|CAC91565.1| hydroperoxide lyase [Nicotiana attenuata]	298	1.00E-79	727	2	pfam00067
179	gi|27764531|gb|AAO23063.1| ent-kaurenoic acid oxidase [Pisum sativum]	321	3.00E-86	805	3	COG2124
180	gi|85068684|gb|ABC69422.1| CYP72A58 [Nicotiana tabacum]	660	0	1546	11	pfam00067
181	gi|147774515|emb|CAN76784.1| hypothetical protein [Vitis vinifera]	225	2.00E-57	720	2	pfam00067
182	gi|147795107|emb|CAN60851.1| hypothetical protein [Vitis vinifera]	139	2.00E-31	829	2	pfam00067
183	gi|126355969|ref|ZP_01712975.1| Amine dehydrogenase [Pseudomonas putida]	267	4.00E-70	787	8	pfam06433
184	gi|77460158|ref|YP_349665.1| thiol:disulfide interchange protein DsbE, putative [Pseudomonas fluorescens PfO-1]	246	2.00E-64	458	13	cd02966, pfam01790
185	gi|147842082|emb|CAN62647.1| hypothetical protein [Vitis vinifera]	200	8.00E-50	781	3	pfam00067
186	gi|71081902|gb|AAZ23260.1| cytochrome P450 monooxygenase [Nicotiana tabacum]	115	1.00E-24	595	3	COG2124
187	gi|118022|sp|P00054|CYC_SESIN Cytochrome c [Sesamum indicum]	220	1.00E-55	984	18	COG3474
188	gi|85068648|gb|ABC69404.1| CYP71D51v2 [Nicotiana tabacum]	291	6.00E-77	1163	2	pfam00067
189	gi|27754697|gb|AAO22792.1| putative cytochrome c oxidoreductase [Arabidopsis thaliana]	268	2.00E-70	792	6	pfam03083
191	gi|119897307|ref|YP_932520.1| L-lactate dehydrogenase [Azoarcus sp. BH72]	316	7.00E-85	742	2	cd03332, pfam01070
192	gi|26106071|dbj|BAC41516.1| NADPH-cytochrome P-450 reductase [Ophiorrhiza pumila]	1078	0	2692	22	pfam00258, pfam00175, COG0369
193	gi|85068672|gb|ABC69416.1| CYP71AU1 [Nicotiana tabacum]	422	0	1186	3	pfam00067
194	gi|63029720|gb|AAY27751.1| allene oxide synthase [Hevea brasiliensis]	657	0	2020	31	pfam00067
195	gi|147858518|emb|CAN81014.1| hypothetical protein [Vitis vinifera]	506	0	1851	14	pfam00067
196	gi|15219780|ref|NP_176265.1| B5 #5 (cytochrome b5 family protein #5) [Arabidopsis thaliana]	143	1.00E-32	862	2	pfam00173
197	gi|147794492|emb|CAN62759.1| hypothetical protein [Vitis vinifera]	115	1.00E-24	620	7	pfam00067

198	gi|27529728|dbj|BAC53893.1| cytochrome P450 [Petunia x hybrida]	298	0	1645	6	pfam00067
199	gi|157357203|emb|CAO63697.1|[157357203] unnamed protein product [Vitis vinifera]	537	1.00E-150	1704	50	pfam00067
204	gi|115265663|dbj|BAF32924.1| cytochrome bd ubiquinol oxidase, subunit II [Pseudomonas syringae pv. phaseolicola]	228	8.00E-59	520	5	pfam02322, COG1294
211	gi|147801862|emb|CAN74977.1| hypothetical protein [Vitis vinifera]	110	5.00E-23	565	2	pfam00067
220	gi|84578869|dbj|BAE72877.1|[84578869] cytochrome P450 [Verbena x hybrida]	231	3.00E-61	781	2	pfam00067
221	gi|14423327|gb|AAK62346.1|[14423327] elicitor-inducible cytochrome P450 [Nicotiana tabacum]	353	1.00E-100	857	3	pfam00067
225	gi|147769656|emb|CAN74644.1|[147769656] hypothetical protein [Vitis vinifera]	295	2.00E-78	740	2	pfam00067
227	gi|147742913|sp|P84887|AAUA_ALCFA Aralkylamine dehydrogenase light chain precursor (Aromatic amine dehydrogenase) [Alcaligenes faecalis]	215	2.00E-54	681	2	pfam02975
231	gi|147828267|emb|CAN75404.1|[147828267] hypothetical protein [Vitis vinifera]	238	4.00E-68	883	5	pfam00067
232	gi|85068612|gb|ABC69386.1|[85068612] CYP92B2v1 [Nicotiana tabacum]	236	6.00E-61	690	2	pfam00067
234	gi|17065916|emb|CAC80883.1| geraniol 10-hydroxylase [Catharanthus roseus]	172	6.00E-64	595	2	pfam00067
235	gi|125580889|gb|EAZ21820.1|[125580889] hypothetical protein OsJ_005303 [Oryza sativa]	299	1.00E-79	780	2	cd02968
