# Supplementary material for: In silico identification of coffee genome expressed sequences potentially associated with resistance to diseases
Source: Genet Mol Biol. 2010 Dec 1;33(4):795–806. doi: 10.1590/s1415-47572010000400031 (PMC3036153; doi:10.1590/s1415-47572010000400031)
Supplement: Table S7 — EST-contigs with E-values < e-20 and scores > 100 obtained in the project Glucanase, and their blast hits, scores, E-values, sizes, number of reads and conserved domains from putative proteins. [file gmb-33-4-795-suppl7.pdf]

Table S7: EST-Contigs with e-value < e^{-20} and score > 100 obtained in the Project Glucanase, and their blast hit, score, e-value, size, number of reads, and conserved domains from putative proteins.

Glucanase						
Contig	BLAST NR	Score	e-value	Length	Reads	Conserved Domains
1	gi|91107165|gb|ABE11608.1| xyloglucan endo-transglycosylase precursor [Solanum chacoense]	212	1.00E-53	665	11	cd02176, pfam06955
2	gi|92868701|gb|ABE78694.1| Glycoside hydrolase, family 17; Virulence factor, pectin lyase fold [Medicago truncatula]	203	5.00E-51	774	3	pfam07983, pfam00332
3	gi|94442926|emb|CAJ91137.1| beta-1,3-glucanase [Platanus x acerifolia]	226	6.00E-58	802	2	pfam00332
4	gi|34909360|ref|NP_916027.1| P0638D12.12 [Oryza sativa (japonica cultivar-group)]	213	1.00E-62	848	4	pfam00332
5	gi|92895371|gb|ABE92687.1| Glycoside transferase, six-hairpin, subgroup [Medicago truncatula]	578	0	1493	3	pfam00759
6	gi|62362434|gb|AAX81588.1| nectarin IV [Nicotiana langsdorffii x Nicotiana sanderae]	392	0	1707	11	No CD has been identified
7	gi|29826242|gb|AAO91861.1| TGB12K interacting protein 2 [Nicotiana tabacum]	311	3.00E-83	1054	13	cd00204
8	gi|3582436|dbj|BAA33065.1| beta-D-glucan exohydrolase [Nicotiana tabacum]	324	5.00E-87	1330	5	COG1472, pfam01915
9	gi|14279169|gb|AAK58515.1| beta-1,3-glucanase-like protein [Olea europaea]	296	2.00E-78	999	2	pfam07983, pfam00332
10	gi|4662638|gb|AAD26909.1| putative beta-1,3-glucanase [Arabidopsis thaliana]	296	1.00E-79	839	6	pfam07983, pfam00332
11	gi|91107165|gb|ABE11608.1| xyloglucan endo-transglycosylase precursor [Solanum chacoense]	483	0	1081	14	cd02176, pfam06955
12	gi|34329342|gb|AAQ63883.1| cellulase [Medicago truncatula]	158	5.00E-37	1372	3	pfam00759
13	gi|33324614|gb|AAQ08018.1| endo-1,4-beta-glucanase [Gossypium hirsutum]	530	0	1111	3	pfam00759
14	gi|56785404|dbj|BAD82640.1| putative elicitor inducible beta-1,3-glucanase NtEIG-E76 [Oryza sativa (japonica cultivar-group)]	321	3.00E-86	839	3	pfam00332
15	gi|9294513|dbj|BAB02775.1| unnamed protein product [Arabidopsis thaliana]	156	1.00E-36	1079	3	COG0412
16	gi|15228047|ref|NP_181224.1| hydrolase, acting on glycosyl bonds / hydrolase, hydrolyzing O-glycosyl compounds [Arabidopsis thaliana]	115	6.00E-48	798	6	cd02176, pfam06955
17	gi|2244740|dbj|BAA21111.1| endo-1,4-beta-glucanase [Gossypium hirsutum]	385	0	1377	11	pfam00759
18	gi|55740509|gb|AAV63847.1| hypothetical protein At1g29380 [Arabidopsis thaliana]	141	3.00E-32	811	2	pfam07983
19	gi|8886865|gb|AAF80590.1| xyloglucan endotransglycosylase XET1 [Asparagus officinalis]	134	2.00E-62	656	2	cd02176, pfam06955
20	gi|55978791|gb|AAV68857.1| hypothetical protein AT1G79480 [Arabidopsis thaliana]	135	3.00E-30	876	2	pfam07983
21	gi|15232590|ref|NP_190241.1| hydrolase, hydrolyzing O-glycosyl compounds [Arabidopsis thaliana]	203	5.00E-51	717	2	cd02176, pfam06955
22	gi|50924081|ref|XP_472401.1| OSJNBa0073L04.8 [Oryza sativa (japonica cultivar-group)]	275	0	1392	2	pfam00332
23	gi|62362438|gb|AAX81590.1| beta-1,3-glucanase [Fragaria x ananassa]	377	0	1469	5	pfam00332
24	gi|11071974|dbj|BAB17320.1| elicitor inducible beta-1,3-glucanase NtEIG-E76 [Nicotiana tabacum]	682	0	1783	11	pfam07983, pfam00332
25	gi|15225764|ref|NP_180858.1| hydrolase, hydrolyzing O-glycosyl compounds [Arabidopsis thaliana]	298	2.00E-79	955	2	cd02176, pfam06955
26	gi|57899486|dbj|BAD86947.1| putative elicitor inducible beta-1,3-glucanase NtEIG-E76 [Oryza sativa (japonica cultivar-group)]	342	9.00E-93	759	2	pfam07983, pfam00332
27	gi|94442926|emb|CAJ91137.1| beta-1,3-glucanase [Platanus x acerifolia]	278	1.00E-73	797	4	pfam00332
28	gi|13560781|gb|AAK30204.1| endoxyloglucan transferase [Daucus carota]	507	0	1282	4	cd02176, pfam06955
29	gi|1885310|emb|CAA62847.1| Endoxyloglucan transferase (EXT) [Hordeum vulgare subsp. vulgare]	140	2.00E-63	1365	4	cd02176, pfam06955
30	gi|33391721|gb|AAQ17461.1| beta-D-glucosidase [Gossypium hirsutum]	591	0	1274	6	COG1472, pfam01915
31	gi|14029149|gb|AAK51119.1| xyloglucan endo-transglycosylase [Carica papaya]	397	0	869	4	cd02176, pfam06955
32	gi|18391291|ref|NP_563892.1| hydrolase, acting on glycosyl bonds / hydrolyzing O-glycosyl compounds [Arabidopsis thaliana]	308	0	885	6	cd02176, pfam06955
33	gi|70927645|gb|AAZ15705.1| endo-alpha-1,4-glucanase [Gossypium hirsutum]	914	0	2119	12	COG1472, pfam01915
34	gi|19911573|dbj|BAB86890.1| syringolide-induced protein 19-1-5 [Glycine max]	290	9.00E-92	747	2	cd02176, pfam06955
35	gi|19911573|dbj|BAB86890.1| syringolide-induced protein 19-1-5 [Glycine max]	466	0	1173	13	cd02176, pfam06955
36	gi|92891730|gb|ABE90942.1| putative glycosyl hydrolase family 17 protein [Medicago truncatula]	163	1.00E-38	951	10	pfam07983
37	gi|90186655|gb|ABD91577.1| beta-1,3-glucanase [Medicago sativa]	218	1.00E-55	707	2	pfam00332
38	gi|18403812|ref|NP_566731.1| hydrolase [Arabidopsis thaliana]	229	2.00E-58	940	5	COG0412
39	gi|37223500|gb|AAQ90287.1| beta-1,3-glucanase, acidic [Coffea arabica]	253	9.00E-66	819	2	pfam07983, pfam00332
40	gi|34908492|ref|NP_915593.1| putative beta-1,3-glucanase [Oryza sativa]	481	0	1549	13	pfam00332
41	gi|37223498|gb|AAQ90286.1| beta-1,3-glucanase, basic [Coffea arabica]	585	0	1412	19	pfam00332
43	gi|92885855|gb|ABE87944.1| Glycosyl hydrolases family 17 [Medicago truncatula]	281	1.00E-74	749	3	pfam07983, pfam00332
44	gi|15235714|ref|NP_195494.1| hydrolase, acting on glycosyl bonds / hydrolyzing O-glycosyl compounds [Arabidopsis thaliana]	101	1.00E-39	1255	5	cd02176, pfam06955
45	gi|90186653|gb|ABD91576.1| beta-1,3-glucanase [Medicago sativa]	193	4.00E-48	656	3	pfam00332
47	gi|29243202|dbj|BAC66186.1| beta-1,3-glucanase [Fragaria x ananassa]	316	1.00E-84	976	4	pfam00332
48	gi|2065531|gb|AAC49704.1| endo-1,4-beta-glucanase [Lycopersicon esculentum]	368	0	1062	5	pfam00759
49	gi|42795460|gb|AAS46240.1| xyloglucan endotransglucosylase-hydrolase XTH5 [Lycopersicon esculentum]	487	0	1300	16	cd02176, pfam06955
50	gi|108710308|gb|ABF98103.1| Glucan endo-1,3-beta-glucosidase 7 precursor, putative, expressed [Oryza sativa (japonica cultivar-group)]	348	4.00E-94	1665	6	pfam07983, pfam00332
51	gi|42795462|gb|AAS46241.1| xyloglucan endotransglucosylase-hydrolase XTH3 [Lycopersicon esculentum]	389	0	1136	12	cd02176, pfam06955
52	gi|285741|dbj|BAA03413.1| EDGP precursor [Daucus carota]	584	0	1567	12	No CD has been identified
53	gi|2290683|gb|AAB65156.1| basic cellulase [Citrus sinensis]	401	0	888	3	pfam00759
54	gi|37223498|gb|AAQ90286.1| beta-1,3-glucanase, basic [Coffea arabica]	641	0	1249	13	pfam00332
55	gi|3582436|dbj|BAA33065.1| beta-D-glucan exohydrolase [Nicotiana tabacum]	285	1.00E-83	636	2	COG1472, pfam01915
56	gi|30678225|ref|NP_178637.2| hydrolase, hydrolyzing O-glycosyl compounds [Arabidopsis thaliana]	308	1.00E-82	697	2	cd02176, pfam06955
57	gi|37223500|gb|AAQ90287.1| beta-1,3-glucanase, acidic [Coffea arabica]	428	0	1425	3	pfam07983, pfam00332
58	gi|15232711|ref|NP_190288.1| hydrolase, hydrolyzing O-glycosyl compounds [Arabidopsis thaliana]	291	3.00E-77	1062	4	cd02176, pfam06955
59	gi|92872472|gb|ABE81087.1| Glycoside transferase, six-hairpin, subgroup [Medicago truncatula]	254	2.00E-66	742	2	pfam00759
60	gi|51854423|gb|AAU10802.1| 'putative endo-1,3;1,4-beta-D-glucanase' [Oryza sativa (japonica cultivar-group)]	232	2.00E-59	1078	5	COG0412
61	gi|70780051|gb|AAZ08349.1| xyloglucan endotransglycosylase/hydrolase XTH5 [Lycopersicon esculentum]	448	0	1276	16	cd02176, pfam06955
62	gi|30690053|ref|NP_195174.3| hydrolase, hydrolyzing O-glycosyl compounds [Arabidopsis thaliana]	304	3.00E-81	981	4	cd02176, pfam06955

63	gi|37223500|gb|AAQ90287.1| beta-1,3-glucanase, acidic [Coffea arabica]	171	7.00E-42	449	5	pfam07983, pfam00332
64	gi|18394637|ref|NP_564059.1| unknown protein [Arabidopsis thaliana]	171	3.00E-41	720	2	pfam07983
65	gi|4165132|gb|AAD08699.1| endo-beta-1,4-D-glucanase [Lycopersicon esculentum]	139	4.00E-41	1020	28	pfam00759
66	gi|11071974|dbj|BAB17320.1| elicitor inducible beta-1,3-glucanase NtEIG-E76 [Nicotiana tabacum]	320	4.00E-86	802	4	pfam07983, pfam00332
67	gi|37625031|gb|AAQ96339.1| putative ankyrin-repeat protein [Vitis aestivalis]	248	3.00E-64	866	2	cd00204
68	gi|15232707|ref|NP_190284.1| hydrolase, hydrolyzing O-glycosyl compounds [Arabidopsis thaliana]	491	0	1033	3	cd02176, pfam06955
69	gi|79328626|ref|NP_001031936.1| hydrolase, hydrolyzing O-glycosyl com.pounds [Arabidopsis thaliana]	215	1.00E-68	971	5	cd02176, pfam06955
70	gi|92891730|gb|ABE90942.1| putative glycosyl hydrolase family 17 protein [Medicago truncatula]	161	3.00E-38	916	5	pfam07983
71	gi|577068|emb|CAA58002.1| xyloglycan endo-transglycosylase [Lycopersicon esculentum]	237	1.00E-77	843	3	cd02176, pfam06955
72	gi|22947852|gb|AAN07898.1| xyloglucan endotransglycosylase [Malus x domestica]	360	4.00E-98	758	2	cd02176, pfam06955
73	gi|285741|dbj|BAA03413.1| EDGP precursor [Daucus carota]	299	9.00E-80	904	3	No CD has been identified
75	gi|42795466|gb|AAS46243.1| xyloglucan endotransglucosylase-hydrolase XTH5 [Lycopersicon esculentum]	466	0	1347	17	cd02176, pfam06955
76	gi|7414433|emb|CAB85903.1| beta-1,3 glucanase [Pisum sativum]	519	0	1725	15	pfam00332
77	gi|4165132|gb|AAD08699.1| endo-beta-1,4-D-glucanase [Lycopersicon esculentum]	248	6.00E-69	782	3	pfam00759
78	gi|2230955|emb|CAA72133.1| endo-1,4-beta-D-glucanase [Lycopersicon esculentum]	291	2.00E-77	734	2	pfam00759
79	gi|473102|emb|CAA82271.1| beta-1,3-glucanase [Nicotiana tabacum]	316	5.00E-85	743	2	pfam00332
81	gi|29500899|emb|CAD87533.1| putative xyloglucan endotransglycosylase - hydrolase XTH5 [Lycopersicon esculentum]	330	0	864	2	cd02176, pfam06955
82	gi|87240526|gb|ABD32384.1| Peptidase A1, pepsin [Medicago truncatula]	351	5.00E-95	1393	8	pfam00026
83	gi|70927645|gb|AAZ15705.1| endo-alpha-1,4-glucanase [Gossypium hirsutum]	430	0	1071	7	COG1472, pfam01915
84	gi|18403820|ref|NP_566732.1| hydrolase [Arabidopsis thaliana]	252	2.00E-65	1010	13	COG0412
85	gi|18403820|ref|NP_566732.1| hydrolase [Arabidopsis thaliana]	273	1.00E-71	1171	16	COG0412
86	gi|577068|emb|CAA58002.1| xyloglycan endo-transglycosylase [Lycopersicon esculentum]	379	0	891	2	cd02176, pfam06955
87	gi|4165132|gb|AAD08699.1| endo-beta-1,4-D-glucanase [Lycopersicon esculentum]	635	0	1333	8	pfam00759
88	gi|32482806|gb|AAP84703.1| putative xyloglucanase inhibitor [Solanum tuberosum]	540	0	1599	9	No CD has been identified
89	gi|16903351|gb|AAL30453.1| endo-beta-1,4-glucanase precursor [Nicotiana tabacum]	309	8.00E-83	732	2	pfam00759
90	gi|22331064|ref|NP_566459.2| FUS9 (FUSCA 9); ubiquitin conjugating enzyme [Arabidopsis thaliana]	261	3.00E-68	996	4	cd00195
91	gi|29826242|gb|AAO91861.1| TGB12K interacting protein 2 [Nicotiana tabacum]	482	0	1516	29	cd00204
92	gi|50944207|ref|XP_481631.1| putative glycosyl hydrolase [Oryza sativa]	160	3.00E-38	613	2	pfam00332
