# Supplementary material for: In silico identification of coffee genome expressed sequences potentially associated with resistance to diseases
Source: Genet Mol Biol. 2010 Dec 1;33(4):795–806. doi: 10.1590/s1415-47572010000400031 (PMC3036153; doi:10.1590/s1415-47572010000400031)
Supplement: Table S8 — EST-contigs with E-values < e-20 and scores > 100 obtained in the project HSP (Heat Shock Protein), and their blast hits, scores, E-values, sizes, number of reads and conserved domains from putative proteins. [file gmb-33-4-795-suppl8.pdf]

Table S8: EST-Contigs with e-value < e^{-20} and score > 100 obtained in the Project HSP (Heat Shock Protein), and their blast hit, score, e-value, size, number of reads, and conserved domains from putative proteins.

HSP (Heat Shock Protein)						
Contig	BLAST NR	Score	e-value	Length	Reads	Conserved Domains
1	gi|19618|emb|CAA41547.1| heat shock protein [Medicago sativa]	128	2.00E-28	721	3	cd00298
2	gi|558606|emb|CAA50022.1| Nthsp18p [Nicotiana tabacum]	219	6.00E-56	781	4	cd00298
3	gi|37704443|gb|AAR01526.1| cytosolic class I small heat shock protein 3B [Nicotiana tabacum]	132	2.00E-30	416	3	cd00298
4	gi|15225187|ref|NP_180771.1| HSP70T-2; ATP binding [Arabidopsis thaliana]	354	2.00E-96	749	2	pfam00012
5	gi|4456758|emb|CAB36910.1| heat shock protein 17.4 [Quercus suber]	233	7.00E-60	782	4	cd00298, COG0071
6	gi|30687816|ref|NP_189160.3| ROF1 (ROTAMASE FKBP 1); FK506 binding/calmodulin binding/peptidyl-prolyl cis-trans isomerase [Arabidopsis thaliana]	818	0	1995	11	cd00189, pfam00254
7	gi|15148884|gb|AAK84869.1| small heat stress protein class CIII [Lycopersicon esculentum]	164	4.00E-39	891	5	cd00298
8	gi|18415982|ref|NP_567663.1| unknown protein [Arabidopsis thaliana]	285	3.00E-75	1627	40	cd00189, smart00727
9	gi|1143427|emb|CAA52149.1| heat shock protein 70 [Cucumis sativus]	1077	0	2608	20	pfam02491, PRK00290
10	gi|1143427|emb|CAA52149.1| heat shock protein 70 [Cucumis sativus]	830	0	1928	21	pfam02491, PRK00290
11	gi|15240308|ref|NP_198583.1| unknown protein [Arabidopsis thaliana]	143	4.00E-33	611	2	cd00298
12	gi|1143427|emb|CAA52149.1| heat shock protein 70 [Cucumis sativus]	375	0	1106	7	pfam02491, PRK00290
13	gi|710434|gb|AAB03097.1| Hsp22.3 [Glycine max]	159	3.00E-38	712	4	cd00298, COG0071
14	gi|76057837|emb|CAH55766.1| peptidyl prolyl cis-trans isomerase [Oryza sativa]	247	3.00E-64	806	2	pfam00254
15	gi|6969976|gb|AAF34134.1| high molecular weight heat shock protein [MMalus x domestica]	351	9.00E-96	640	2	pfam00012
16	gi|14581677|gb|AAK64512.1| Hsp70 interacting protein/thioredoxin chimera [Vitis labrusca]	324	2.00E-87	784	2	cd02947, cd00189
17	gi|5257560|gb|AAD41409.1| cytosolic class II low molecular weight heat shock protein [Prunus dulcis]	214	3.00E-54	776	5	cd00298
18	gi|5302797|emb|CAB46039.1| HSP like protein [Arabidopsis thaliana]	239	7.00E-62	832	3	pfam00012
19	gi|558606|emb|CAA50022.1| Nthsp18p [Nicotiana tabacum]	222	1.00E-56	817	18	cd00298
20	gi|462013|sp|P35016|ENPL_CATRO Endoplasmin homolog precursor (GRP94 homolog) [Catharanthus roseus]	581	0	1244	5	pfam00183, cd00075
21	gi|50923325|ref|XP_472023.1| OSJNBa0091C07.4 [Oryza sativa (japonica cultivar-group)]	518	0	1484	8	pfam00254, cd00189
22	gi|77556324|gb|ABA99120.1| AC009176 putative heat-shock protein [Oryza sativa]	362	9.00E-99	832	2	pfam00183, PRK05218
23	gi|558606|emb|CAA50022.1| Nthsp18p [Nicotiana tabacum]	205	9.00E-52	683	10	cd00298
24	gi|123538|sp|P04793|HSP13_SOYBN 17.5 kDa class I heat shock protein (HSP 17.5-M) [Glycine max]	243	4.00E-63	757	3	cd00298
25	gi|30172151|emb|CAD89783.1| peptidylprolyl cis-trans isomerase [Oryza sativa]	243	5.00E-63	883	4	pfam00254
27	gi|558606|emb|CAA50022.1| Nthsp18p [Nicotiana tabacum]	219	9.00E-56	846	11	cd00298
28	gi|77380807|gb|ABA72320.1| heat shock protein YegD [Pseudomonas fluorenscens]	418	0	749	3	PRK11678
29	gi|710434|gb|AAB03097.1| Hsp22.3 [Glycine max]	159	2.00E-38	670	2	cd00298, COG0071
30	gi|19618|emb|CAA41547.1| heat shock protein [Medicago sativa]	129	9.00E-29	796	2	cd00298
31	gi|51091143|dbj|BAD35839.1| putative cyclophilin-40 [Oryza sativa (japonica cultivar-group)]	207	2.00E-52	729	3	cd01926, smart00028, cd00189
