# Supplementary material for: In silico identification of coffee genome expressed sequences potentially associated with resistance to diseases
Source: Genet Mol Biol. 2010 Dec 1;33(4):795–806. doi: 10.1590/s1415-47572010000400031 (PMC3036153; doi:10.1590/s1415-47572010000400031)
Supplement: Table S9 — EST-contigs with E-values < e-20 and scores > 100 obtained in the project Thaumatin, and their blast hits, scores, E-values, sizes, number of reads and conserved domains from putative proteins. [file gmb-33-4-795-suppl9.pdf]

Table S9: EST-Contigs with e-value < e^{-20} and score > 100 obtained in the Project Thaumatin, and their blast hit, score, e-value, size, number of reads, and conserved domains from putative proteins.

Thaumatin						
Contig	BLAST NR	Score	e-value	Length	Reads	Conserved Domains
1	gi|21592749|gb|AAM64698.1| putative thaumatin-like protein [Arabidopsis thaliana]	325	2.00E-87	1592	4	smart00205
2	gi|21592749|gb|AAM64698.1| putative thaumatin-like protein [Arabidopsis thaliana]	304	3.00E-81	911	7	smart00205
3	gi|4586372|dbj|BAA74546.2| thaumatin-like protein SE39b [Nicotiana tabacum]	313	5.00E-84	1011	4	smart00205
4	gi|71057064|emb|CAI38795.2| thaumatin-like protein [Actinidia deliciosa]	400	0	1017	41	pfam00314
5	gi|53830843|gb|AAU95244.1| putative thaumatin-like protein [Solanum tuberosum]	371	0	975	56	pfam00314
6	gi|71057064|emb|CAI38795.2| thaumatin-like protein [Actinidia deliciosa]	404	0	892	15	pfam00314
7	gi|33329390|gb|AAQ10092.1| thaumatin-like protein [Vitis vinifera]	381	0	1125	117	pfam00314
8	gi|14290153|gb|AAK59278.1| thaumatin-like protein [Sambucus nigra]	368	0	880	21	pfam00314
9	gi|6273385|gb|AAF06347.1| SCUTL2 [Vitis vinifera]	228	5.00E-84	925	10	smart00205
10	gi|4586372|dbj|BAA74546.2| thaumatin-like protein SE39b [Nicotiana tabacum]	281	2.00E-79	1037	3	smart00205
11	gi|15221033|ref|NP_173261.1| thaumatin, putative [Arabidopsis thaliana]	399	0	1147	6	smart00205
12	gi|12324220|gb|AAG52086.1| thaumatin-like protein; 9376-10898 [Arabidopsis thaliana]	314	0	858	7	smart00205
13	gi|2501182|sp|Q41350|OLP1_LYCES Osmotin-like protein precursor [Lycopersicon esculentum]	413	0	1135	10	smart00205
14	gi|38603816|gb|AAR24653.1| At5g40020 [Arabidopsis thaliana]	148	3.00E-34	890	2	smart00205
15	gi|7270553|emb|CAB81510.1| thaumatin-like protein [Arabidopsis thaliana]	343	6.00E-93	870	4	smart00205
16	gi|12323299|gb|AAG51631.1| thaumatin-like protein; 12104-13574 [Arabidopsis thaliana]	261	2.00E-68	765	2	smart00205
