# Supplementary material for: In silico identification of coffee genome expressed sequences potentially associated with resistance to diseases
Source: Genet Mol Biol. 2010 Dec 1;33(4):795–806. doi: 10.1590/s1415-47572010000400031 (PMC3036153; doi:10.1590/s1415-47572010000400031)
Supplement: Table S10 — EST-contigs with E-values < e-20 and scores > 100 obtained in the project Phytoalexin, and their blast hits, scores, E-values, sizes, number of reads and conserved domains from putative proteins. [file gmb-33-4-795-suppl10.pdf]

**Table S10:** EST-Contigs with e-value < $e^{-20}$ and score > 100 obtained in the Project Phytoalexin, and their blast hit, score, e-value, size, number of reads, and conserved domains from putative proteins.

| Phytoalexin | | | | | | |
|---|---|---|---|---|---|---|
| Contig | BLAST NR | Score | e-value | Length | Reads | Conserved Domains |
| 1 | gi\|58826317\|gb\|AAW82883.1\| phytoalexin-deficient 4-1 protein [Solanum tuberosum] | 123 | 7.00E-27 | 759 | 2 | cd00519 |
| 2 | gi\|58826317\|gb\|AAW82883.1\| phytoalexin-deficient 4-1 protein [Solanum tuberosum] | 126 | 9.00E-28 | 683 | 2 | cd00519 |
| 3 | gi\|58826317\|gb\|AAW82883.1\| phytoalexin-deficient 4-1 protein [Solanum tuberosum] | 528 | 0 | 1507 | 5 | cd00519 |
