# Supplementary material for: In silico identification of coffee genome expressed sequences potentially associated with resistance to diseases
Source: Genet Mol Biol. 2010 Dec 1;33(4):795–806. doi: 10.1590/s1415-47572010000400031 (PMC3036153; doi:10.1590/s1415-47572010000400031)
Supplement: Table S11 — EST-contigs with E-values < e-20 and scores > 100 obtained in the project Chalconesynthase, and their blast hits, scores, E-values, sizes, number of reads and conserved domains from putative proteins. [file gmb-33-4-795-suppl11.pdf]

Table S11: EST-Contigs with e-value < e^{-20} and score > 100 obtained in the Project Chalconesynthase, and their blast hit, score, e-value, size, number of reads, and conserved domains from putative proteins.

Chalconesynthase						
Contig	BLAST NR	Score	e-value	Length	Reads	Conserved Domains
1	gi|50380153|gb|AAT76306.1| aldo-keto reductase [Fragaria x ananassa]	202	6.00E-51	657	2	pfam00248
2	gi|77381754|gb|ABA73267.1| 3-oxoacyl-(acyl-carrier-protein) synthase III, putative [Pseudomonas fluorescens PfO-1]	271	1.00E-71	759	3	cd00830, PRK09352
3	gi|1345787|sp|P48387|CHS2_CAMSI Chalcone synthase 2 (Naringenin-chalcone synthase 2) [Camellia sinensis]	691	0	1775	15	cd00831, COG3424
4	gi|1345787|sp|P48387|CHS2_CAMSI Chalcone synthase 2 (Naringenin-chalcone synthase 2) [Camellia sinensis]	660	0	1514	52	cd00831, COG3424
5	gi|1345787|sp|P48387|CHS2_CAMSI Chalcone synthase 2 (Naringenin-chalcone synthase 2) [Camellia sinensis]	713	0	1509	73	cd00831, COG3424
