# Supplementary material for: In silico identification of coffee genome expressed sequences potentially associated with resistance to diseases
Source: Genet Mol Biol. 2010 Dec 1;33(4):795–806. doi: 10.1590/s1415-47572010000400031 (PMC3036153; doi:10.1590/s1415-47572010000400031)
Supplement: Table S12 — EST-contigs with E-values < e-20 and scores > 100 obtained in the project Polyphenoloxydase, and their blast hits, scores, E-values, sizes, number of reads and conserved domains from putative proteins. [file gmb-33-4-795-suppl12.pdf]

Table S12: EST-Contigs with e-value < e^{-20} and score > 100 obtained in the Project Polyphenoloxydase, and their blast hit, score, e-value, size, number of reads, and conserved domains from putative proteins.

Polyphenoloxydase						
Contig	BLAST NR	Score	e-value	Length	Reads	Conserved Domains
1	gi|51872305|gb|AAU12257.1| polyphenol oxidase [Populus balsamifera subsp. trichocarpa x Populus deltoides]	616	0	1851	8	pfam00264
2	gi|1172584|sp|P43309|PPO_MALDO Polyphenol oxidase, chloroplast precursor (PPO) (Catechol oxidase) [Malus x domestica]	510	0	2127	11	pfam00264
3	gi|4158170|emb|CAA06855.1| catechol oxidase; polyphenol oxidase [Ipomoema batatas]	608	0	2070	30	pfam00264
4	gi|6137618|pdb|1BT3|A Chain A, Catechol Oxidase [Ipomoea Batatas]	315	2.00E-84	1000	14	pfam00264
