# Supplementary material for: In silico identification of coffee genome expressed sequences potentially associated with resistance to diseases
Source: Genet Mol Biol. 2010 Dec 1;33(4):795–806. doi: 10.1590/s1415-47572010000400031 (PMC3036153; doi:10.1590/s1415-47572010000400031)
Supplement: Table S13 — EST-contigs with E-values < e-20 and scores > 100 obtained in the project Importin, and their blast hits, scores, E-values, sizes, number of reads and conserved domains from putative proteins [file gmb-33-4-795-suppl13.pdf]

Table S13: EST-Contigs with e-value < e^{-20} and score > 100 obtained in the Project Importin, and their blast hit, score, e-value, size, number of reads, and conserved domains from putative proteins.

Importin						
Contig	BLAST NR	Score	e-value	Length	Reads	Conserved Domains
1	gi|15238758|ref|NP_200160.1| protein transporter [Arabidopsis thaliana]	668	0	1309	7	pfam03810, cd00020, COG5215
2	gi|13752562|gb|AAK38727.1|AF369707_1 importin alpha 2 [Capsicum annuum]	299	1E-79	807	3	cd00020, pfam01749, COG5064
4	gi|24940138|emb|CAD11990.1| rubisco small subunit [Coffea arabica]	163	8E-39	833	2	cd03527
5	gi|13752562|gb|AAK38727.1|AF369707_1 importin alpha 2 [Capsicum annuum]	555	0	1238	6	cd00020, pfam01749, COG5064
6	gi|84453224|dbj|BAE71209.1| putative importin alpha [Trifolium pratense]	223	3E-59	873	5	cd00020, pfam01749, COG5064
7	gi|13752562|gb|AAK38727.1|AF369707_1 importin alpha 2 [Capsicum annuum]	369	0	1029	4	cd00020, pfam01749, COG5064
8	gi|84453224|dbj|BAE71209.1| putative importin alpha [Trifolium pratense]	492	0	1482	12	cd00020, pfam01749, COG5064
9	gi|23954312|emb|CAC79691.1| Importin beta-like protein [Oryza sativa]	431	0	954	2	pfam03810, cd00020, COG5215
10	gi|84453224|dbj|BAE71209.1| putative importin alpha [Trifolium pratense]	128	4E-36	1147	2	cd00020, pfam01749, COG5064
11	gi|26449534|dbj|BAC41893.1| putative importin beta [Arabidopsis thaliana]	516	0	950	2	pfam03810, cd00020, COG5215
