# Supplementary material for: In silico identification of coffee genome expressed sequences potentially associated with resistance to diseases
Source: Genet Mol Biol. 2010 Dec 1;33(4):795–806. doi: 10.1590/s1415-47572010000400031 (PMC3036153; doi:10.1590/s1415-47572010000400031)
Supplement: Table S14 — EST-contigs with E-values < e-20 and scores > 100 obtained in the project Glucosyltransferase, and their blast hits, scores, E-values, sizes, number of reads and conserved domains from putative proteins. [file gmb-33-4-795-suppl14.pdf]

Table S14: EST-Contigs with e-value < e^{-20} and score > 100 obtained in the Project Glucosyltransferase, and their blast hit, score, e-value, size, number of reads, and conserved domains from putative proteins.

Glucosyltransferase						
Contig	BLAST NR	Score	e-value	Length	Reads	Conserved Domains
1	gi|147795324|emb|CAN67249.1| hypothetical protein [Vitis vinifera]	271	3.00E-71	671	2	pfam00201, COG1819
2	gi|147767625|emb|CAN60198.1| hypothetical protein [Vitis vinifera]	347	7.00E-94	1093	4	pfam00201, COG1819
3	gi|50284480|dbj|BAD29721.1| UDP-glucose glucosyltransferase [Catharanthus roseus]	476	0	1248	3	pfam00201
4	gi|147861933|emb|CAN78769.1| hypothetical protein [Vitis vinifera]	481	0	1128	5	pfam00201, COG1819
5	gi|119640513|gb|ABL85473.1| gylcosyltransferase UGT71A13 [Maclura pomifera]	120	1.00E-25	827	2	pfam00201, COG1819
6	gi|28380078|sp|Q9AR73|HQGT_RAUSE Hydroquinone glucosyltransferase (Arbutin synthase) [Rauvolfia serpentina]	580	0	1345	10	pfam00201, COG1819
7	gi|147821100|emb|CAN70963.1| hypothetical protein [Vitis vinifera]	358	1.00E-97	681	2	pfam00201, COG1819
9	gi|15228063|ref|NP_181234.1| UDP-glucoronosyl/UDP-glucosyl transferase family protein [Arabidopsis thaliana]	385	0	1184	8	pfam00201, COG1819
10	gi|147854062|emb|CAN83394.1| hypothetical protein [Vitis vinifera]	315	1.00E-84	710	2	pfam00201, COG1819
11	gi|125541419|gb|EAY87814.1| hypothetical protein OsI_009047 [Oryza sativa]	392	0	1124	5	COG0380, pfam02358, pfam00982
12	gi|454245|emb|CAA50376.1| anthocyanin 3 glucoside: rhamnosyltransferase [Petunia x hybrida]	422	0	1879	8	COG1819
13	gi|58430500|dbj|BAD89044.1| putative glycosyltransferase [Solanum aculeatissimum]	316	1.00E-84	906	3	pfam00201
14	gi|28380078|sp|Q9AR73|HQGT_RAUSE Hydroquinone glucosyltransferase (Arbutin synthase) [Rauvolfia serpentina]	459	0	1208	11	pfam00201, COG1819
15	gi|146148629|gb|ABQ02257.1| O-glucosyltransferase 2 [Vitis labrusca]	365	5.00E-99	1415	8	pfam00201, COG1819
17	gi|147855978|emb|CAN80742.1| hypothetical protein [Vitis vinifera]	473	0	1600	5	pfam00201, COG1819
18	gi|147846163|emb|CAN81633.1| hypothetical protein [Vitis vinifera]	310	8.00E-83	817	2	pfam00201, COG1819
19	gi|147767625|emb|CAN60198.1| hypothetical protein [Vitis vinifera]	257	4.00E-67	790	2	pfam00201, COG1819
20	gi|62241067|dbj|BAD93690.1| glycosyltransferase NTGT5b [Nicotiana tabacum]	299	1.00E-79	702	3	pfam00201
21	gi|147812173|emb|CAN61517.1| hypothetical protein [Vitis vinifera]	369	0	717	2	pfam00201, COG1819
22	gi|145329987|ref|NP_001077979.1| UDP-glucoronosyl/UDP-glucosyl transferase family protein [Arabidopsis thaliana]	199	1.00E-49	770	6	pfam00201, COG1819
23	gi|38532106|gb|AAR23313.1| beta-1,4-mannan synthase [Cyamopsis tetragonoloba]	290	7.00E-77	778	3	pfam00535, COG1215
24	gi|115467628|ref|NP_001057413.1| Os06g0288300 [Oryza sativa]	145	1.00E-40	972	6	pfam00201
25	gi|15236546|ref|NP_194913.1| PGR3 (PROTON GRADIENT REGULATION 3); binding [Arabidopsis thaliana]	291	4.00E-77	835	3	pfam01535, COG0457, pfam06239
26	gi|15242557|ref|NP_195906.1| pentatricopeptide (PPR) repeat-containing protein [Arabidopsis thaliana]	346	8.00E-94	743	2	pfam01535
27	gi|70734017|ref|YP_257657.1| lipopolysaccharide core biosynthesis protein RfaG [Pseudomonas fluorescens Pf-5]	405	0	817	2	pfam00534, COG0438
28	gi|147806166|emb|CAN70002.1| hypothetical protein [Vitis vinifera]	205	2.00E-51	722	4	pfam00201, COG1819
29	gi|4455123|gb|AAD21086.1| flavonoid 3-O-glucosyltransferase [Forsythia x intermedia]	273	0	1137	2	pfam00201
30	gi|34148023|gb|AAQ62571.1| glycosyltransferase 5 [Ipomoea trifida]	306	1.00E-81	901	3	pfam00535,COG1215
31	gi|21435782|gb|AAM53963.1|AF515727_1 UDP-glucosyltransferase [Stevia rebaudiana]	229	1.00E-58	714	2	pfam04101, pfam00201, COG1819
32	gi|70734017|ref|YP_257657.1| lipopolysaccharide core biosynthesisprotein RfaG [Pseudomonas fluorescens Pf-5]	438	0	858	7	pfam00534, COG0438
33	gi|629669|pir||S39507 glucuronosyl transferase homolog, ripening-related [Solanum lycopersicon]	180	7.00E-44	819	2	pfam00201
34	gi|62241065|dbj|BAD93689.1| glycosyltransferase NTGT5a [Nicotiana tabacum]	496	0	1523	13	pfam00201
35	gi|20149064|gb|AAM12787.1| putative anthocyanidine rhamnosyl-transferase [Capsicum annuum]	380	0	1696	4	COG1819
36	gi|19568935|gb|AAL91978.1|AF483209_1 putative trehalose synthase [Solanum tuberosum]	599	0	1103	4	COG0380, pfam02358, pfam00982
37	gi|22759895|dbj|BAC10994.1| rhamnosyl transferase [Nierembergia sp. NB17]	216	1.00E-54	693	2	pfam00201, COG1819
38	gi|15224213|ref|NP_179460.1| ATTPS11 [Arabidopsis thaliana]	304	3.00E-81	755	2	COG0380, pfam02358, pfam00982
39	gi|147795873|emb|CAN74227.1| hypothetical protein [Vitis vinifera]	385	0	1078	4	pfam00201, COG1819
40	gi|50284482|dbj|BAD29722.1| UDP-glucose glucosyltransferase [Catharanthus roseus]	630	0	1588	6	pfam00201
41	gi|126668372|ref|ZP_01739330.1| glycosyl transferases-like protein [Marinobacter sp. ELB17]	296	5.00E-79	729	3	pfam00535, PRK10714
42	gi|51705431|gb|AAU09445.1| putative UDP-rhamnose:rhamnosyltransferase [Fragaria x ananassa]	217	9.00E-66	1114	5	pfam00201
43	gi|119640480|gb|ABL85472.1| glycosyltransferase UGT72B9 [Maclura pomifera]	114	2.00E-24	516	3	pfam00201
44	gi|147768453|emb|CAN78332.1| hypothetical protein [Vitis vinifera]	243	9.00E-63	842	2	pfam00201, COG1819
45	gi|77457186|ref|YP_346691.1| alginate biosynthesis protein Alg8 [Pseudomonas fluorescens PfO-1]	400	0	869	3	No CD has been identified
46	gi|147767625|emb|CAN60198.1| hypothetical protein [Vitis vinifera]	217	5.00E-55	685	3	pfam00201, COG1819
47	gi|92877343|gb|ABE84479.1| Pentatricopeptide repeat [Medicago truncatula]	167	6.00E-40	719	2	pfam01535
48	gi|21435782|gb|AAM53963.1|AF515727_1 UDP-glucosyltransferase [Stevia rebaudiana]	410	0	1639	6	pfam04101, pfam00201, COG1819
49	gi|51705413|gb|AAU09444.1| UDP-glucose glucosyltransferase [Fragaria anassa]	375	0	1280	4	pfam00201
50	gi|147815573|emb|CAN68287.1| hypothetical protein [Vitis vinifera]	266	8.00E-70	750	3	pfam00201, COG1819
51	gi|147781122|emb|CAN71907.1| hypothetical protein [Vitis vinifera]	255	2.00E-66	788	2	pfam00201, COG1819
52	gi|20146093|dbj|BAB88935.1| glucosyltransferase NTGT2 [Nicotiana tabacum]	655	0	1831	18	pfam03033, COG1819
53	gi|147811099|emb|CAN70169.1| hypothetical protein [Vitis vinifera]	362	0	1517	11	pfam00201, COG1819
54	gi|147846160|emb|CAN79485.1| hypothetical protein [Vitis vinifera]	461	0	1037	2	pfam00201, COG1819
55	gi|15227717|ref|NP_180576.1| UDP-glucoronosyl/UDP-glucosyl transferase family protein [Arabidopsis haliana]	408	0	1532	5	COG1819
56	gi|115473997|ref|NP_001060597.1| Os07g0671200 [Oryza sativa]	160	3.00E-38	571	2	pfam01535
57	gi|24459979|dbj|BAC22617.1| UDP-glucose:sterol 3-O-glucosyltransferase [Panax ginseng]	511	0	809	3	pfam03033, COG1819
58	gi|147855978|emb|CAN80742.1| hypothetical protein [Vitis vinifera]	310	8.00E-83	961	5	pfam00201, COG1819
59	gi|14349251|dbj|BAB60720.1| glucosyltransferase [Nicotiana tabacum]	544	0	1366	7	pfam00201
60	gi|147815573|emb|CAN68287.1| hypothetical protein [Vitis vinifera]	226	3.00E-60	812	2	pfam00201, COG1819
61	gi|14192682|gb|AAK54465.1| cold-induced glucosyl transferase [Solanum tuberosum]	192	2.00E-52	768	2	pfam03033, pfam00201, COG1819
62	gi|116310407|emb|CAH67416.1| OSIGBa0143N19.10 [Oryza sativa]	290	2.00E-78	1123	2	pfam01501
63	gi|115310620|emb|CAJ32597.1| sucrose synthase [Coffea arabica]	483	0	871	3	pfam00534, pfam00862
64	gi|147787516|emb|CAN77812.1| hypothetical protein [Vitis vinifera]	151	3.00E-35	714	3	pfam00201, COG1819
65	gi|147864250|emb|CAN83017.1| hypothetical protein [Vitis vinifera]	229	5.00E-60	969	2	pfam00201, COG1819
66	gi|147811099|emb|CAN70169.1| hypothetical protein [Vitis vinifera]	106	1.00E-37	968	4	pfam00201, COG1819
67	gi|147795324|emb|CAN67249.1| hypothetical protein [Vitis vinifera]	232	2.00E-59	709	3	pfam00201, COG1819
68	gi|147800590|emb|CAN77507.1| hypothetical protein [Vitis vinifera]	148	1.00E-34	522	2	pfam00201, COG1819
69	gi|147818360|emb|CAN62624.1| hypothetical protein [Vitis vinifera]	202	3.00E-68	804	2	pfam00201, COG1819

70	gi|147821441|emb|CAN74580.1| hypothetical protein [Vitis vinifera]	358	1.00E-97	801	3	pfam00201, COG1819
71	gi|125553006|gb|EAY98715.1| hypothetical protein OsI_019948 [Oryza sativa]	801	0	2030	8	COG0380, pfam02358, pfam00982
72	gi|115310620|emb|CAJ32597.1| sucrose synthase [Coffea arabica]	528	0	1066	3	pfam00534, pfam00862
73	gi|27461029|gb|AAL06646.1| flavonoid 1-2 rhamnosyltransferase [Citrus maxima]	241	4.00E-62	810	2	COG1819
74	gi|15225508|ref|NP_181493.1| glycosyl transferase family 2 protein [Arabidopsis thaliana]	469	0	1434	6	pfam00535
75	gi|140049083|gb|ABO79665.1| Glycosyl transferase, family 8 [Medicago truncatula]	239	4.00E-61	1408	8	pfam01501
76	gi|147809370|emb|CAN71202.1| hypothetical protein [Vitis vinifera]	775	0	1312	2	pfam00201, COG1819
77	gi|147855978|emb|CAN80742.1| hypothetical protein [Vitis vinifera]	305	4.00E-81	1064	3	pfam00201, COG1819
78	gi|147828262|emb|CAN62185.1| hypothetical protein [Vitis vinifera]	337	4.00E-91	782	2	pfam00201, COG1819
79	gi|115467634|ref|NP_001057416.1| Os06g0289200 [Oryza sativa]	251	2.00E-84	1332	7	pfam00201
80	gi|147855615|emb|CAN83466.1| hypothetical protein [Vitis vinifera]	478	0	1008	5	pfam00201, COG1819
81	gi|147767625|emb|CAN60198.1| hypothetical protein [Vitis vinifera]	495	0	1544	5	pfam00201, COG1819
82	gi|147826555|emb|CAN61971.1| hypothetical protein [Vitis vinifera]	380	0	757	3	pfam00201, COG1819
83	gi|62241063|dbj|BAD93688.1| glucosyltransferase NTGT4 [Nicotiana tabacum]	247	4.00E-64	652	2	pfam00201
84	gi|102139944|gb|ABF70084.1| trehalose-6-phosphate synthase, putative [Musa balbisiana]	128	4.00E-28	818	2	COG0380, pfam02358, pfam00982
85	gi|147862190|emb|CAN82595.1| hypothetical protein [Vitis vinifera]	891	0	2586	16	pfam00201, COG1819
86	gi|110932098|gb|ABH03018.1| resveratrol/hydroxycinnamic acid O-glucosyltransferase [Vitis labrusca]	708	0	1719	15	pfam00201, COG1819
87	gi|52139814|gb|AAU29197.1| sucrose phosphate synthase [Lycopersicon esculentum]	570	0	1062	3	pfam00534, pfam05116
88	gi|147779442|emb|CAN74357.1| hypothetical protein [Vitis vinifera]	509	0	830	2	pfam00201, COG1819
89	gi|67845751|emb|CAI56307.1| sucrose synthase [Coffea canephora]	1625	0	3092	191	pfam00534, pfam00862
90	gi|147818682|emb|CAN76185.1| hypothetical protein [Vitis vinifera]	1157	0	2346	16	pfam00201, COG1819
91	gi|20149064|gb|AAM12787.1| putative anthocyanidine rhamnosyl-transferase [Capsicum annuum]	196	2.00E-49	360	2	COG1819
93	gi|15235900|ref|NP_192536.1| ATCSLC12 (Cellulose synthase-like C12); transferase, transferring glycosyl groups [Arabidopsis thaliana]	956	0	2097	7	pfam00535
94	gi|115310620|emb|CAJ32597.1| sucrose synthase [Coffea arabica]	889	0	1326	8	pfam00534, pfam00862
95	gi|115310618|emb|CAJ32596.1| sucrose synthase [Coffea arabica]	401	0	851	4	pfam00534, pfam00862
96	gi|20146093|dbj|BAB88935.1| glucosyltransferase NTGT2 [Nicotiana tabacum]	544	0	1315	8	pfam03033, COG1819
97	gi|42795466|gb|AAS46243.1| xyloglucan endotransglucosylase-hydrolase XTH7 [Lycopersicon esculentum]	392	0	889	14	cd02176, pfam06955
98	gi|147833323|emb|CAN61988.1| hypothetical protein [Vitis vinifera]	320	5.00E-86	769	2	pfam00201, COG1819
99	gi|67845751|emb|CAI56307.1| sucrose synthase [Coffea canephora]	474	0	1024	3	pfam00534, pfam00862
100	gi|147800590|emb|CAN77507.1| hypothetical protein [Vitis vinifera]	397	0	1376	5	pfam00201, COG1819
101	gi|147768350|emb|CAN60444.1| hypothetical protein [Vitis vinifera]	349	7.00E-95	726	2	pfam00201, COG1819
102	gi|147815573|emb|CAN68287.1| hypothetical protein [Vitis vinifera]	207	2.00E-63	898	3	pfam00201, COG1819
103	gi|147768350|emb|CAN60444.1| hypothetical protein [Vitis vinifera]	166	5.00E-40	541	2	pfam00201, COG1819
104	gi|115310618|emb|CAJ32596.1| sucrose synthase [Coffea arabica]	279	2.00E-88	997	2	pfam00534, pfam00862
105	gi|147815573|emb|CAN68287.1| hypothetical protein [Vitis vinifera]	309	2.00E-82	902	11	pfam00201, COG1819
106	gi|145337405|ref|NP_177278.3| UDP-glucose:glycoprotein glucosyltransferase [Arabidopsis thaliana]	572	0	1477	10	pfam06427, pfam01501
107	gi|37993657|gb|AAR06914.1| UDP-glycosyltransferase 71E1 [Stevia rebaudiana]	286	7.00E-76	735	2	pfam00201
109	gi|27461029|gb|AAL06646.1| flavonoid 1-2 rhamnosyltransferase [Citrus maxima]	198	3.00E-49	764	3	COG1819
110	gi|15226332|ref|NP_180375.1| glycosyltransferase family protein [Arabidopsis thaliana]	434	0	1178	2	pfam00201, COG1819
111	gi|19743740|gb|AAL92461.1| putative glucosyltransferase [Lycopersicon esculentum]	361	0	1183	3	pfam00201
112	gi|147811099|emb|CAN70169.1| hypothetical protein [Vitis vinifera]	108	3.00E-22	844	2	pfam00201, COG1819
113	gi|147790314|emb|CAN74373.1| hypothetical protein [Vitis vinifera]	206	9.00E-52	824	2	pfam00201, COG1819
114	gi|147787516|emb|CAN77812.1| hypothetical protein [Vitis vinifera]	203	9.00E-51	783	5	pfam00201, COG1819
115	gi|147790314|emb|CAN74373.1| hypothetical protein [Vitis vinifera]	255	2.00E-66	754	2	pfam00201, COG1819
116	gi|147768688|emb|CAN76057.1| hypothetical protein [Vitis vinifera]	462	0	1759	6	pfam00201, COG1819
117	gi|15228063|ref|NP_181234.1| UDP-glucoronosyl/UDP-glucosyl transferase family protein [Arabidopsis thaliana]	560	0	1316	2	pfam06427, pfam01501
118	gi|22326571|ref|NP_195861.2| DIE2/ALG10 family [Arabidopsis thaliana]	333	0	1670	7	pfam04922
119	gi|147815573|emb|CAN68287.1| hypothetical protein [Vitis vinifera]	301	2.00E-80	651	3	pfam00201, COG1819
121	gi|20146093|dbj|BAB88935.1| glucosyltransferase NTGT2 [Nicotiana tabacum]	280	8.00E-74	967	7	pfam03033, COG1819
122	gi|62241067|dbj|BAD93690.1| glycosyltransferase NTGT5b [Nicotiana tabacum]	307	4.00E-82	769	5	pfam00201
123	gi|146148629|gb|ABQ02257.1| O-glucosyltransferase 2 [Vitis labrusca]	342	3.00E-92	1235	7	pfam00201, COG1819
124	gi|147855978|emb|CAN80742.1| hypothetical protein [Vitis vinifera]	302	4.00E-82	1097	2	pfam00201, COG1819
125	gi|19743740|gb|AAL92461.1| putative glucosyltransferase [Lycopersicon esculentum]	451	0	1314	5	pfam00201
126	gi|77176831|gb|ABA64521.1| sucrose-phosphate synthase isoform B [Nicotiana tabacum]	579	0	1022	3	pfam00534, pfam05116, pfam00862
127	gi|147790314|emb|CAN74373.1| hypothetical protein [Vitis vinifera]	514	0	1819	16	pfam00201, COG1819
128	gi|629669|pir||S39507 glucuronosyl transferase homolog, ripening-related [Solanum lycopersicon]	455	0	1256	2	pfam00201
129	gi|147790314|emb|CAN74373.1| hypothetical protein [Vitis vinifera]	228	6.00E-58	1034	3	pfam00201, COG1819
130	gi|92884230|gb|ABE87250.1| UDP-glucuronosyl/UDP-glucosyltransferase [Medicago truncatula]	137	6.00E-62	841	2	pfam00201
