# Supplementary material for: In silico identification of coffee genome expressed sequences potentially associated with resistance to diseases
Source: Genet Mol Biol. 2010 Dec 1;33(4):795–806. doi: 10.1590/s1415-47572010000400031 (PMC3036153; doi:10.1590/s1415-47572010000400031)
Supplement: Table S15 — ESTs from the NS1, RM1, RX1 and SS1 libraries shown in Figure 1. [file gmb-33-4-795-suppl15.pdf]

Table S15: ESTs from NS1, RM1, RX1 and SS1 libraries, shown in Figure 1.

Project NBS-LRR			
ESTs NS1	BLAST X	E-value	Size (bp)
1	P0039H02.32 [Oryza sativa (japonica cultivar-group)]	3.00E-11	797
2	rad-51 (Fifty one) like, Short RFS-1, f-box protein Fbl2 (51.6 kD) (rfs-1Co) [Caenorhabditis elegans]	0.005	871
3	NBS-LRR protein [Solanum acaule]	7.00E-13	889
4	F-box and leucine-rich repeat protein 2; F-box protein containing leucine-rich repeats [Homo sapiens]	3.00E-09	919
ESTs RM1	BLAST X	E-value	Size (bp)
1	NBS-LRR-like protein [Oryza sativa (japonica cultivar-group)]	9.00E-26	908
2	NBS-LRR resistance-like protein J71 [Phaseolus vulgaris]	1.00E-26	908
3	hypothetical protein F6H11.60 - Arabidopsis thaliana	4.00E-33	987
4	hypothetical protein F6H11.60 - Arabidopsis thaliana	0.001	981
5	elicitor-inducible LRR receptor-like protein EILP [Nicotiana tabacum]	5.00E-34	878
6	disease resistance protein (CC-NBS-LRR class), putative [Arabidopsis thaliana]	4.00E-13	872
7	LRR receptor-like protein kinase [Nicotiana tabacum]	5.00E-90	914
8	putative TIR/NBS/LRR disease resistance protein [Pinus taeda]	6.00E-08	889
9	disease resistance protein (CC-NBS-LRR class), putative [Arabidopsis thaliana]	1.00E-43	930
10	leucine-rich repeat transmembrane protein kinase, putative [Arabidopsis thaliana]	4.00E-21	901
11	disease resistance protein (TIR-NBS-LRR class), putative [Arabidopsis thaliana]	3.00E-07	925
12	hypernodulation aberrant root formation protein [Lotus japonicus]	8.00E-26	809
13	LRR receptor-like kinase 2 [Arabidopsis thaliana]	1.00E-07	871
14	similar to NBS-LRR type resistance gene [Oryza sativa (japonica cultivar-group)]	3.00E-46	923
15	leucine-rich repeat transmembrane protein kinase, putative [Arabidopsis thaliana]	7.00E-31	922
ESTs RX1	BLAST X	E-value	Size (bp)
1	disease resistance protein family (LRR) [Arabidopsis thaliana]	3.00E-05	957
2	LRR receptor protein kinase -related [Arabidopsis thaliana]	1.00E-16	972
3	NBS-LRR protein [Solanum acaule]	1.00E-17	837
4	Phytosulfokine receptor precursor (Phytosulfokine LRR receptor kinase)	2.00E-08	966
5	leucine-rich repeat protein LRP - tomato	5.00E-56	932
6	disease resistance protein (CC-NBS-LRR class), putative [Arabidopsis thaliana]	2.00E-06	960
7	disease resistance protein (NBS-LRR class), putative [Arabidopsis thaliana]	0.0004	1008
8	NBS-LRR disease resistance protein homologue [Hordeum vulgare]	5.00E-29	988
9	LRR receptor-like kinase 1 [Arabidopsis thaliana]	0.004	967
10	NBS-LRR protein [Solanum acaule]	5.00E-17	1017
11	disease resistance protein (CC-NBS-LRR class), putative [Arabidopsis thaliana]	2.00E-08	889
12	coronatine-insensitive 1 (COI1), AtFBL2 [Arabidopsis thaliana]	7.00E-05	983
13	coronatine-insensitive 1 (COI1), AtFBL2 [Arabidopsis thaliana]	3.00E-07	976
14	NBS-LRR protein [Solanum acaule]	5.00E-13	857
15	disease resistance protein (CC-NBS-LRR class), putative [Arabidopsis thaliana]	9.00E-07	882
16	putative NBS-LRR type resistance protein, 3' partial [Oryza sativa]	0.0004	908
ESTs SS1	BLAST X	E-value	Size (bp)
1	NBS-LRR protein [Solanum tuberosum]	3.00E-24	888
2	GPA2-like NBS-LRR protein [Solanum nigrum]	2.00E-15	878
3	P0039H02.32 [Oryza sativa (japonica cultivar-group)]	0.0002	874
Project Resistance			
ESTs NS1	BLAST X	E-value	Size (bp)
1	disease resistance protein Prf - tomato	2.00E-14	889
ESTs RM1	BLAST X	E-value	Size (bp)
1	hypothetical protein Rv1258c [Mycobacterium tuberculosis H37Rv]	1.00E-05	907
2	fusaric acid resistance protein [Xanthomonas campestris pv. campestris str. ATCC 33913]	1.00E-15	881
3	leucine rich repeat protein family [Arabidopsis thaliana]	9.00E-38	876
4	pleiotropic drug resistance like protein [Nicotiana tabacum]	7.00E-09	905
5	Putative disease resistance protein At1g58400	4.00E-13	872
6	daunorubicin resistance protein [Enterococcus faecalis V583]	1.00E-17	854
7	disease resistance protein family [Arabidopsis thaliana]	3.00E-34	878
8	orf, hypothetical protein [Escherichia coli K12]	9.00E-52	884
9	orf, hypothetical protein [Escherichia coli K12]	3.00E-42	845
10	Acriflavin resistance protein F (EnvD protein). [Escherichia coli]	0.002	976
11	dTDP-glucose 4-6-dehydratase homolog D18 - Arabidopsis thaliana	1.00E-35	925
12	[Segment 1 of 2] Mercuric reductase (Hg(II) reductase)	3.00E-29	933
13	Glyoxalase, Glyoxalase/Bleomycin resistance protein/Dioxygenase superfamily [Bacillus anthracis A2012]	3.00E-19	973
14	putative Cf2/Cf5 disease resistance protein [Oryza sativa (japonica cultivar-group)]	1.00E-31	941
15	leucine rich repeat protein family [Arabidopsis thaliana]	0.003	963
16	leucine rich repeat protein family [Arabidopsis thaliana]	6.00E-11	939
17	Multidrug resistance ABC transporter ATP-binding and permease protein [Bacillus cereus ATCC 14579]	5.00E-09	945
18	D-alanyl-D-alanine dipeptidase (D-Ala-D-Ala dipeptidase) (Vancomycin B-type resistance protein vanX)	6.00E-15	943
19	membrane protein OpdE [Pseudomonas aeruginosa PA01]	6.00E-24	902
20	integral membrane lipid kinase-like protein [Mesorhizobium loti]	3.00E-17	947
21	expressed protein [Arabidopsis thaliana]	8.00E-40	921
22	orf, hypothetical protein [Escherichia coli K12]	3.00E-53	635
23	pleiotropic drug resistance like protein [Nicotiana tabacum]	4.00E-34	809
24	FUSARIC ACID RESISTANCE PROTEIN FUSE [Brucella melitensis]	2.00E-05	947
25	fusaric acid resistance protein, putative [Pseudomonas putida KT2440]	9.00E-08	943
26	pleiotropic drug resistance like protein [Nicotiana tabacum]	4.00E-43	908
27	disease resistance-like protein [Coffea arabica]	2.00E-71	908
28	dTDP-glucose 4-6-dehydratase homolog D18 - Arabidopsis thaliana	2.00E-31	923
29	disease resistance-like protein [Coffea arabica]	3.00E-68	908

30	senescence-associated protein [Arabidopsis thaliana]	2.00E-33	944
31	putative TIR/NBS/LRR disease resistance protein [Pinus taeda]	6.00E-08	889
32	Tetracenomycin C resistance and export protein	3.00E-18	972
33	FUSARIC ACID RESISTANCE PROTEIN FUSB / FUSARIC ACID RESISTANCE PROTEIN FUSC [Brucella melitensis]	3.00E-05	1040
34	dTDP-glucose 4-6-dehydratase homolog D18 - Arabidopsis thaliana	2.00E-15	924
35	ACRIFLAVIN RESISTANCE PROTEIN D [Brucella melitensis]	8.00E-11	929
36	similar to Pib(rice blast resistance gene) [Oryza sativa (japonica cultivar-group)]	8.00E-49	923
37	natural resistance-associated macrophage protein [Burkholderia cepacia genomovar III]	3.00E-28	939
38	golgi-specific brefeldin A-resistance guanine nucleotide exchange factor 1 (3M842) [Caenorhabditis elegans]	1.00E-08	956
39	multidrug resistance P-glycoprotein, putative [Arabidopsis thaliana]	5.00E-54	1022
40	outer membrane protein OprM precursor [Pseudomonas aeruginosa PA01]	0.003	985
41	PROBABLE RESPONSE REGULATOR FOR COBALT ZINC CADMIUM RESISTANCE TRANSCRIPTION REGULATOR PROTEIN [Ralstonia solanacearum]	4.00E-71	921
42	putative RSH, disease resistance-related protein [Oryza sativa (japonica cultivar-group)]	6.00E-94	895
43	DAUNORUBICIN RESISTANCE TRANSMEMBRANE PROTEIN [Brucella melitensis]	7.00E-19	999
44	cobalt-zinc-cadmium resistance protein [Xylella fastidiosa Temecula1]	5.00E-05	921
45	multidrug resistance protein [Bradyrhizobium japonicum]	3.00E-43	912
46	fusaric acid resistance protein [Xanthomonas campestris pv. campestris str. ATCC 33913]	2.00E-16	857
47	multidrug resistance ABC transporter [Clostridium tetani E88]	1.00E-09	884
48	multidrug resistance ABC transporter [Clostridium tetani E88]	1.00E-07	912
49	disease resistance protein (CC-NBS-LRR class), putative [Arabidopsis thaliana]	1.00E-43	930
50	PROBABLE RESPONSE REGULATOR FOR COBALT ZINC CADMIUM RESISTANCE TRANSCRIPTION REGULATOR PROTEIN [Ralstonia solanacearum]	5.00E-69	909
51	dTDP-glucose 4-6-dehydratase homolog D18 - Arabidopsis thaliana	4.00E-50	933
52	disease resistance protein (TIR-NBS-LRR class), putative [Arabidopsis thaliana]	3.00E-07	925
53	Tetracycline resistance protein from transposon Tn4351/Tn4400	5.00E-14	926
54	florfenicol resistance protein-like [Oryza sativa (japonica cultivar-group)]	2.00E-16	913
55	pleiotropic drug resistance like protein [Nicotiana tabacum]	3.00E-14	950
56	tellurium resistance protein TerC [Rickettsia conorii]	2.00E-07	948
57	PROBABLE MULTIDRUG RESISTANCE TRANSMEMBRANE PROTEIN [Ralstonia solanacearum]	4.00E-30	976
58	Tetracycline resistance protein from transposon Tn4351/Tn4400	4.00E-20	903
59	bacteriocin resistance protein, putative [Neisseria meningitidis MC58]	1.00E-07	906
60	fusaric acid resistance protein, putative [Pseudomonas putida KT2440]	0	875
61	bacteriocin resistance protein, putative [Neisseria meningitidis MC58]	4.00E-07	909
**ESTs RX1**	**BLAST X**	**E-value**	**Size (bp)**
1	disease resistance protein [Lycopersicon esculentum]	5.00E-11	966
2	disease resistance protein BS2 [Capsicum chacoense]	3.00E-16	837
3	Putative disease resistance protein Hcr2-0B [Oryza sativa]	2.00E-12	952
4	multidrug resistance protein, putative [Vibrio cholerae]	9.00E-52	890
5	isease resistance E - tomato	4.00E-49	929
6	disease resistance protein -related [Arabidopsis thaliana]	1.00E-05	957
7	verticillium wilt disease resistance protein Ve2 [Lycopersicon esculentum]	1.00E-29	949
8	putative Cf2/Cf5 disease resistance protein [Oryza sativa (japonica cultivar-group)]	5.00E-22	940
9	probable disease resistance protein [imported] - Arabidopsis thaliana	2.00E-08	889
10	putative Mlo (pathogen resistance) protein [Oryza sativa]	1.00E-15	915
11	hypothetical protein T3F20.24 [imported] - Arabidopsis thaliana	2.00E-17	934
12	dTDP-glucose 4-6-dehydratase homolog D18 - Arabidopsis thaliana	3.00E-05	944
13	disease resistance protein D - tomato	1.00E-48	941
14	putative resistance protein [Lycopersicon esculentum]	6.00E-72	948
15	fusaric acid resistance protein, putative [Pseudomonas putida KT2440]	1.00E-13	959
16	disease resistance-like protein [Coffea canephora]	4.00E-22	1017
17	disease resistance protein Cf-2.1 - currant tomato	1.00E-18	992
18	ribosomal protein S14; 40S ribosomal protein S14; emetine resistance [Homo sapiens]	3.00E-52	962
19	Probable WRKY transcription factor 19 (WRKY DNA-binding protein 19)	5.00E-13	927
20	similar to Pib(rice blast resistance gene) [Oryza sativa (japonica cultivar-group)]	3.00E-29	988
21	disease resistance protein (NBS-LRR class), putative [Arabidopsis thaliana]	0.0004	1008
22	dTDP-glucose 4-6-dehydratase homolog D18 - Arabidopsis thaliana	2.00E-10	967
23	disease resistance protein Hcr2-5D - tomato	4.00E-12	971
24	disease resistance protein Prf - tomato	1.00E-14	960
25	hypothetical protein T3F20.24 [imported] - Arabidopsis thaliana	5.00E-22	955
26	dTDP-glucose 4-6-dehydratase homolog D18 - Arabidopsis thaliana	2.00E-96	1036
27	dTDP-glucose 4-6-dehydratase homolog D18 - Arabidopsis thaliana	1.00E-37	945
28	pleiotropic drug resistance like protein [Nicotiana tabacum]	3.00E-07	977
29	disease resistance response protein-related/ dirigent protein-related [Arabidopsis thaliana]	8.00E-32	912
30	probable fusaric acid resistance protein FusE II [Chromobacterium violaceum ATCC 12472]	2.00E-16	926
31	ribosomal protein S14; 40S ribosomal protein S14; emetine resistance [Homo sapiens]	9.00E-53	921
32	probable multidrug resistance efflux pump [Pseudomonas aeruginosa PA01]	1.00E-47	938
33	putative disease resistance protein [Oryza sativa]	6.00E-28	919
34	probable oxidative stress resistance two-component transmembrane sensor histidine kinase transcription regulator protein [Ralstonia solanacearum]	2.00E-14	916
35	barley stem rust resistance protein [Hordeum vulgare subsp. vulgare]	4.00E-06	891
36	SUMO-1 activating enzyme subunit 2 [Homo sapiens]	2.00E-25	901
37	SUMO-1 activating enzyme subunit 2 [Homo sapiens]	3.00E-58	883
38	multidrug resistance P-glycoprotein (pgp1) [Arabidopsis thaliana]	5.00E-18	837
39	disease resistance protein Prf - tomato	5.00E-23	857
40	disease resistance protein Prf - tomato	7.00E-33	882
41	putative NBS-LRR type resistance protein, 3' partial [Oryza sativa]	0.0004	908
42	drug resistance transporter, Bcr/CflA family protein [Pseudomonas syringae pv. tomato str. DC3000]	6.00E-47	923
**ESTs SS1**	**BLAST X**	**E-value**	**Size (bp)**
1	pleiotropic drug resistance like protein [Nicotiana tabacum]	5.00E-27	890

2	disease resistance protein BS2 [Capsicum chacoense]	7.00E-28	878
3	disease resistance protein BS2 [Capsicum chacoense]	1.00E-31	888
4	disease resistance protein (TIR class), putative [Arabidopsis thaliana]	1.00E-13	895
5	senescence-associated protein [Arabidopsis thaliana]	2.00E-32	861
6	pleiotropic drug resistance like protein [Nicotiana tabacum]	5.00E-40	861
7	disease resistance protein Prf - tomato	0.0002	862
**Project Chitinase**			
**ESTs NS1**	**BLAST X**	**E-value**	**Size (bp)**
1	chitinase [Hevea brasiliensis]	2.00E-41	846
**ESTs RM1**	**BLAST X**	**E-value**	**Size (bp)**
1	chitinase (EC 3.2.1.14) Chib1 - soybean	4.00E-51	953
2	glycosyl hydrolase family 19 (chitinase) [Arabidopsis thaliana]	7.00E-87	783
3	chitinase 3-like protein precursor [Trichosanthes kirilowii]	3.00E-32	993
4	chitinase (EC 3.2.1.14) class III, acidic - soybean	2.00E-20	964
5	chitinase (EC 3.2.1.14) class III, acidic - soybean	5.00E-15	902
6	chitinase 3-like protein precursor [Trichosanthes kirilowii]	4.00E-49	935
7	chitinase 3-like protein precursor [Trichosanthes kirilowii]	8.00E-45	881
8	class VII chitinase precursor [Gossypium hirsutum]	6.00E-76	938
**ESTs RX1**	**BLAST X**	**E-value**	**Size (bp)**
1	chitinase III [Vitis vinifera]	2.00E-65	994
2	putative class III acidic chitinase [Oryza sativa (japonica cultivar-group)]	5.00E-64	941
3	chitinase [Hevea brasiliensis]	2.00E-52	941
4	chitinase (EC 3.2.1.14) - adzuki bean	2.00E-28	936
5	chitinase (EC 3.2.1.14) Chib1 - soybean	2.00E-60	918
6	chitinase homolog [Coffea arabica]	7.00E-45	983
7	ACIDIC ENDOCHITINASE PRECURSOR	3.00E-36	963
8	chitinase 3-like protein precursor [Trichosanthes kirilowii]	1.00E-65	904
9	chitinase homolog [Coffea arabica]	5.00E-53	913
10	chitinase [Psophocarpus tetragonolobus]	2.00E-60	875
11	chitinase [Trifolium repens]	1.00E-43	924
12	ACIDIC ENDOCHITINASE PRECURSOR	6.00E-67	870
13	chitinase [Hevea brasiliensis]	1.00E-50	907
14	chitinase [Hevea brasiliensis]	2.00E-75	968
15	chitinase precursor [Petroselinum crispum]	0	974
16	chitinase [Hevea brasiliensis]	1.00E-45	930
17	class III acidic chitinase [Malus x domestica]	1.00E-64	959
18	class VII chitinase precursor [Gossypium hirsutum]	6.00E-44	967
19	ACIDIC ENDOCHITINASE SE2 PRECURSOR	2.00E-55	984
20	probable chitinase (EC 3.2.1.14) precursor - cucumber	1.00E-16	933
21	chitinase homolog [Coffea arabica]	2.00E-38	1016
22	class III chitinase [Lupinus albus]	1.00E-66	962
23	chitinase 3-like protein precursor [Trichosanthes kirilowii]	4.00E-39	958
24	chitinase [Hevea brasiliensis]	1.00E-48	1016
25	chitinase 3-like protein precursor [Trichosanthes kirilowii]	6.00E-62	916
26	ACIDIC ENDOCHITINASE PRECURSOR	2.00E-57	952
27	chitinase (EC 3.2.1.14) class III, acidic - soybean	9.00E-18	927
28	chitinase 1 [Cucumis melo]	4.00E-18	917
29	class III chitinase [Lupinus albus]	1.00E-27	945
30	chitinase homolog [Coffea arabica]	3.00E-06	827
31	ACIDIC ENDOCHITINASE PRECURSOR	1.00E-38	925
32	chitinase [Psophocarpus tetragonolobus]	2.00E-63	959
**ESTs SS1**	**BLAST X**	**E-value**	**Size (bp)**
1	chitinase 3-like protein precursor [Trichosanthes kirilowii]	1.00E-35	866
2	chitinase 3-like protein precursor [Trichosanthes kirilowii]	4.00E-25	869
3	class III chitinase-like protein [Sesbania rostrata]	0.0006	872
4	chitinase 3-like protein precursor [Trichosanthes kirilowii]	4.00E-12	862
5	probable chitinase (EC 3.2.1.14) precursor - cucumber	5.00E-14	856
6	chitinase (EC 3.2.1.14) class III, acidic - soybean	2.00E-18	861
7	ACIDIC ENDOCHITINASE PRECURSOR	1.00E-83	868
8	chitinase 3-like protein precursor [Trichosanthes kirilowii]	3.00E-26	860
9	chitinase 3-like protein precursor [Trichosanthes kirilowii]	5.00E-20	874
10	chitinase 3-like protein precursor [Trichosanthes kirilowii]	4.00E-27	875
11	probable chitinase (EC 3.2.1.14) precursor - cucumber	7.00E-13	890
12	probable chitinase (EC 3.2.1.14) precursor - cucumber	5.00E-16	865
13	chitinase 3-like protein precursor [Trichosanthes kirilowii]	5.00E-53	854
14	chitinase (EC 3.2.1.14) class III, acidic - soybean	4.00E-13	835
15	probable chitinase (EC 3.2.1.14) precursor - cucumber	3.00E-14	875
16	ACIDIC ENDOCHITINASE PRECURSOR	6.00E-74	883
17	Glycosidase, Chitin Degradation, Multifunctional Enzyme	1.00E-53	911
18	chitinase homolog [Coffea arabica]	3.00E-53	894
19	chitinase 3-like protein precursor [Trichosanthes kirilowii]	7.00E-34	870
20	chitinase [Psophocarpus tetragonolobus]	4.00E-66	865
21	ACIDIC ENDOCHITINASE PRECURSOR	3.00E-68	878
22	chitinase [Hevea brasiliensis]	4.00E-70	881
23	chitinase [Hevea brasiliensis]	2.00E-62	884
24	ACIDIC ENDOCHITINASE PRECURSOR	1.00E-62	873
25	class III chitinase-like protein [Sesbania rostrata]	0.0002	881

26	ACIDIC ENDOCHITINASE PRECURSOR	6.00E-74	881
27	probable chitinase (EC 3.2.1.14) precursor - cucumber	4.00E-16	883
28	chitinase (EC 3.2.1.14) class III, acidic - soybean	7.00E-18	875
29	ACIDIC ENDOCHITINASE PRECURSOR	9.00E-72	887
30	class III acidic chitinase [Musa acuminata]	2.00E-47	879
31	ACIDIC ENDOCHITINASE PRECURSOR	7.00E-65	945
32	ACIDIC ENDOCHITINASE PRECURSOR	9.00E-45	870
33	ACIDIC ENDOCHITINASE PRECURSOR	9.00E-76	881
34	ACIDIC ENDOCHITINASE PRECURSOR	2.00E-62	881
35	ACIDIC ENDOCHITINASE PRECURSOR	2.00E-63	886
36	ACIDIC ENDOCHITINASE PRECURSOR	9.00E-72	878
37	chitinase [Hevea brasiliensis]	5.00E-40	906
38	chitinase 3-like protein precursor [Trichosanthes kirilowii]	2.00E-73	902
39	chitinase 3-like protein precursor [Trichosanthes kirilowii]	3.00E-80	879
40	ACIDIC ENDOCHITINASE PRECURSOR	6.00E-70	873
41	chitinase [Hevea brasiliensis]	1.00E-48	882
42	ACIDIC ENDOCHITINASE PRECURSOR	2.00E-67	872
43	ACIDIC ENDOCHITINASE PRECURSOR	9.00E-72	890
44	class III chitinase-like protein [Sesbania rostrata]	0.0002	888
45	ACIDIC ENDOCHITINASE PRECURSOR	1.00E-22	892
46	chitinase [Hevea brasiliensis]	2.00E-43	875
47	chitinase 3-like protein precursor [Trichosanthes kirilowii]	4.00E-39	870
48	probable chitinase (EC 3.2.1.14) precursor - cucumber	2.00E-18	863
49	chitinase 3-like protein precursor [Trichosanthes kirilowii]	1.00E-07	875
50	chitinase [Hevea brasiliensis]	1.00E-45	865
51	chitinase 3-like protein precursor [Trichosanthes kirilowii]	6.00E-34	900
52	chitinase [Hevea brasiliensis]	3.00E-67	903
53	chitinase [Psophocarpus tetragonolobus]	2.00E-63	875
54	ACIDIC ENDOCHITINASE PRECURSOR	9.00E-82	864
55	probable chitinase (EC 3.2.1.14) precursor - cucumber	5.00E-14	898
56	chitinase 3-like protein precursor [Trichosanthes kirilowii]	5.00E-69	886
57	chitinase 3-like protein precursor [Trichosanthes kirilowii]	3.00E-39	885
58	ACIDIC ENDOCHITINASE PRECURSOR	4.00E-76	886
59	probable chitinase (EC 3.2.1.14) precursor - cucumber	5.00E-14	902
60	Glycosidase, Chitin Degradation, Multifunctional Enzyme	5.00E-70	900
61	ACIDIC ENDOCHITINASE PRECURSOR	4.00E-75	878
62	ACIDIC ENDOCHITINASE PRECURSOR	1.00E-28	987
63	chitinase [Hevea brasiliensis]	7.00E-40	865
64	ACIDIC ENDOCHITINASE PRECURSOR	2.00E-73	873
65	chitinase [Hevea brasiliensis]	2.00E-50	896
66	probable chitinase (EC 3.2.1.14) precursor - cucumber	1.00E-15	897
67	chitinase [Psophocarpus tetragonolobus]	7.00E-67	889
68	probable chitinase (EC 3.2.1.14) precursor - cucumber	3.00E-14	883
69	chitinase 3-like protein precursor [Trichosanthes kirilowii]	9.00E-40	894
70	chitinase 3-like protein precursor [Trichosanthes kirilowii]	6.00E-09	878
71	chitinase 3-like protein precursor [Trichosanthes kirilowii]	2.00E-25	883
72	probable chitinase (EC 3.2.1.14) precursor - cucumber	5.00E-14	874
73	chitinase [Hevea brasiliensis]	7.00E-52	854
74	chitinase 3-like protein precursor [Trichosanthes kirilowii]	9.00E-40	871
75	chitinase 3-like protein precursor [Trichosanthes kirilowii]	8.00E-40	852
76	probable chitinase (EC 3.2.1.14) precursor - cucumber	2.00E-12	862
77	chitinase 3-like protein precursor [Trichosanthes kirilowii]	2.00E-09	851
78	chitinase [Hevea brasiliensis]	7.00E-42	851
79	chitinase [Hevea brasiliensis]	2.00E-41	847
80	chitinase [Hevea brasiliensis]	4.00E-45	868
81	class III chitinase-like protein [Sesbania rostrata]	0.0004	815
82	chitinase 3-like protein precursor [Trichosanthes kirilowii]	1.00E-19	835
83	chitinase [Cucurbita moschata]	8.00E-07	888
84	probable chitinase (EC 3.2.1.14) precursor - cucumber	3.00E-13	857
85	chitinase 3-like protein precursor [Trichosanthes kirilowii]	2.00E-38	868
86	class III chitinase-like protein [Sesbania rostrata]	7.00E-07	850
87	probable chitinase (EC 3.2.1.14) precursor - cucumber	4.00E-16	867
88	chitinase 3-like protein precursor [Trichosanthes kirilowii]	2.00E-31	854
89	chitinase 3-like protein precursor [Trichosanthes kirilowii]	9.00E-40	859
90	chitinase 3-like protein precursor [Trichosanthes kirilowii]	9.00E-72	861
91	chitinase 3-like protein precursor [Trichosanthes kirilowii]	1.00E-38	873
92	chitinase homolog [Coffea arabica]	4.00E-45	898
93	probable chitinase (EC 3.2.1.14) precursor - cucumber	2.00E-09	902
94	chitinase 3-like protein precursor [Trichosanthes kirilowii]	1.00E-12	851
95	chitinase (EC 3.2.1.14) class III, acidic - soybean	0.0003	849
96	chitinase 3-like protein precursor [Trichosanthes kirilowii]	9.00E-21	859
97	chitinase [Psophocarpus tetragonolobus]	7.00E-79	840
98	probable chitinase (EC 3.2.1.14) precursor - cucumber	5.00E-16	843
99	chitinase 3-like protein precursor [Trichosanthes kirilowii]	2.00E-30	833
100	chitinase [Hevea brasiliensis]	2.00E-50	825
101	probable chitinase (EC 3.2.1.14) precursor - cucumber	2.00E-14	864
102	chitinase 3-like protein precursor [Trichosanthes kirilowii]	2.00E-31	831

103	chitinase 3-like protein precursor [Trichosanthes kirilowii]	1.00E-27	860
104	chitinase [Hevea brasiliensis]	1.00E-58	844
105	chitinase homolog [Coffea arabica]	2.00E-35	879
106	ACIDIC ENDOCHITINASE PRECURSOR	3.00E-75	869
107	ACIDIC ENDOCHITINASE PRECURSOR	1.00E-74	868
108	chitinase [Hevea brasiliensis]	1.00E-43	875
109	chitinase homolog [Coffea arabica]	6.00E-23	867
110	ACIDIC ENDOCHITINASE PRECURSOR	4.00E-68	865
111	probable chitinase (EC 3.2.1.14) precursor - cucumber	1.00E-24	867
112	chitinase 3-like protein precursor [Trichosanthes kirilowii]	3.00E-20	892
113	ACIDIC ENDOCHITINASE PRECURSOR	2.00E-73	861
114	ACIDIC ENDOCHITINASE PRECURSOR	1.00E-71	851
115	chitinase 3-like protein precursor [Trichosanthes kirilowii]	2.00E-74	857
116	chitinase 3-like protein precursor [Trichosanthes kirilowii]	6.00E-60	868
117	chitinase 3-like protein precursor [Trichosanthes kirilowii]	2.00E-62	850
118	chitinase 3-like protein precursor [Trichosanthes kirilowii]	2.00E-34	903
119	chitinase 3-like protein precursor [Trichosanthes kirilowii]	2.00E-32	859
120	chitinase 3-like protein precursor [Trichosanthes kirilowii]	4.00E-38	872
121	chitinase 3-like protein precursor [Trichosanthes kirilowii]	2.00E-20	853
122	chitinase 3-like protein precursor [Trichosanthes kirilowii]	1.00E-25	900
123	chitinase [Hevea brasiliensis]	2.00E-42	860
124	putative chitinase III [Oryza sativa (japonica cultivar-group)]	2.00E-15	858
125	chitinase homologue [Sesbania rostrata]	3.00E-16	894
126	probable chitinase (EC 3.2.1.14) precursor - cucumber	5.00E-11	847
127	chitinase (EC 3.2.1.14) Chib1 - soybean	1.00E-68	863
128	ACIDIC ENDOCHITINASE PRECURSOR	3.00E-57	848
129	chitinase [Hevea brasiliensis]	3.00E-42	886
130	chitinase [Hevea brasiliensis]	1.00E-58	853
**Project Cytochrome p450**			
**ESTs NS1**	**BLAST X**	**E-value**	**Size (bp)**
1	Trans-cinnamate 4-monooxygenase (Cinnamic acid 4-hydroxylase) (CA4H) (C4H) (P450C4H) (Cytochrome P450 73)	2.00E-52	881
2	cytochrome b5 domain-containing protein [Arabidopsis thaliana]	1.00E-10	855
3	cytochrome oxidase deficient homolog 1 [Homo sapiens]	3.00E-44	877
4	cytochrome P450-like protein [Arabidopsis thaliana]	3.00E-76	900
5	desaturase/cytochrome b5 protein [Ricinus communis]	2.00E-60	830
**ESTs RM1**	**BLAST X**	**E-value**	**Size (bp)**
1	pectinesterase family [Arabidopsis thaliana]	2.00E-37	954
2	hypothetical protein At2g36300 [imported] - Arabidopsis thaliana	1.00E-69	952
3	hypothetical protein [Pseudomonas fluorescens PfO-1]	0	870
4	wound induced protein kinase [Nicotiana tabacum]	0	880
5	tsh protein - Escherichia coli	1.00E-40	929
6	PROBABLE TRANSMEMBRANE PROTEIN [Ralstonia solanacearum]	1.00E-76	918
7	hypothetical protein [Ralstonia metallidurans]	0.0001	948
8	DnaJ protein family [Arabidopsis thaliana]	2.00E-89	860
9	hypothetical protein [Pseudomonas syringae pv. syringae B728a]	3.00E-76	941
10	CONSERVED HYPOTHETICAL PROTEIN [Ralstonia solanacearum]	1.00E-52	932
11	hypothetical protein [Burkholderia fungorum]	9.00E-58	958
12	hypothetical protein [Burkholderia fungorum]	8.00E-10	885
13	Photosystem I reaction center subunit II, chloroplast precursor (Photosystem I 20 kDa subunit) (PSI-D) (PS I subunit 5)	1.00E-84	988
14	sensory box histidine kinase/response regulator [Pseudomonas syringae pv. tomato str. DC3000]	1.00E-86	861
15	unknown [Arabidopsis thaliana]	3.00E-69	944
16	putative cytochrome P450 [Arabidopsis thaliana]	2.00E-23	964
17	cytochrome b561 family protein [Pseudomonas syringae pv. tomato str. DC3000]	2.00E-09	945
18	cytochrome b5 domain-containing protein [Arabidopsis thaliana]	3.00E-25	837
19	Thiamine pyrophosphate-requiring enzymes [Thermoanaerobacter tengcongensis]	3.00E-21	901
20	Thiamine pyrophosphate-requiring enzymes [Thermoanaerobacter tengcongensis]	3.00E-21	911
21	cytochrome P450, putative [Arabidopsis thaliana]	2.00E-79	944
22	cytochrome P-450 [Lithospermum erythrorhizon]	3.00E-95	865
23	elicitor-inducible cytochrome P450 [Nicotiana tabacum]	2.00E-73	890
24	NADPH-cytochrome P-450 reductase [Ophiorrhiza pumila]	6.00E-90	949
25	cytochrome BD2, subunit II [Vibrio parahaemolyticus RIMD 2210633]	5.00E-21	1037
26	cytochrome BD2 subunit II [Salmonella typhimurium LT2]	3.00E-35	949
27	cytochrome BD2 subunit II [Salmonella typhimurium LT2]	2.00E-33	951
28	Trans-cinnamate 4-monooxygenase (Cinnamic acid 4-hydroxylase) (CA4H) (C4H) (P450C4H) (Cytochrome P450 73)	3.00E-98	824
29	cytochrome oxidase subunit I [Rhodothermus marinus]	1.00E-35	802
30	cytochrome P450, putative [Arabidopsis thaliana]	4.00E-72	968
31	Cytochrome c-type biogenesis protein [Methanosarcina mazei Goe1]	0.002	981
32	cytochrome b561 family protein [Pseudomonas syringae pv. tomato str. DC3000]	6.00E-08	910
33	cytochrome b561 family protein [Pseudomonas syringae pv. tomato str. DC3000]	3.00E-07	1062
34	Allene oxide synthase, chloroplast precursor (Hydroperoxide dehydrase) (Cytochrome P450 74A)	3.00E-68	928
35	L-lactate dehydrogenase [Escherichia coli K12]	3.00E-52	940
36	L-lactate dehydrogenase [Escherichia coli K12]	2.00E-52	918
37	Cytochrome P450 77A2 (CYPLXXVIIA2) (P-450EG5)	0	953
38	cytochrome P450 [Solanum tuberosum]	2.00E-21	903
39	cytochrome P450 [Solanum tuberosum]	1.00E-52	923
40	cytochrome b561 family protein [Pseudomonas syringae pv. tomato str. DC3000]	9.00E-10	947
41	NADPH-cytochrome P-450 reductase [Ophiorrhiza pumila]	8.00E-06	1090

42	NADPH-cytochrome P-450 reductase [Ophiorrhiza pumila]	2.00E-58	882
43	Cytochrome c-type biogenesis protein [Methanosarcina mazei Goe1]	0.002	895
44	nodulin MtN3 family protein [Arabidopsis thaliana]	2.00E-29	900
45	Cytochrome P450 77A2 (CYPLXXVIIA2) (P-450EG5)	1.00E-74	907
46	putative cytochrome P450 [Arabidopsis thaliana]	8.00E-21	893
47	cytochrome c family protein [Pseudomonas putida KT2440]	1.00E-31	908
48	cytochrome c oxidase subunit (cbb3-type) [Pseudomonas aeruginosa PA01]	3.00E-92	853
49	nitrate reductase 1, cytochrome b(NR), gamma subunit [Shigella flexneri 2a str. 2457T]	6.00E-16	905
50	Cytochrome P450 82A2 (P450 CP4)	4.00E-14	877
51	cytochrome P450 [Solanum tuberosum]	5.00E-64	920
52	cytochrome b561 family protein [Pseudomonas syringae pv. tomato str. DC3000]	4.00E-08	982
53	L-lactate dehydrogenase [Escherichia coli K12]	2.00E-55	866
54	cytochrome P450, putative [Arabidopsis thaliana]	6.00E-65	962
55	putative cytochrome P450 [Solanum tuberosum]	2.00E-06	853
56	60S RIBOSOMAL PROTEIN L10-3 (QM/R22)	0	948
57	cytochrome b5 domain-containing protein [Arabidopsis thaliana]	1.00E-36	942
58	NADPH-ferrihemoprotein reductase (EC 1.6.2.4) 1 - parsley	3.00E-17	1092
59	cytochrome P450 [Petunia x hybrida]	3.00E-62	938
60	heme exporter protein C; cytochrome C-type biogenesis protein [Xanthomonas axonopodis pv. citri str. 306]	6.00E-44	930
61	Allene oxide synthase, chloroplast precursor (Hydroperoxide dehydrase) (Cytochrome P450 74A)	5.00E-64	933
62	NADPH-cytochrome P-450 reductase [Ophiorrhiza pumila]	5.00E-53	955
63	probable thiol:disulfide interchange protein [Pseudomonas aeruginosa PA01]	2.00E-52	952
64	Cytochrome c-type biogenesis protein [Methanosarcina mazei Goe1]	0.002	963
65	allene oxide synthase / cytochrome P450 74A [Arabidopsis thaliana]	3.00E-81	934
66	putative cytochrome B 561 [Craterostigma plantagineum]	4.00E-38	829
67	probable cytochrome c [Nitrosomonas europaea ATCC 19718]	2.00E-42	859
68	allene oxide synthase / cytochrome P450 74A [Arabidopsis thaliana]	3.00E-06	908
69	NADPH-cytochrome P450 oxidoreductase (EC 1.-.-.-) - common tobacco	3.00E-85	1000
70	expressed protein [Arabidopsis thaliana]	3.00E-23	936
**ESTs RX1**	**BLAST X**	**E-value**	**Size (bp)**
1	No Hits Found	0	894
2	No Hits Found	0	902
3	No Hits Found	0	894
4	No Hits Found	0	870
5	No Hits Found	0	930
6	No Hits Found	0	962
7	No Hits Found	0	988
8	No Hits Found	0	955
9	No Hits Found	0	923
10	No Hits Found	0	919
11	No Hits Found	0	897
12	No Hits Found	0	847
13	No Hits Found	0	965
14	No Hits Found	0	891
15	No Hits Found	0	963
16	60S RIBOSOMAL PROTEIN L10-3 (QM/R22)	0	968
17	NADPH-cytochrome P450 reductase (CPR) (P450R)	1.00E-22	945
18	cytochrome P450 -related [Arabidopsis thaliana]	4.00E-06	968
19	cytochrome b5 domain-containing protein [Arabidopsis thaliana]	6.00E-18	989
20	putative cytochrome c oxidoreductase [Arabidopsis thaliana]	5.00E-23	956
21	cytochrome P450 [Solanum tuberosum]	5.00E-62	959
22	cytochrome P450 monooxygenase CYP72A5 [Zea mays subsp. mays]	5.00E-33	958
23	cytochrome P450 [Solanum tuberosum]	3.00E-28	981
24	cytochrome P450, putative [Arabidopsis thaliana]	5.00E-49	978
25	cytochrome c551 peroxidase, putative [Pseudomonas putida KT2440]	6.00E-06	942
26	cytochrome b5 [Arabidopsis thaliana]	5.00E-26	891
27	cytochrome P450 [Catharanthus roseus]	2.00E-17	1017
28	NADPH-cytochrome P-450 reductase [Ophiorrhiza pumila]	3.00E-50	881
29	fatty acid desaturase/cytochrome b5 fusion protein [Arabidopsis thaliana]	5.00E-42	826
30	putative cytochrome c oxidoreductase [Arabidopsis thaliana]	4.00E-18	899
31	allene oxide synthase / cytochrome P450 74A [Arabidopsis thaliana]	6.00E-62	915
32	Ubiquinol-cytochrome C reductase complex 14 kDa protein (CR14)	9.00E-37	924
33	cytochrome c551 peroxidase, putative [Pseudomonas putida KT2440]	7.00E-06	982
34	cytochrome P450 ent-kaurene oxidase (GA3) [Arabidopsis thaliana]	8.00E-62	958
35	Allene oxide synthase, chloroplast precursor (Hydroperoxide dehydrase) (Cytochrome P450 74A)	8.00E-65	943
36	putative cytochrome P450 [Arabidopsis thaliana]	9.00E-72	938
37	Putative Cytochrome P450 [Oryza sativa]	1.00E-39	891
38	Allene oxide synthase (Rubber particle protein) (RPP)	2.00E-23	916
39	cytochrome P450 ent-kaurene oxidase (GA3) [Arabidopsis thaliana]	4.00E-79	955
40	Cytochrome c	2.00E-56	969
41	Allene oxide synthase, chloroplast precursor (Hydroperoxide dehydrase) (Cytochrome P450 74A)	3.00E-75	929
42	cytochrome c551 peroxidase, putative [Pseudomonas putida KT2440]	6.00E-06	928
43	cytochrome P450 [Solanum tuberosum]	1.00E-28	992
44	expressed protein [Arabidopsis thaliana]	9.00E-16	839
**ESTs SS1**	**BLAST X**	**E-value**	**Size (bp)**
1	No Hits Found	0	889
2	Ubiquinol-cytochrome C reductase complex 14 kDa protein (CR14)	9.00E-37	854

3	putative NADPH-cytochrome P450 reductase [Pisum sativum]	3.00E-14	838
4	Ubiquinol-cytochrome C reductase complex 14 kDa protein (CR14)	2.00E-16	875
5	putative cytochrome c oxidoreductase [Arabidopsis thaliana]	3.00E-40	871
**Project Glucanase**			
**ESTs NS1**	**BLAST X**	**E-value**	**Size (bp)**
1	glycosyl hydrolase family 17 [Arabidopsis thaliana]	2.00E-21	875
2	putative glucanase [Oryza sativa (japonica cultivar-group)]	4.00E-14	832
3	probable glucanase - Arabidopsis thaliana	2.00E-37	893
**ESTs RM1**	**BLAST X**	**E-value**	**Size (bp)**
1	xyloglucan endotransglycosylase, putative [Arabidopsis thaliana]	2.00E-68	962
2	putative beta-1, 3-glucanase [Oryza sativa (japonica cultivar-group)]	1.00E-58	910
3	Glucan endo-1, 3-beta-glucosidase, acidic isoform PR-Q' precursor ((1->3)-beta-glucan endohydrolase) ((1->3)-beta-glucanase) (Beta-1, 3-endoglucanase) (PR-35)	5.00E-80	892
4	glycosyl hydrolase family 17 (beta-1, 3-glucanase) [Arabidopsis thaliana]	3.00E-45	938
5	glucan endo-1, 3-beta-D-glucosidase (EC 3.2.1.39) [imported] - garden pea	2.00E-25	918
6	glycosyl hydrolase family 17 [Arabidopsis thaliana]	4.00E-65	854
7	glucan endo-1, 3-beta-D-glucosidase (EC 3.2.1.39) [imported] - garden pea	2.00E-76	877
8	xyloglucan endotransglycosylase, putative [Arabidopsis thaliana]	3.00E-60	941
9	putative beta-1, 3-glucanase [Oryza sativa (japonica cultivar-group)]	1.00E-71	877
10	glycosyl hydrolase family 9 [Arabidopsis thaliana]	9.00E-56	935
11	glucan endo-1, 3-beta-D-glucosidase (EC 3.2.1.39) [imported] - garden pea	3.00E-42	852
12	glycosyl hydrolase family 9 [Arabidopsis thaliana]	2.00E-56	959
13	1, 3-beta-glucanase (EC 3.2.1.-), basic - tomato	4.00E-73	1009
14	endo-beta-1, 4-D-glucanase [Lycopersicon esculentum]	3.00E-81	925
15	xyloglucan endo-1, 4-beta-D-glucanase (EC 3.2.1.-) F6H11.140 - Arabidopsis thaliana	2.00E-08	1077
16	putative beta-1, 3-glucanase [Oryza sativa (japonica cultivar-group)]	6.00E-86	935
17	xyloglucan endo-1, 4-beta-D-glucanase (EC 3.2.1.-) F6H11.140 - Arabidopsis thaliana	3.00E-07	895
18	xyloglucan endotransglycosylase, putative [Arabidopsis thaliana]	2.00E-13	853
19	xyloglucan endo-1, 4-beta-D-glucanase (EC 3.2.1.-) F6H11.140 - Arabidopsis thaliana	3.00E-50	901
20	xyloglucan endotransglycosylase, putative [Arabidopsis thaliana]	1.00E-07	801
21	putative exoglucanase precursor [Oryza sativa]	6.00E-56	879
22	xyloglucan endo-1, 4-beta-D-glucanase (EC 3.2.1.-) F6H11.140 - Arabidopsis thaliana	4.00E-34	935
**ESTs RX1**	**BLAST X**	**E-value**	**Size (bp)**
1	glycosyl hydrolase family 17 [Arabidopsis thaliana]	2.00E-20	886
2	xyloglucan endo-1, 4-beta-D-glucanase (EC 3.2.1.-) (clone NXG1) - common nasturtium	3.00E-93	921
3	glucanase -related [Arabidopsis thaliana]	6.00E-19	982
4	contains similarity to endo-1, 3-1, 4-beta-D-glucanase~gene_id:MDB19.5 [Arabidopsis thaliana]	3.00E-18	999
5	1, 3-beta-glucanase (EC 3.2.1.-), acidic - tomato	7.00E-59	970
6	endo-1, 3-beta-glucanase-like protein [Pyrus pyrifolia]	1.00E-84	946
**ESTs SS1**	**BLAST X**	**E-value**	**Size (bp)**
1	beta-1, 3 glucanase-like protein [Oryza sativa (japonica cultivar-group)]	2.00E-20	874
2	glucan endo-1, 3-beta-D-glucosidase (EC 3.2.1.39) [imported] - garden pea	5.00E-76	876
**Project HSP**			
**ESTs RM1**	**BLAST X**	**E-value**	**Size (bp)**
1	tetratricopeptide repeat (TPR)-containing protein [Arabidopsis thaliana]	1.00E-41	984
2	tetratricopeptide repeat (TPR)-containing protein [Arabidopsis thaliana]	9.00E-44	927
3	heat shock protein hsp70	7.00E-72	880
**ESTs RX1**	**BLAST X**	**E-value**	**Size (bp)**
1	heat shock protein family [Arabidopsis thaliana]	4.00E-34	977
**ESTs SS1**	**BLAST X**	**E-value**	**Size (bp)**
1	heat shock protein hsp70b [Arabidopsis thaliana]	0.0004	909
2	cytosolic class I small heat-shock protein HSP17.5 [Castanea sativa]	2.00E-41	892
3	tetratricopeptide repeat (TPR)-containing protein [Arabidopsis thaliana]	1.00E-21	855
4	17.6 KD CLASS I HEAT SHOCK PROTEIN (HSP 17.6-L)	7.00E-05	853
**Project Thaumatin**			
**ESTs RX1**	**BLAST X**	**E-value**	**Size (bp)**
1	putative thaumatin-like protein [Vitis vinifera]	2.00E-10	995
2	thaumatin-like protein [Vitis vinifera]	1.00E-89	991
**ESTs SS1**	**BLAST X**	**E-value**	**Size (bp)**
1	putative thaumatin [Arabidopsis thaliana]	1.00E-95	864
**Project Chalconesynthase**			
**ESTs RM1**	**BLAST X**	**E-value**	**Size (bp)**
1	CHALCONE SYNTHASE 2 (NARINGENIN-CHALCONE SYNTHASE 2)	2.00E-59	915
2	CHALCONE SYNTHASE 2 (NARINGENIN-CHALCONE SYNTHASE 2)	0	838
3	CHALCONE SYNTHASE 2 (NARINGENIN-CHALCONE SYNTHASE 2)	0	936
**Project Pathogenesis**			
**ESTs RM1**	**BLAST X**	**E-value**	**Size (bp)**
1	subtilisin-like proteinase (EC 3.4.21.-) precursor P69B, pathogenesis-related - tomato	4.00E-09	958
2	Similar to Arabidopsis thaliana putative pathogenesis-related protein (U20347) [Oryza sativa (japonica cultivar-group)]	2.00E-16	838
**ESTs RX1**	**BLAST X**	**E-value**	**Size (bp)**
1	pathogenesis-related protein 1 - parsley	9.00E-06	986
2	pathogenesis-related protein 10 [Vitis vinifera]	2.00E-36	966
3	pathogenesis-related protein 10 [Vitis vinifera]	5.00E-32	945
4	Acidic endochitinase Q precursor (Pathogenesis-related protein Q) (PR-Q)	3.00E-13	967
5	Pathogenesis-related protein R major form precursor (Thaumatin-like protein E22)	2.00E-09	995
6	pathogenesis-related protein [Oryza sativa]	2.00E-28	972
7	pathogenesis-related protein 5-1 [Helianthus annuus]	5.00E-86	991
**ESTs SS1**	**BLAST X**	**E-value**	**Size (bp)**

1	thaumatin family [Arabidopsis thaliana]	4.00E-48	864
	**Project Polyphenoloxidase**		
**ESTs SS1**	**BLAST X**	**E-value**	**Size (bp)**
1	catechol oxidase; polyphenol oxidase [Ipomoea batatas]	3.00E-71	893
	**Project Importin**		
**ESTs NS1**	**BLAST X**	**E-value**	**Size (bp)**
1	importin alpha subunit [Arabidopsis thaliana]	7.00E-12	901
**ESTs RM1**	**BLAST X**	**E-value**	**Size (bp)**
1	importin alpha 2 [Capsicum annuum]	5.00E-96	925
2	cellular apoptosis susceptibility protein (importin-alpha re-exporter), putative [Arabidopsis thaliana]	1.00E-33	1048
3	rubisco small subunit [Coffea arabica]	1.00E-93	924
4	rubisco small subunit [Coffea arabica]	1.00E-89	909
5	rubisco small subunit [Coffea arabica]	1.00E-93	888
6	rubisco small subunit [Coffea arabica]	1.00E-93	961
7	rubisco small subunit [Coffea arabica]	3.00E-91	907
8	rubisco small subunit [Coffea arabica]	4.00E-93	912
9	rubisco small subunit [Coffea arabica]	1.00E-93	989
10	rubisco small subunit [Coffea arabica]	1.00E-93	962
11	rubisco small subunit [Coffea arabica]	1.00E-93	903
12	rubisco small subunit [Coffea arabica]	1.00E-93	902
13	rubisco small subunit [Coffea arabica]	1.00E-93	974
14	rubisco small subunit [Coffea arabica]	1.00E-93	847
**ESTs RX1**	**BLAST X**	**E-value**	**Size (bp)**
1	AtKAP alpha [Arabidopsis thaliana]	5.00E-94	908
2	rubisco small subunit [Coffea arabica]	7.00E-93	939
3	rubisco small subunit [Coffea arabica]	1.00E-93	957
4	No Hits Found	0	905
5	No Hits Found	0	941
6	No Hits Found	0	892
7	No Hits Found	0	914
8	rubisco small subunit [Coffea arabica]	1.00E-90	946
9	rubisco small subunit [Coffea arabica]	6.00E-78	980
10	No Hits Found	0	927
11	No Hits Found	0	883
12	No Hits Found	0	849
13	No Hits Found	0	937
14	No Hits Found	0	886
15	No Hits Found	0	902
**ESTs SS1**	**BLAST X**	**E-value**	**Size (bp)**
1	probable nuclear transport factor importin alpha [imported] - Arabidopsis thaliana	2.00E-13	853
2	rubisco small subunit [Coffea arabica]	1.00E-93	846
3	rubisco small subunit [Coffea arabica]	1.00E-93	884
4	rubisco small subunit [Coffea arabica]	2.00E-54	869
	**Project Glucosyltransferase**		
**ESTs NS1**	**BLAST X**	**E-value**	**Size (bp)**
1	UDP-glucose:salicylic acid glucosyltransferase [Nicotiana tabacum]	1.00E-75	881
2	putative glucosyltransferase [Lycopersicon esculentum]	9.00E-25	802
3	Sucrose synthase (Sucrose-UDP glucosyltransferase)	0	904
**ESTs RM1**	**BLAST X**	**E-value**	**Size (bp)**
1	Sucrose synthase (Sucrose-UDP glucosyltransferase) (SS65)	3.00E-93	926
2	glucosyltransferase-8 [Vigna angularis]	0.006	928
3	Sucrose synthase (Sucrose-UDP glucosyltransferase) (SS65)	3.00E-98	911
4	Putative indole-3-acetate beta-glucosyltransferase [Oryza sativa (japonica cultivar-group)]	1.00E-35	860
5	Sucrose synthase (Sucrose-UDP glucosyltransferase) (SS65)	0	929
6	glycosyltransferase family [Arabidopsis thaliana]	4.00E-42	871
7	glucosyl transferase [Xanthomonas axonopodis pv. citri str. 306]	6.00E-57	917
8	probable endo-xyloglucan transferase - upland cotton (fragment)	2.00E-64	941
9	alpha2-glucosyltransferase [Homo sapiens]	3.00E-29	942
10	glucosyltransferase NTGT3 [Nicotiana tabacum]	1.00E-53	960
11	probable endo-xyloglucan transferase - upland cotton (fragment)	3.00E-70	962
12	probable endo-xyloglucan transferase - upland cotton (fragment)	4.00E-37	935
13	alpha, alpha-trehalose-phosphate synthase, UDP-forming, putative [Arabidopsis thaliana]	5.00E-73	927
14	alpha, alpha-trehalose-phosphate synthase, UDP-forming, putative [Arabidopsis thaliana]	8.00E-32	1025
15	probable endo-xyloglucan transferase - upland cotton (fragment)	9.00E-12	1077
16	alpha, alpha-trehalose-phosphate synthase, UDP-forming, putative [Arabidopsis thaliana]	3.00E-90	941
17	glycosyltransferase family 2 [Arabidopsis thaliana]	0	962
18	Sucrose synthase (Sucrose-UDP glucosyltransferase) (SS65)	0	858
19	glycosyltransferase family 2 [Arabidopsis thaliana]	5.00E-22	964
20	probable endo-xyloglucan transferase - upland cotton (fragment)	0	918
21	glycosyltransferase family 20 [Arabidopsis thaliana]	2.00E-53	921
22	Limonoid UDP-glucosyltransferase (Limonoid glucosyltransferase) (Limonoid GTase) (LGTase)	6.00E-78	936
23	alpha, alpha-trehalose-phosphate synthase, UDP-forming, putative [Arabidopsis thaliana]	1.00E-68	878
24	probable endo-xyloglucan transferase - upland cotton (fragment)	4.00E-55	901
25	hypothetical protein [Pseudomonas syringae pv. syringae B728a]	4.00E-62	1012
26	ribosomal protein L7	1.00E-29	916
27	theobromine synthase [Coffea arabica]	0	924
28	expressed protein [Arabidopsis thaliana]	7.00E-64	828

29	auxin-regulated protein [Arabidopsis thaliana]	9.00E-79	940
30	putative protein [Arabidopsis thaliana]	7.00E-96	951
31	secretory peroxidase [Avicennia marina]	0	936
32	polygalacturonase (EC 3.2.1.15) 1 beta chain precursor - tomato	2.00E-69	999
33	No Hits Found	0	861
34	DEAD box RNA helicase, putative [Arabidopsis thaliana]	0	880
35	expressed protein [Arabidopsis thaliana]	4.00E-14	891
36	No Hits Found	0	855
37	GENERAL L-AMINO ACID TRANSPORT ATP-BINDING PROTEIN AAPP [Brucella melitensis]	4.00E-61	888
38	expressed protein [Arabidopsis thaliana]	3.00E-69	879
39	No Hits Found	0	947
40	No Hits Found	0	918
41	O-sialoglycoprotein endopeptidase [Pseudomonas putida KT2440]	4.00E-66	937
42	sensor histidine kinase [Pseudomonas putida KT2440]	1.00E-09	918
43	DHHC-type zinc finger domain-containing protein [Arabidopsis thaliana]	0	952
44	hypothetical protein [Pseudomonas fluorescens PfO-1]	4.00E-83	924
45	protein phosphatase 2C (PP2C), putative [Arabidopsis thaliana]	1.00E-24	966
46	expressed protein [Arabidopsis thaliana]	1.00E-49	958
47	hypothetical protein [Pseudomonas aeruginosa UCBPP-PA14]	4.00E-08	947
48	hypothetical protein [Azotobacter vinelandii]	7.00E-52	940
49	hypothetical protein [Pseudomonas fluorescens PfO-1]	9.00E-33	870
50	alpha subunit of aromatic ring hydroxylase component of chlorobenzoate 1, 2-dioxygenase [Burkholderia sp. NK8]	4.00E-94	964
**ESTs RX1**	**BLAST X**	**E-value**	**Size (bp)**
1	Sucrose synthase (Sucrose-UDP glucosyltransferase)	0	957
2	Sucrose synthase (Sucrose-UDP glucosyltransferase) (SS65)	0	918
3	Sucrose synthase (Sucrose-UDP glucosyltransferase) (SS65)	0	983
4	Sucrose synthase (Sucrose-UDP glucosyltransferase)	0	939
5	glucosyltransferase [Nicotiana tabacum]	6.00E-50	932
6	hypothetical protein T16E15.1 - Arabidopsis thaliana	4.00E-17	943
7	glycosyltransferase family 20 [Arabidopsis thaliana]	0	974
8	Sucrose synthase (Sucrose-UDP glucosyltransferase)	0	929
9	glucuronosyl transferase homolog, ripening-related - tomato (fragment)	4.00E-77	946
10	Sucrose synthase (Sucrose-UDP glucosyltransferase)	0	929
11	Hydroquinone glucosyltransferase (Arbutin synthase)	2.00E-48	945
12	indole-3-acetate beta-glucosyltransferase -related [Arabidopsis thaliana]	3.00E-24	890
13	Sucrose synthase (Sucrose-UDP glucosyltransferase)	2.00E-79	895
14	glucosyltransferase NTGT2 [Nicotiana tabacum]	3.00E-31	877
15	glycosyltransferase family [Arabidopsis thaliana]	2.00E-38	883
16	alpha, alpha-trehalose-phosphate synthase, UDP-forming (trehalose-6-phosphate synthase/UDP-glucose-glucosephosphate glucosyltransferase), putative [Arabidopsis thaliana]	2.00E-54	936
17	No Hits Found	0	984
18	No Hits Found	0	954
19	No Hits Found	0	910
20	No Hits Found	0	893
21	No Hits Found	0	891
22	No Hits Found	0	891
23	No Hits Found	0	869
24	No Hits Found	0	951
25	No Hits Found	0	896
26	No Hits Found	0	920
27	No Hits Found	0	855
28	No Hits Found	0	943
**ESTs SS1**	**BLAST X**	**E-value**	**Size (bp)**
1	putative glucosyltransferase [Arabidopsis thaliana]	2.00E-33	850
2	No Hits Found	0	903
