# Supplementary material for: In silico identification of coffee genome expressed sequences potentially associated with resistance to diseases
Source: Genet Mol Biol. 2010 Dec 1;33(4):795–806. doi: 10.1590/s1415-47572010000400031 (PMC3036153; doi:10.1590/s1415-47572010000400031)
Supplement: Figure S1 — Distribution of the number of reads in the 140 EST-contigs with E-values < e-20 and scores > 100 formed after clustering. [file gmb-33-4-795-suppl16.pdf]

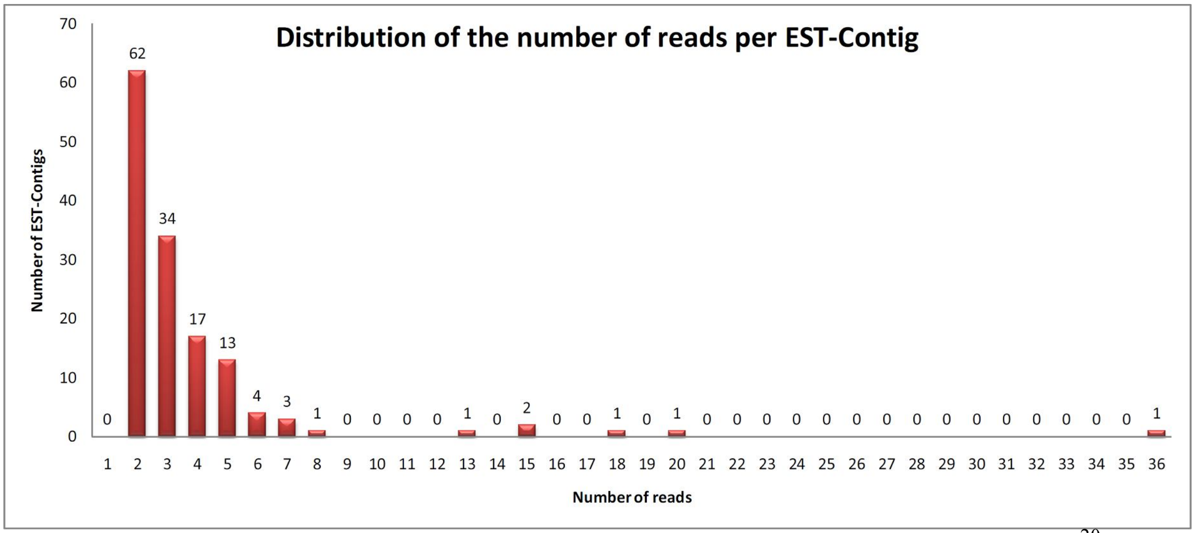


**Figure S1:** Distribution of the number of reads in the 140 EST-Contigs with e-value < $e^{-20}$ and score > 100 formed after clustering.
