# Supplementary figures and images for: In silico identification of coffee genome expressed sequences potentially associated with resistance to diseases
Source: Genet Mol Biol. 2010 Dec 1;33(4):795–806. doi: 10.1590/s1415-47572010000400031 (PMC3036153; doi:10.1590/s1415-47572010000400031)

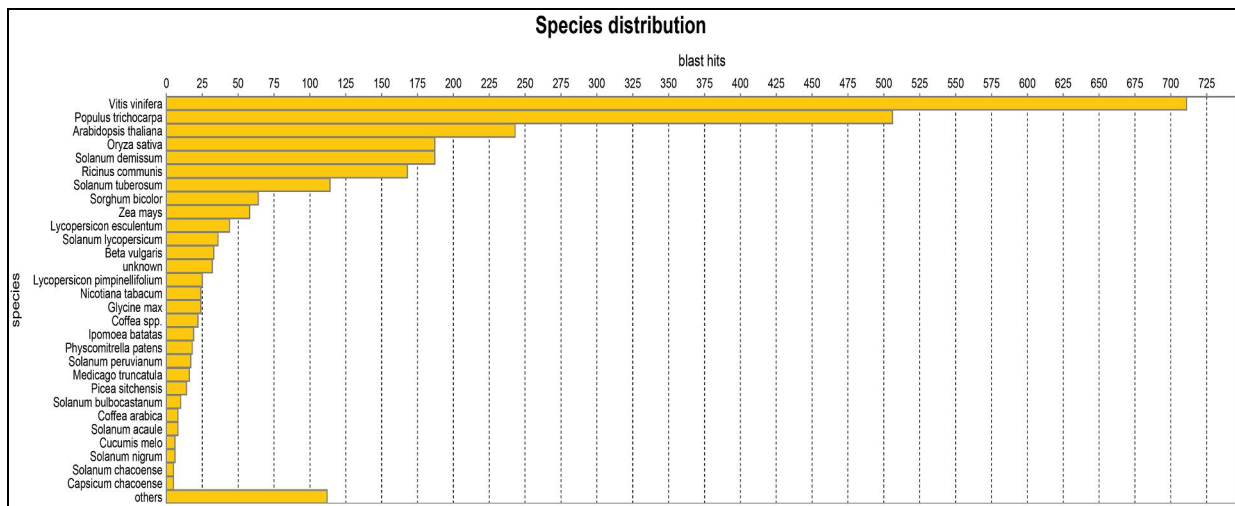

**Figure S2:** Species with most blast hits with the EST-Contigs

Supplement: Figure S2 — Species with the most blast hits with the EST-contigs. [file gmb-33-4-795-suppl17.pdf]
