# Supplementary material for: In silico identification of coffee genome expressed sequences potentially associated with resistance to diseases
Source: Genet Mol Biol. 2010 Dec 1;33(4):795–806. doi: 10.1590/s1415-47572010000400031 (PMC3036153; doi:10.1590/s1415-47572010000400031)
Supplement: Figure S4 — Average similarity value distribution of the 20 best hits of the 140 EST-contigs submitted to BlastX by Blast2GO. [file gmb-33-4-795-suppl19.pdf]

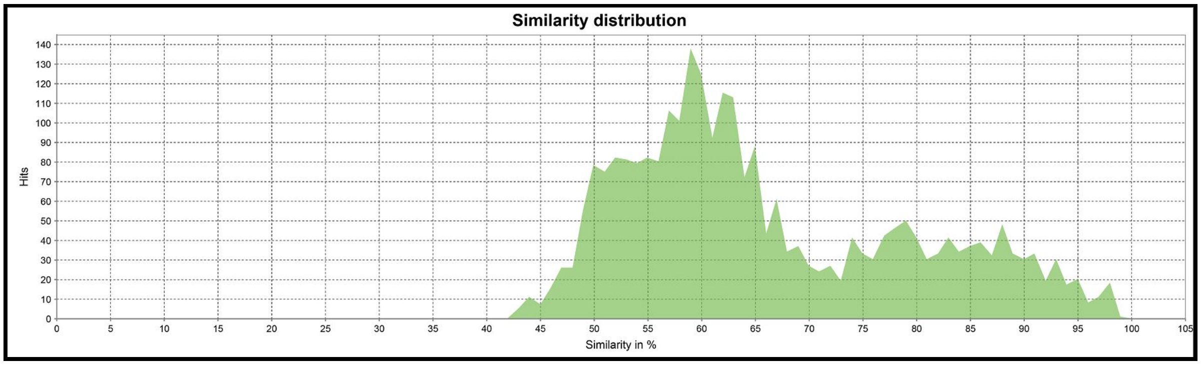

Figure S4: Average similarity value distribution of the best 20 hitsof the 140 EST-Contigs submitted to BlastX by Blast2GO
