# Supplementary material for: In silico identification of coffee genome expressed sequences potentially associated with resistance to diseases
Source: Genet Mol Biol. 2010 Dec 1;33(4):795–806. doi: 10.1590/s1415-47572010000400031 (PMC3036153; doi:10.1590/s1415-47572010000400031)
Supplement: Figure S5 — Distribution of the number of GO terms (Cellular Component, Molecular Function and Biological Process) for the 140 EST-contigs analyzed by Blast2GO. [file gmb-33-4-795-suppl20.pdf]

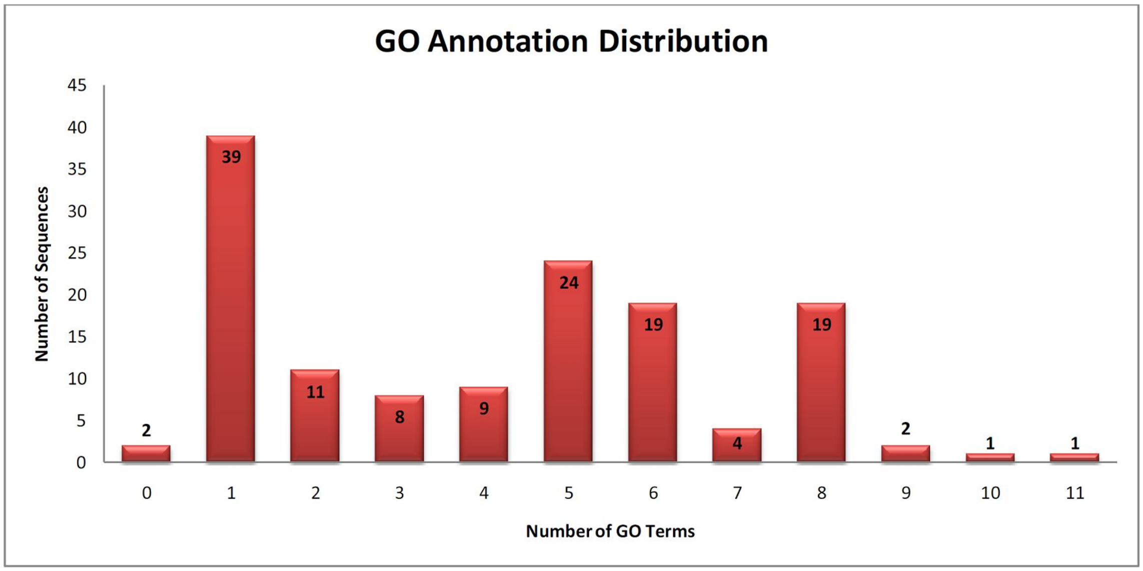

Figure S5: Distribution of the number of GO terms (Cellular Component, Molecular Function and Biological Process) from the 140 EST-Contigs analyzed by Blast2GO.
