# Supplementary material for: In silico identification of coffee genome expressed sequences potentially associated with resistance to diseases
Source: Genet Mol Biol. 2010 Dec 1;33(4):795–806. doi: 10.1590/s1415-47572010000400031 (PMC3036153; doi:10.1590/s1415-47572010000400031)
Supplement: Figure S6 — Evidence code (EC) distribution for the EST-contig blast hits. [file gmb-33-4-795-suppl21.pdf]

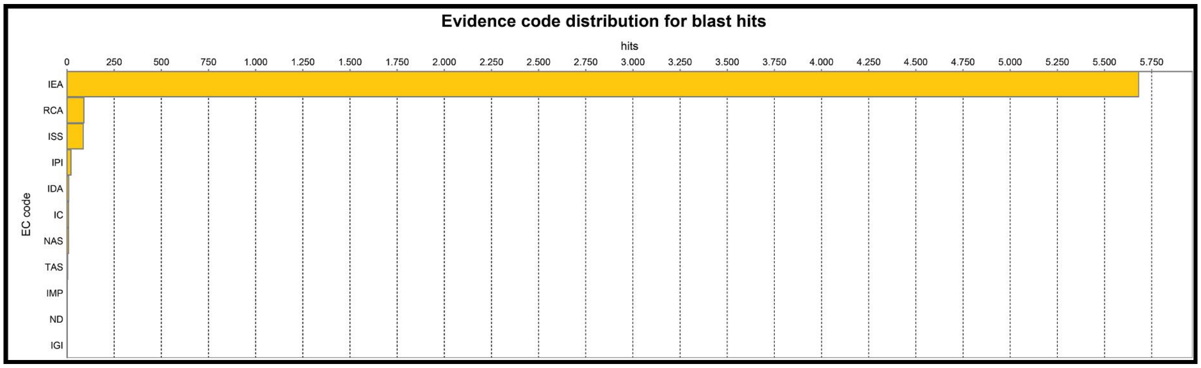

Figure S6: EC (Evidence Code) distribution for the EST-Contigs blast hits. IEA = Inferred from Electronic Annotation. ISS = Inferred from Sequence or Structural Similarity. RCA = Inferred from Reviewed Computational Analysis. IPI = Inferred from Physical Interaction. IDA = Inferred from Direct Assay. IC = Inferred by Curator. NAS = Non-traceable Author Statement. TAS = Traceable Author Statement. ND = No biological Data available. IMP = Inferred from Mutant Phenotype. IGI = Inferred from Genetic Interaction.
